# Biomimetic Synthesis of Azorellolide via Cyclopropylcarbinyl Cation Chemistry

**DOI:** 10.1021/jacs.4c14664

**Published:** 2024-12-18

**Authors:** Jordan Y. Artzy, Dean J. Tantillo, Dirk H. Trauner

**Affiliations:** § Department of Chemistry, University of Pennsylvania, Philadelphia, Pennsylvania 19104, United States; ‡ Department of Chemistry, University of California, Davis, California 95616, United States

## Abstract

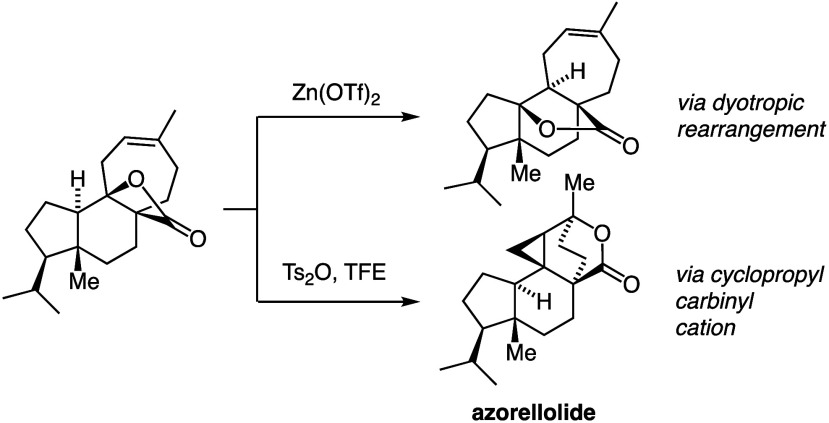

A concise synthesis of the complex diterpene azorellolide, inspired by speculations on biosynthetic cationic cascades, is presented. The approach, guided by computation, relies on the intramolecular interception of a cyclopropylcarbinyl cation by an appended carboxylate. The successful execution of this strategy was achieved through acid-catalyzed isomerization of a β-lactone in competition with a type I dyotropic rearrangement.

Biomimetic synthesis can be defined as an attempt to emulate biosynthetic pathways and patterns in the absence of biological catalysts. Although this effort may seem naïve given the sophistication of enzymes it has proven to be remarkably successful.^1,2^ This is especially true when utilizing reaction cascades where numerous bonds are formed sequentially from energetically spring-loaded precursors. Examples include cascades that involve pericyclic reactions, such as electrocyclizations and cycloadditions, which can lead to impressive increases in stereochemical and topological complexity.^3,4^ Others feature cyclizations of linear polyolefin precursors through cationic cascades that rapidly establish complex polycyclic terpenoid frameworks, mimicking their biosynthesis.^5−8^ These reactions are all the more remarkable as terpene synthases not only conformationally preorganize polyenyl cations toward cyclizations, as well as hydride and alkyl shifts, but also quench the terminal cation through selective attack of nucleophiles or deprotonations.^9^

We have long been fascinated by polyolefin cyclizations that converge on cyclopropylcarbinyl (CC)/cyclobutyl (CB)/homoallyl (HA) cations. Such carbocations have been famously formulated in the biosynthesis of squalene^10^ but have been little studied in the final phase of biosynthetic cyclizations.^11,12^ They have numerous applications in elegant total syntheses.^13,14^ In principle, such cations can exist as rapidly interconverting discrete species or represent resonance structures (bicyclobutonium ions).^15,16^ If all four of their carbon atoms are distinguishable, the reactivity of these cations with nucleophiles or bases can be dauntingly complex (Figure 1A). Up to three CC, three CB, and six HA cations can be formulated that lead to discrete regioisomers upon nucleophilic interception, further complicated by the possibility of stereoisomers (see Supporting Information (SI) Figure 1 for a full enumeration). In Nature, the selective interception of CC/CB/HA cations is achieved by terpene synthases that position a water nucleophile in a defined trajectory, which may be difficult to mimic with less complex catalysts. To overcome this challenge, we reasoned that a high degree of selectivity could be achieved through intramolecularization (Figure 1B). We now report a synthesis of the diterpenoid azorellolide that hinges on such an intramolecularization strategy.

**Figure 1:**
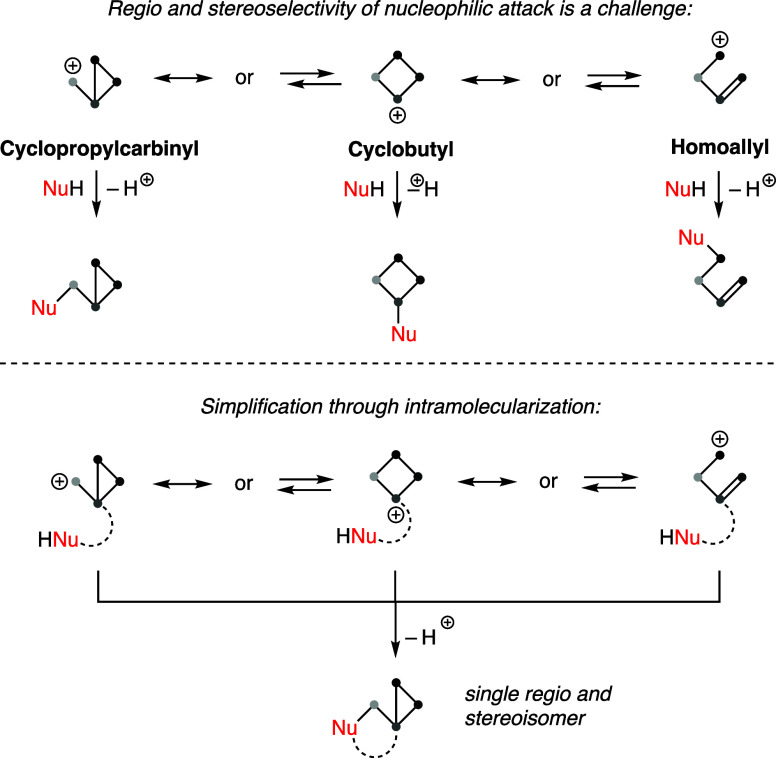
A simplified view of the CC/CB/HA manifold and its interception with nucleophiles. (A) The regio and stereoselective attack of a nucleophile presents selectivity challenges. This challenge can be met through intramolecularization. (B) CC = cyclopropylcarbinyl cation; CB = cyclobutyl cation; HA = homoallyl cation.

Azorellolide belongs to a family of natural products isolated from flowering plants of the genus *Azorella*, which grow in high altitudes in the southern hemisphere (Figure 2A).^17^ Structurally, this diterpene features a 5/6/6/3 skeleton with an additional δ-lactone that contains seven stereogenic centers, three of which are quaternary. Other members of the family are dihydroazorellolide, 13α/β hydroxy azorellane, and azorellanol.^17−19^ The azorellanes are often isolated with mulinane diterpenes, such as mulinenic acid or isomulinic acid, which are characterized by an oxidized 5/6/7 framework.^20,21^ A number of mulinanes and azorellanes have shown gastroprotective, antibacterial, antiprotozoal, and cytotoxic activities.^22^

**Figure 2:**
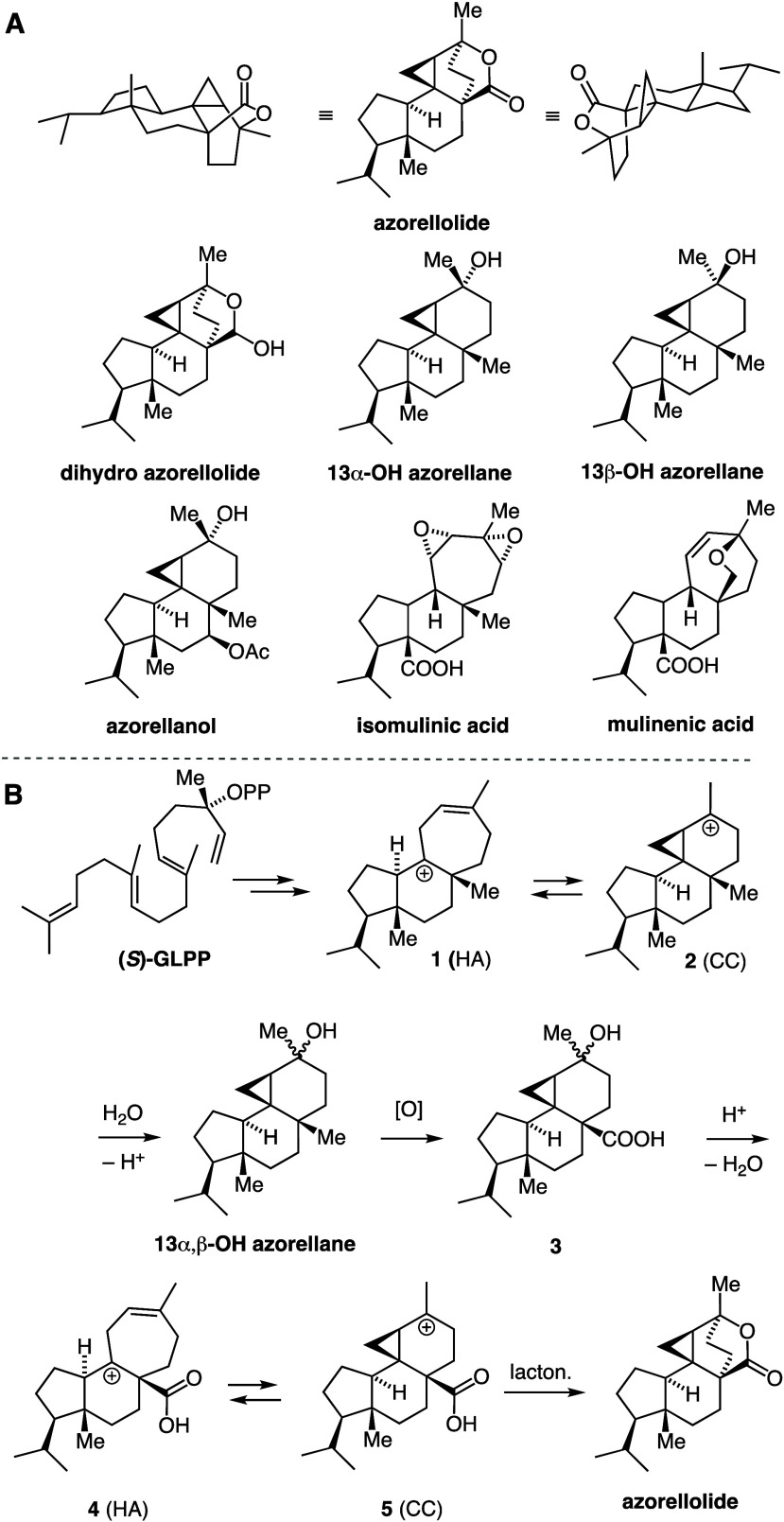
(A) Selected azorellanes and mulinanes. (B) Proposed endgame in the biosynthesis of azorellolide.

A proposed biosynthetic endgame of azorellolide is shown in Figure 2B. Cyclization of geranyl-linalyl pyrophosphate (GLPP) with methyl and hydride shifts could afford HA cation **1**, which could be in equilibrium with CC cation **2**. Interception of the latter with water affords 13α and/or 13β azorellane.^22^ Oxidation of an angular methyl group would yield carboxylic acid **3**, which then undergoes ionization to HA cation **4** in equilibrium with CC cation **5**. Quenching of the latter by the carboxylate leads to azorellolide.

We evaluated the interconversion of the terminal cations and their nucleophilic interception with quantum mechanical calculations (relative free energies in kcal/mol; mPW1PW91/6-31+G(d,p);^23−29^ see SI for additional details). HA cation **4** was found to be +5 kcal/mol higher in energy that CC cation **5**, whose calculated structure is shown at the center (note the delocalization in the cyclopropyl unit reflected by the bond lengths) (Figure 3). A CB cation was not located. Engagement of the carbonyl group in **5** leads to protonated azorellolide **6**, the lowest energy carbocation found (Δ*G*_rel_ = −14.8). By comparison, protonated β-lactone **7** formed through nucleophilic interception of **4** is higher in energy than its precursor (Δ*G*_rel_ = +3.1). The transition states leading from **4** to **5** and from **5** to **6** were found to be low lying, which would result in instantaneous interconversion of these species at room temperature. We also calculated the relative energies of four possible products, namely β-lactones **8** and **9** (stemming from HA ions), the δ-lactone **10** (stemming from a hypothetical CB ion), and the δ-lactone azorellolide. Among these, azorellolide is by far the lowest in energy. Interestingly a transition state that directly leads from **5** to the protonated form of β-lactone **9** most closely resembles a bicyclobutonium ion (see SI Figure 2 for details).

**Figure 3:**
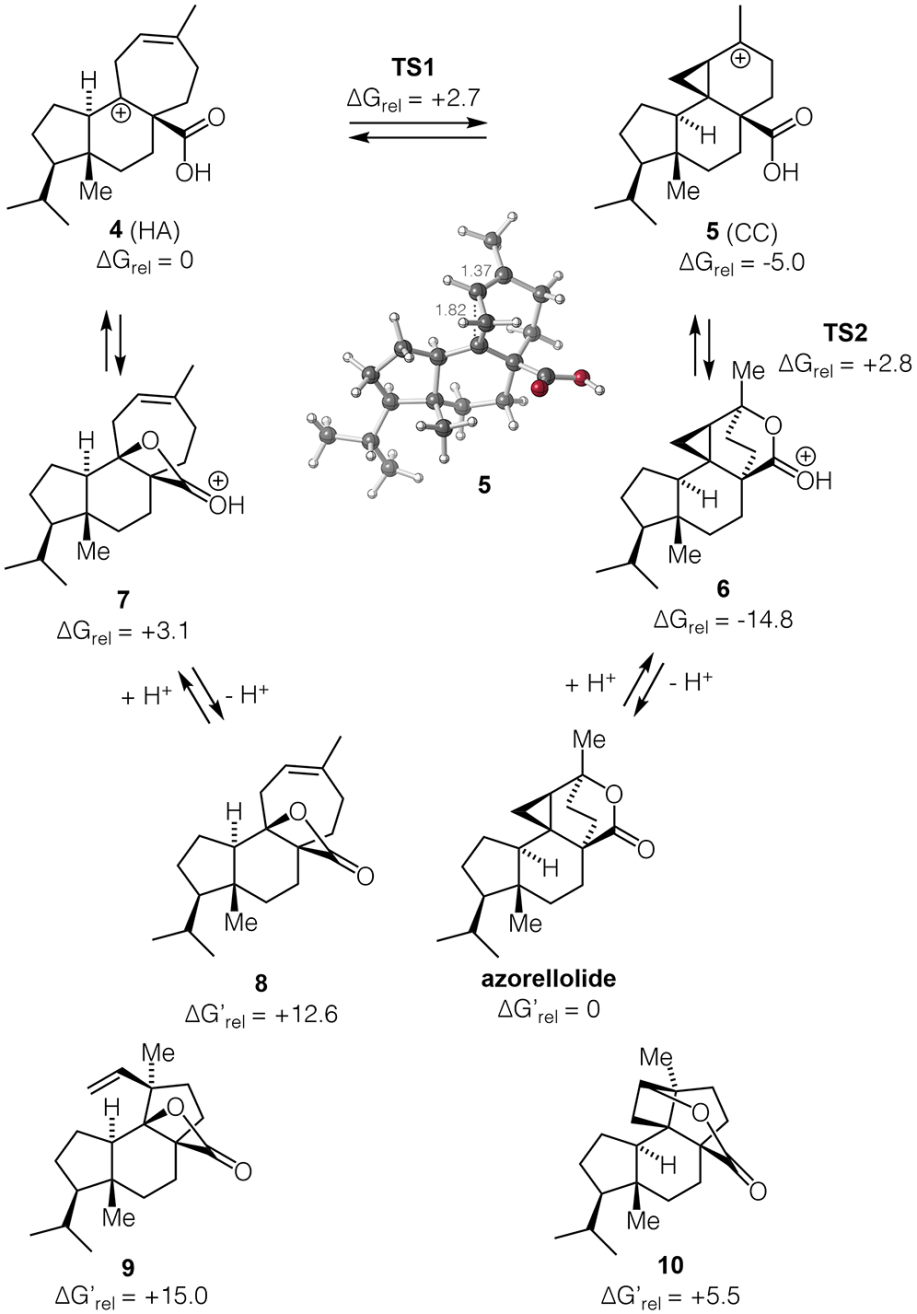
Implementation of the intramolecularization strategy. Shown are the relative energies of interconverting carbocations and deprotonated lactones, respectively. The energies of selected transition states (TS) are also indicated. The calculated structure of cation **5** with select bond lengths (in Å) is shown in in the center.

Based on these calculations we decided to aim for a suitable precursor of carbocation **4**, which we initially deemed to be the corresponding tertiary alcohol (compound **17**, Figure 4). Our synthesis of this precursor commenced with commercially available ergocalciferol (vitamin D_**2**_), which was oxidatively degraded and then reduced to afford the known secondary alcohol **11**.^30^ Oxidation with Bobbitt’s oxammonium salt to ketone **12** required careful optimization due a tendency of this *trans* hydrindanone to isomerize to its corresponding *cis* isomer^31−33^ (see SI). Regioselective deprotonation of **12** and reaction with Manders’ reagent^34^ gave a β-keto ester (not shown) that underwent diastereoselective Michael addition to methyl vinyl ketone (MVK) to afford **13**, which features a second quaternary stereocenter. Again, the reaction had to be carefully monitored to avoid isomerizing to the thermodynamically more stable *cis* hydrindanone. Selective olefination of the sterically less hindered methyl ketone could then be achieved with Nysted’s^35−37^ reagent and Ti(OiPr)_2_Cl_2_ to yield olefin **14**. Strongly basic conditions, such as methyl phosphonium bromide and KHMDS or BuLi, resulted in retro-Michael addition or epimerization. Allyl addition to the neopentylic ketone could be achieved under Barbier^38,39^ conditions using an organozinc reagent. The resultant diene **15** underwent smooth ring-closing metathesis to afford hydroxy ester **16**, which features a 5/6/7 mulinane skeleton. The single crystal X-ray structure of **16** is shown in Figure 4. Finally, this compound could be hydrolyzed under Krapcho conditions with excess LiCl and DMF at 120 °C to yield the desired hydroxy acid **17** together with recovered starting material.^40^

**Figure 4:**
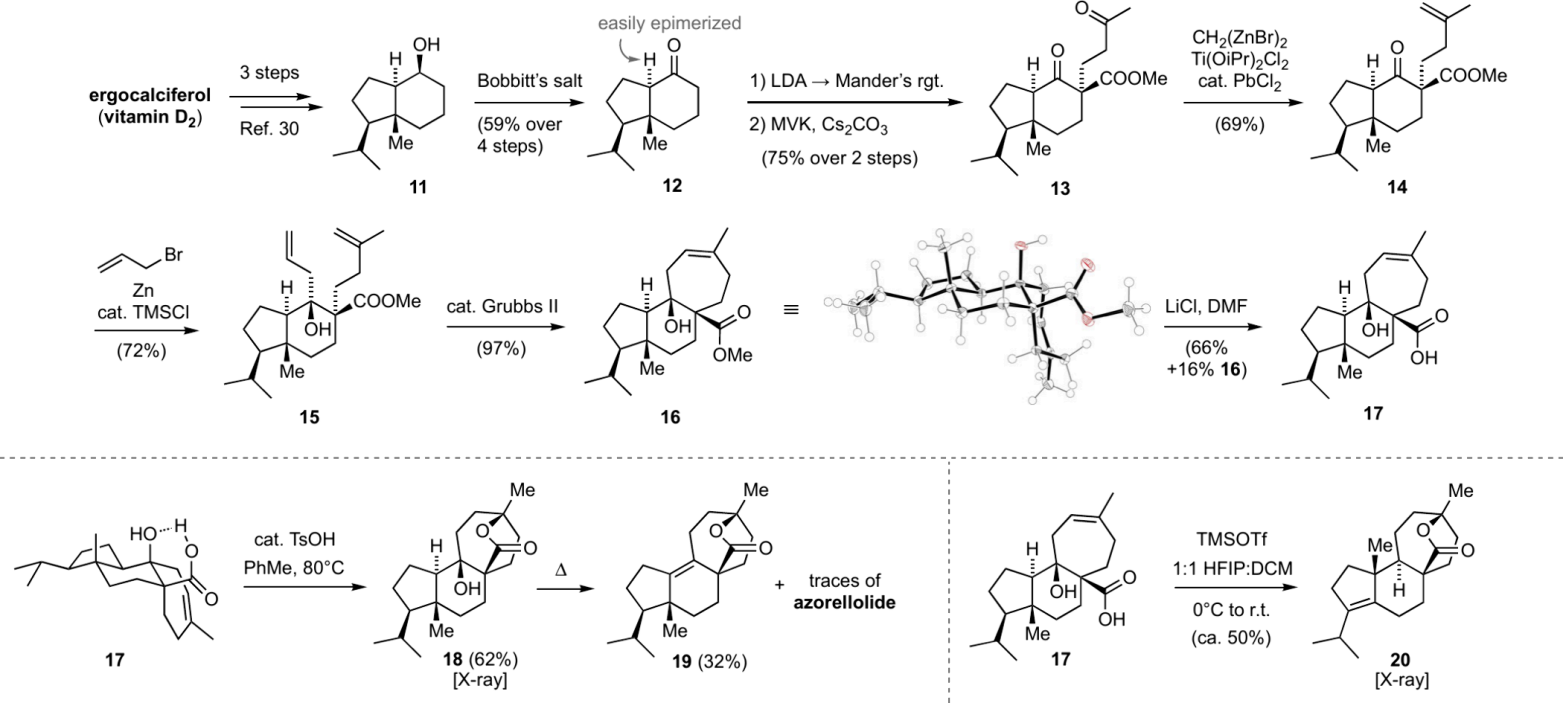
Preparation of cyclization precursors and unsuccessful cyclization attempts. The single crystal X-ray structure of **16** is shown.

With **17** in hand, we attempted its dehydration and transformation to azorellolide under a variety of acidic conditions (Figure 4, bottom). However, despite extensive efforts, we were unable to find or cleanly isolate azorellolide. For instance, treatment of **17** with tosic acid in toluene at elevated temperatures only led to addition of the carboxylate to the double bond, giving lactone **18**. Further heating led to elimination of the tertiary alcohol to yield tetrasubstituted alkene **19**. Azorellolide, easily identified in ^1^H NMR spectra of complex mixtures due the upfield shift of its cyclopropane protons, was only found in trace amounts under these conditions. Treatment of **17** with trimethylsilyl triflate (TMSOTf) in 1:1 HFIP: DCM led to addition of the carboxylic acid to the double bond and ionization of the tertiary alcohol, followed by hydride shift, methyl shift, and elimination, to yield lactone **20**, contaminated by double-bond isomers. The structure of **20** was confirmed by X-ray crystallography (see SI). This product was also observed upon exposure to SnCl_4_, TiCl_4_ or Sc(OTf)_3_.

We traced the reluctance of the tertiary alcohol in **17** to undergo protonation and ionization to its stabilization by an intramolecular hydrogen bond (Figure 4, bottom left). In addition, we reasoned that the formation of water, a small and comparatively strong base (p*K*_a_ of H_3_O^+^ = −1.74) during the ionization would complicate the reaction and lead to the formation of unwanted byproducts. Motivated by the computational results shown in Figure 3, we therefore decided to focus on isomerizations of strained lactones instead of eliminations. To this end, we synthesized β-lactone **8** from hydroxy acid **17** in high yield through activation with a mixed carboxylic-sulfonic anhydride (Figure 5). A single crystal X-ray structure of **8** confirmed our assignment.

**Figure 5:**
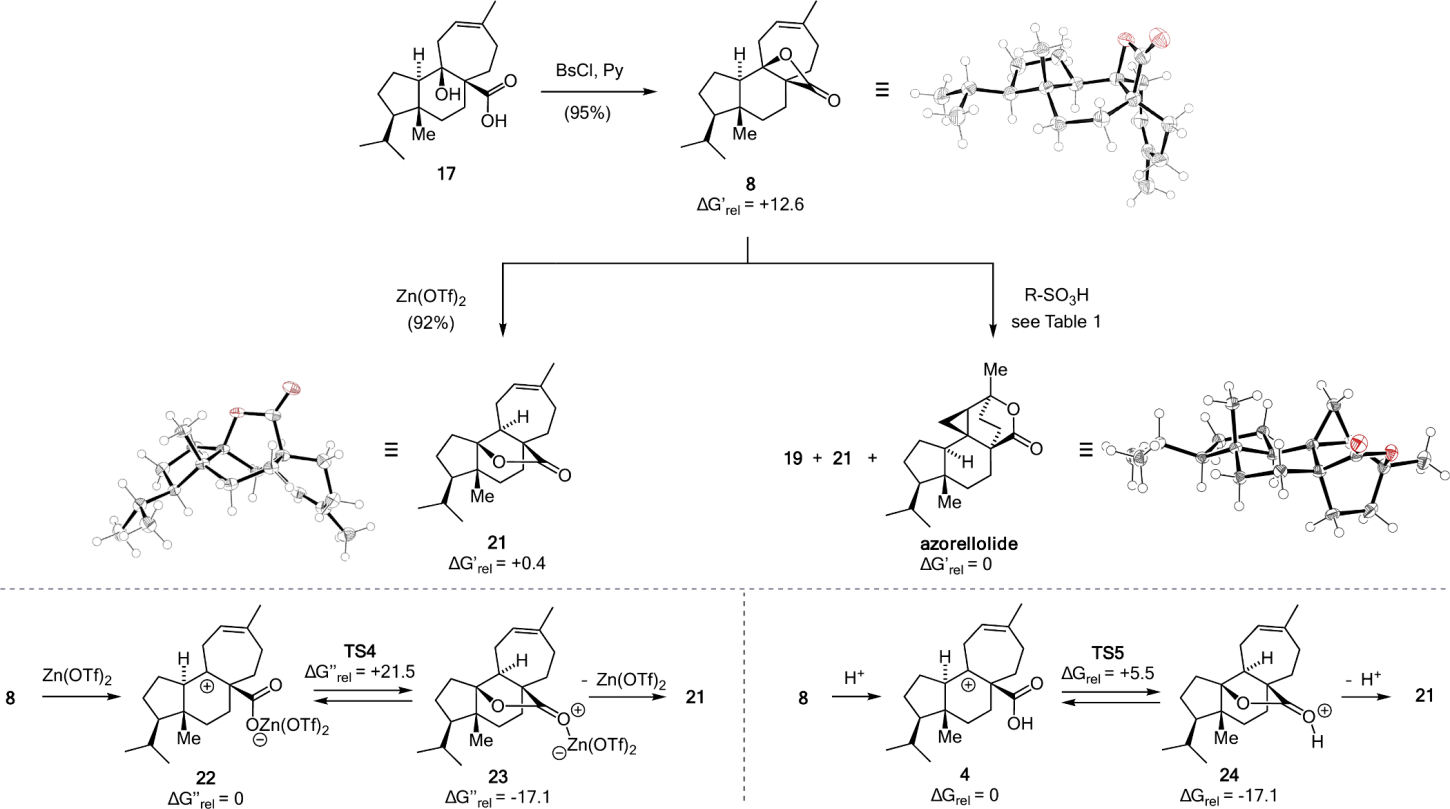
Biomimetic synthesis of azorellolide via β-lactone **8**, dyotropic rearrangement of **8**, and mechanistic interpretation. The single crystal X-ray structures if **8**, **21** and azorellolide are shown.

The isomerization of **8** to azorellolide was investigated under a variety of conditions. Exposure to Lewis acids, such as BF_3_·Et_2_O, Zn(OTf)_2_, Sc(OTf)_3_ or (C_6_F_5_)_3_B, led to clean isomerization to γ-lactone **21** through what can be seen as a type I dyotropic rearrangement.^41^ The single crystal X-ray structure of **21** is shown in Figure 5. Azorellolide could not be identified under these conditions.

By contrast, treatment of **8** with a range of strong protic acids gave azorellolide as the main product accompanied by varying amounts of δ-lactone **19** and γ-lactone **21** (Table 1, Figure 6). Initially, we found that exposure of 8 to five equivalents of 4-chlorobenzenesufonic acid delivered azorellolide in a modest 20% yield (entry 1). This promoted us to systematically explore the electronic range of sulfonic acids. Rendering the sulfonic acid more acidic (*e.g*., 2-nitro-4-trifluoromethyl-benzenesulfonic acid, entry 2) diminished the yield of azorellolide to 14%, leading to dominant formation of **19**. Benzenesulfonic acid (entry 3) aided in the formation of azorellolide (30% yield), but more electron-donating sulfonic acids such as 4-OMe-benzenesulfonic acid (entry 4) showed little to no effect on promoting the desired rearrangement compared to entry 3 (27% yield). Further optimization ultimately led us to 2-bromo-4- trifluoromethyl -benzenesulfonic acid in a toluene:MeCN mixture (5:1), which gave azorellolide in a 39% yield (entry 6). Finally, it was found that trifluoroethanol (TFE) and tosic anhydride (Ts_2_O) improved the isolated yield of azorellolide to a 48% yield (51% BRSM) (Figure 6 Bottom).

**Table 1:**
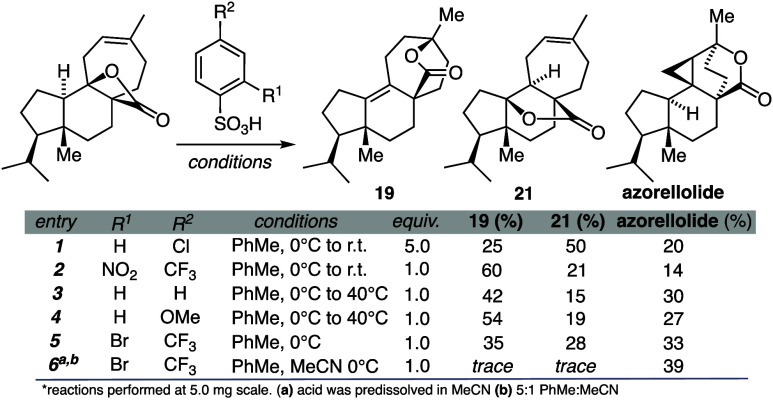
Selected Experiments for Aryl Sulfonic Acids

**Figure 6:**
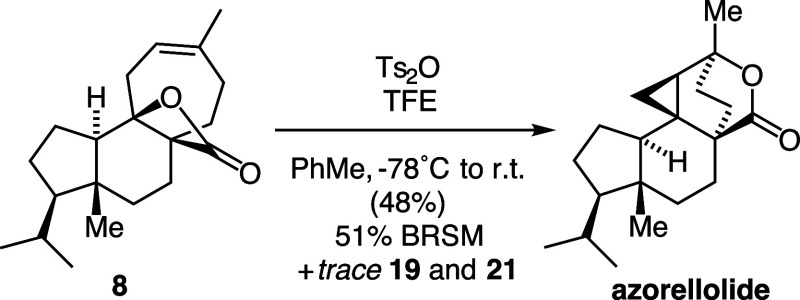
Optimized conditions for rearrangement.

Again, we resorted to quantum mechanical calculations to interpret these results (Figure 5, bottom). For both proton and Lewis acid promoted rearrangements, initial ring-opening to **22**/**4** is predicted to be followed by concerted 1,2-H shift/C–O bond formation, making these reactions stepwise dyotropic processes.^42−44^ Both stepwise and concerted but asynchronous dyotropic rearrangements have been proposed to occur in terpene biosynthesis.^45,46^ Not surprisingly, isomerization of β-lactone **8** (Δ*G*_rel_ = +12.6) to γ-lactone **21** (Δ*G*_rel_ = +0.4) is predicted to be thermodynamically strongly favored. However, while this conversion is predicted to be reversible under acidic conditions (Figure 5, bottom right), it is predicted not to be so with a Lewis acid (Figure 5, bottom left). Thus, only with protic acids is azorellolide, which is predicted to be slightly more stable than **21**, formed in significant amounts.

In sum, we have achieved a synthesis of the complex diterpene, azorellolide in eight steps form the known hydrindanone **12** (12 steps from commercially available ergocalciferol). It is the first synthesis of a member of the azorellane class and proceeds through an interception of a cyclopropylcarbinyl cation **5**, that presumably also occurs in the biosynthesis. In the laboratory, however, this cation is generated from a strained β-lactone (compound **8**). In the course of the isomerization of **8** to azorellolide, three stereocenters, one of which is quaternary, are formed in a single step. The results of quantum chemical computations were used throughout to shape synthetic strategy and interpret our results. According to our calculations and observations, the facile dyotropic rearrangement **8** → **21** could also occur in Nature, potentially making the γ-lactone **21** an anticipated natural product.^47^

## Data Availability

In addition to the Supporting Information (SI), computed structures are available through the ioChem-BD repository at doi:10.19061/iochem-bd-6-407.

## Supporting Information Available

Figures, Tables, experimental details and characterization data for all new compounds. (PDF)
