# Supplementary material for: Biomimetic Synthesis of Azorellolide via Cyclopropylcarbinyl Cation Chemistry
Source: J Am Chem Soc. 2024 Dec 18;147(1):78–83. doi: 10.1021/jacs.4c14664 (PMC11726563; doi:10.1021/jacs.4c14664)
Supplement: Supplementary file 1 — ja4c14664_si_001.pdf [file ja4c14664_si_001.pdf]

Supporting Information for

Biomimetic Synthesis of Azorellolide via Cyclopropylcarbinyl Cation Chemistry

Jordan Y. Artzy,[1] Dean J. Tantillo,[2*] and Dirk H. Trauner[1*]

[1]Department of Chemistry, University of Pennsylvania, Philadelphia, PA 19104, USA

[2]Department of Chemistry, University of California, Davis, CA 95616, USA

The PDF file includes:
Supporting Figures
General Information and Methods
Spectra
X-Ray Data

Supporting Information Figures

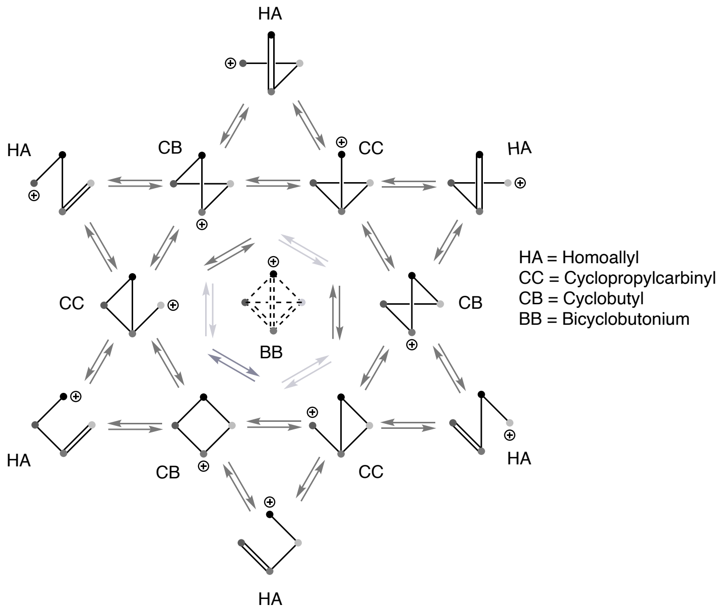

SI Figure 1: Enumeration of the CC-CB-HA manifold. If all four carbon atoms are distinguishable, three CC, three CB, and six HA cations are possible, which can interconvert. Stereoisomers are not taken into account. The BB cation is shown in the center.

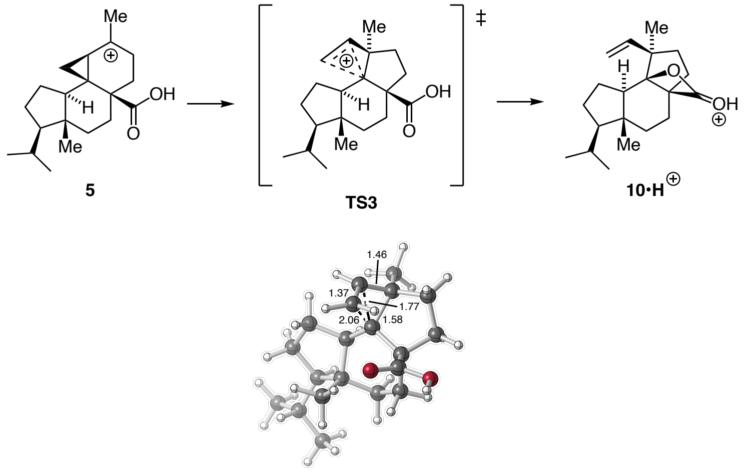

SI Figure 2: Transition state for the interconversion of **8** to the protonated form of **10**, resembling a BB cation.

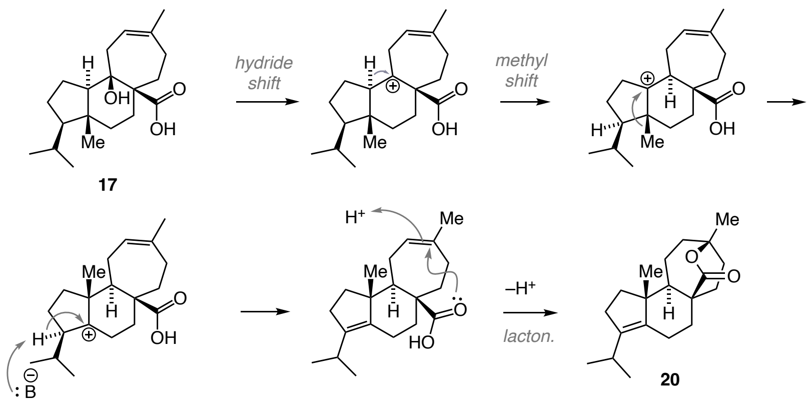

Proposed mechanism for the transformation of **17** to **20**.

General Information and Materials

Nuclear Magnetic Resonance (NMR) spectroscopy:

NMR spectra (^{1}H, ^{13}C{^{1}H}) were recorded on a Fourier transform NMR spectrometer at 298 K at 400, 500, or 600 MHz (for ^{1}H), 101, 126, or 151 MHz (for ^{13}C{^{1}H}). Chemical Shifts **(δ)** are reported in parts per million (ppm) relative to tetramethylsilane. The residual solvent signals of deuterated solvents $CDCl_3$ (7.260) and C_6D_6 (7.160) were used as internal references for ^{1}H NMR data and ($CDCl_3$: δ = 77.16 ppm, C_6D_6: δ = 128.06) for ^{13}C{^{1}H} spectra. Spin multiplicities are described as follows: s (singlet), d (doublet), t (triplet), q (quartet), p (pentet) in replacement of quintet, m (multiplet), br (broad) or a combination thereof. Structural analysis was conducted with ^{1}H- and ^{13}C-NMR spectra with the aid of additional 2D spectra (COSY, HMBC, HSQC, NOESY). Spectra analysis was conducted with the software MestReNova.

Mass spectrometry (MS):

Liquid chromatography mass spectrometry (LC-MS) analysis was performed on a Waters GCT Premier LC-MS or Bruker scimaX MRMS with electrospray ionization (ESI). Samples were taken up in acetonitrile for analysis, and signals were measured against an internal lock mass reference leucine enkephalin for ESI-LC-MS.

Infrared spectropscopy (IR):
IR spectra were recorded on a PerkinElmer FT-IR Spectrum Two spectrometer. For measurements, the neat substances were directly applied as a thin film on the ATR unit. The measured wavenumbers are reported to the nearest integer.

Optical rotation ($[\alpha]_D^T$)
Optical rotation values were measured on a Krüss P3000 Series polarimeter. The specific rotation values ($[\alpha]_D^T$) are reported in $\mathrm{deg \cdot dm^{-1} \cdot mL \cdot gm^{-1}}$ and calculated by the formula:

$$[\boldsymbol{\alpha}]_{\boldsymbol{D}}^{\boldsymbol{T}} = \frac{a \cdot 100}{l \cdot c}$$

T represents the ambient temperature (°C), D represents wavelength (in all cases, D is the sodium line: 589
nm), l represents the length of the cuvette (dm), c represents the concentration of the solution (g/100 mL).
α represents the measured rotation in degrees. The appropriate solvent and concentrations are reported in brackets. In all cases, T = 23°C.

Melting point (m_p):
Melting points were determined on a Stanford Research Systems DigiMelt melting point apparatus. The values are uncorrected.

Methods
Unless otherwise noted, all reactions were magnetically stirred under inert gas (Argon) atmosphere using standard Schlenk techniques. Glassware was evacuated and flame-dried with a butane torch. Drying over Na_2SO_4 implies stirring with an appropriate amount of

anhydrous salt for several minutes followed by filtration through a glass frit and rinsing of the filter cake with additional solvent used for extraction. Electric heating plates with heating mantles were used for reactions at elevated temperatures. For reactions below room temperature, the reaction vessel was cooled using a mixture of ice and water (0°C), or acetone and dry ice (–78°C). Stated reaction temperatures refer to the external bath temperature. Cannulas and syringes were used for the transfer of reagents and solvents which were flooded with inert gas (3x) before use. Purification by column chromatography was performed using manual air pressure, Geduran© Si60 silica gel (40–63 μm) from Merck KGaA. Silica gel F254 TLC plates from Merck KGaA were used for monitoring reactions, analyzing fractions of column chromatography, isolation for preparative plate TLC, and measuring R_f values. Plates were visualized using UV and stained with *p*-anisaldehyde followed by subsequent heating. Isolation of non–UV active compounds was performed by cutting a small slice of a preparative plate, staining with *p*-anisaldehyde, aligning, and approximating the compounds' location through alignment. Reaction yields refer to spectroscopically pure isolated amounts of compounds.

Chemicals

All chemicals were purchased from Sigma Aldrich, Fisher Scientific, TCI, Ambeed, Acros, Enamine, Aurora Chemicals, Acros Organics, Oakwood, and Resarch Products International (RPI). HPLC- and ACS grade solvents were purchased from Fisher Scientific and used as received without further purification. The expression "hexanes" refers to a mixture of hexane isomers with a boiling point between 40-80°C. Tetrahydrofuran (THF), dichloromethane (DCM), were dried and deoxygenated by passing through a Pure Process Technology (PPT) Solvent purification system. Diisopropylamine was distilled from CaH_2, and methyl vinyl ketone was distilled from calcium chloride and potassium carbonate. Other reaction solvents toluene (PhMe), dimethylformamide (DMF), hexafluoroisopropanol (HFIP), triethylamine (NEt_3), and pyridine (pyr.) were purchased from commercial sources (Acros Organics, Oakwood) and were stored under an inert gas atmosphere and over molecular sieves. All other reagents with a purity of >95% were purchased from commercial sources and used without further purification.

Computational Structures

Computed structures can be found through the ioChem-BD repository:
https://iochem-bd.bsc.es/browse/handle/100/328426

Staining solutions

p-anisaldehyde:
To 135 mL of absolute ethanol add 5 mL of conc. sulfuric acid and 1.5 mL of glacial acetic acid. Allow the solution to cool to room temperature. Add 3.7 mL of *p*-anisaldehyde. Stir the solution vigorously to ensure homogeneity.

Experimental Procedures

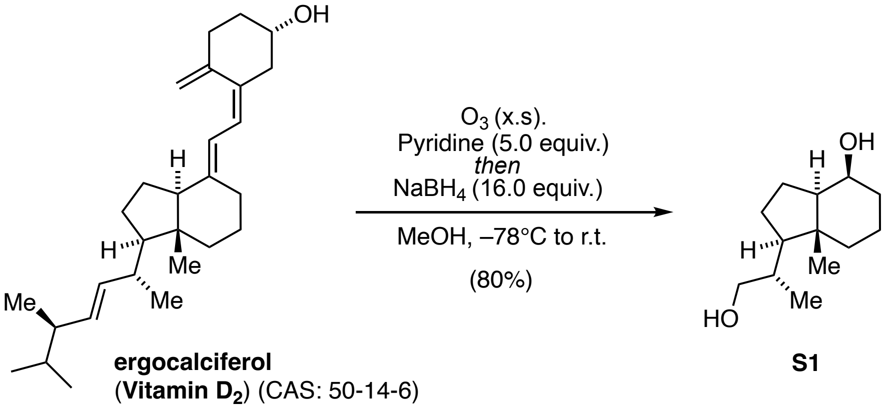

ergocalciferol (**Vitamin D$_2$**) (CAS: 50-14-6)

S1

Caution! Ozone is extremely toxic and can react explosively with certain oxidizable substances. It is essential that the reaction vessel remains dilute to avoid build-up of ozone. Ozone machines should be left in a well-ventilated fume hood when operating.

The following is a modification of a literature procedure.[1]
A flame–dried 2L round bottom flask equipped with a stir bar was charged with Vitamin D$_2$ (10.0 g, 25.2 mmol, 1.0 equiv.), pyridine (10.2 mL, 126 mmol, 5.0 equiv.), and dissolved in non–anhydrous MeOH (35.5 mM, ca. 800 mL). The reaction mixture was cooled to –78°C using a dry ice/acetone bath. A stream of ozone (60 L/h, 50 Hz) was passed through the solution at –78°C. After 2.5 h, a grey-blue color appeared, and the ozone flow was stopped. Argon was sparged into the solution until the solution was no longer blue (approximately 15 minutes). While stirring at –78°C, $NaBH_4$ (5.08 g, 134 mmol, 5.3 equiv.) was added *carefully* to avoid over-bubbling, and the solution was allowed to stir for 20 minutes. The reaction was then removed from the dry ice bath and $NaBH_4$ (5.08 g, 134 mmol, 5.3 equiv.) was added again to *carefully* avoid over-bubbling and left to stir for another 20 minutes. A final amount of $NaBH_4$ (5.09 g, 135 mmol, 5.4 equiv.) (total $NaBH_4$ added = 16.0 equiv.) was added *carefully* to avoid over-bubbling and left to stir at room temperature overnight. The reaction was quenched with a 1 M aqueous HCl (100 mL) solution and the crude mixture was then concentrated *in–vacuo,* and a dark yellow oil was obtained. The crude mixture was extracted with EtOAc (3 x 150 mL), washed with brine (150 mL), dried over anhydrous $NaSO_4$, and concentrated *in–vacuo.* The crude yellow oil was then purified with flash column chromatography (50% EtOAc/Hexanes) to afford **S1** as a crystalline solid in 80% yield (4.28 g, 20.1 mmol).

^{1}H NMR (500 MHz, $CDCl_3$) δ: 4.09 (q, J = 2.8 Hz, 1H), 3.64 (dd, J = 10.5, 3.3 Hz, 1H), 3.39 (dd, J = 10.5, 6.7 Hz, 1H), 1.99 (dt, J = 13.6, 3.2 Hz, 1H), 1.89 – 1.76 (m, 3H), 1.56 (tdd, J = 13.7, 8.9, 6.5 Hz, 2H), 1.52 – 1.41 (m, 3H), 1.39 – 1.24 (m, 2H), 1.18 (q, J = 10.2 Hz, 2H), 1.03 (d, J = 6.6 Hz, 3H), 0.96 (s, 3H).

R_f (EtOAc/Hexanes = 30%): 0.21 (*p*-anisaldehyde: dark blue)

Analytical Data is in accordance with the literature.[1]

^{1}H NMR (500 MHz, $CDCl_3$)

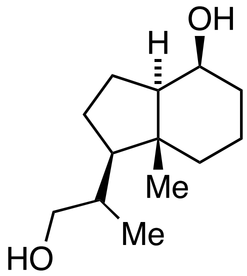

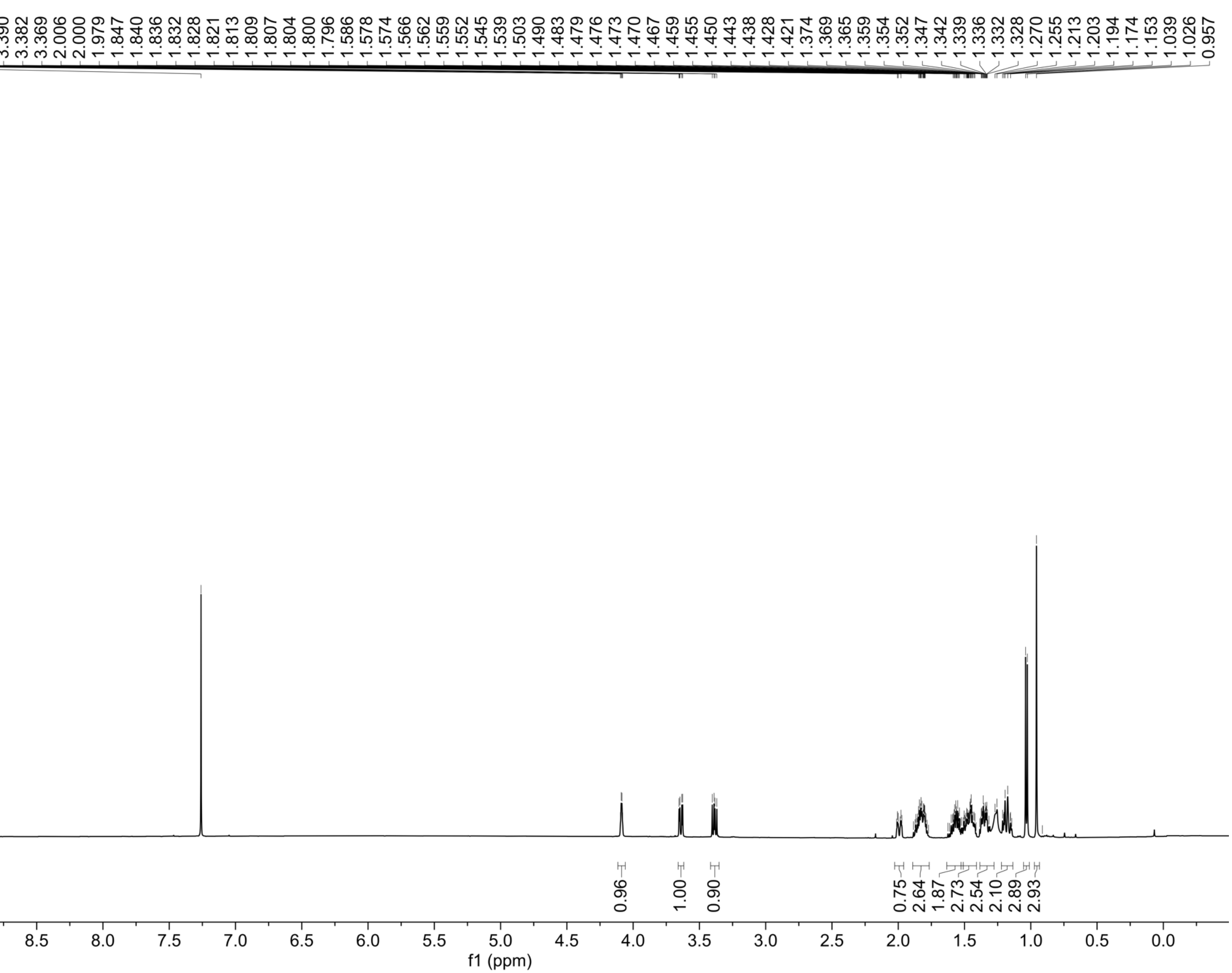

Ketone **12**

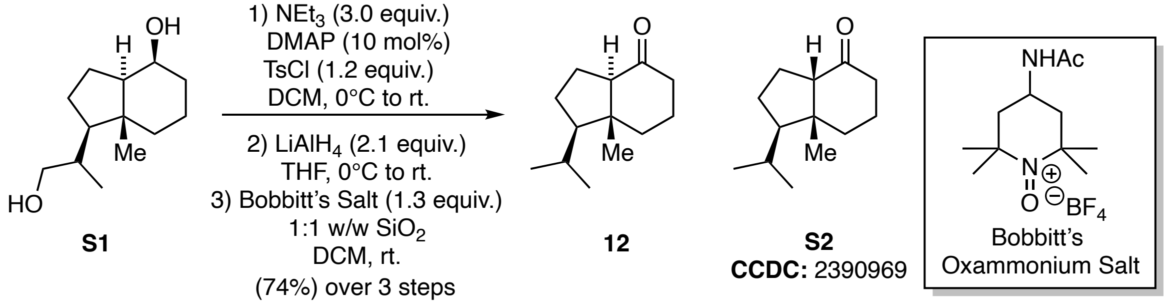

The following preparations of **12** and **S2** were adapted from the literature[2] with modification:

1. **Tosylation**

To a flame–dried 250 mL round bottom flask with stir bar was added **S1** (4.00 g, 18.8 mmol, 1.0 equiv.) and dissolved in 95 mL (0.20 M) of anhydrous DCM. The flask was cooled to 0°C, and to it was added triethylamine (7.81 mL, 56.5 mmol, 3.0 equiv.), DMAP (230 mg, 1.88 mmol, 0.10 equiv.) and left to stir for 5 minutes. *p*-toluenesulfonyl chloride (4.31 g, 22.6 mmol, 1.2 equiv.) was added and left to stir for 90 minutes. The color of the reaction mixture began to slowly turn yellow. The reaction can be checked for completion *via* a mini vial work-up (sat. $NaHCO_3$/EtOAc), LCMS analysis of starting material consumption, or by crude NMR analysis (preferred method). The reaction is diluted with DCM (50 mL). Saturated aqueous $NaHCO_3$ (60 mL) is added, the phases were separated. The organic extracts were washed with water, brine, and dried over Na_2SO_4, and concentrated *in–vacuo* to yield a yellow solid, carried forward without further purification.

2. **Tosylate Reductive Displacement**

The crude solid from the previous step was transferred to a flame-dried 250 mL 2-necked round bottom flask with stir bar and placed under argon in anhydrous THF (90 mL). The crude mixture was cooled to 0°C and stirred. Lithium aluminum hydride (750 mg, 19.8 mmol, 1.05 equiv.) was *slowly* added (**Caution: H2 Evolution!)** and allowed to stir for 2 minutes, and the bubbling subsided. Lithium aluminum hydride (750 mg, 19.8 mmol, 1.05 equiv.) was once again added and the reaction mixture was left to stir overnight (complete after 5 hours). Consumption of starting material was determined by TLC analysis and the reaction was quenched using the procedure developed by Fieser and Fieser[2]: the excess hydride was quenched by *dropwise* addition of water (0.750 mL), 15% aqueous NaOH solution (0.750 mL) and water (3 mL). After dilution with ethyl acetate (100 mL), the mixture was filtered through Celite® and concentrated *in–vacuo* to yield a light-yellow oil **11** (not shown in SI) that was carried forward without further purification.

3A. Oxidation Procedure A (No detected epimerization)

The crude material from the previous step was transferred to a 250 mL round–bottom-flask. Non–anhydrous DCM (150 mL) was added together with a stir bar. Then, a 1:1 w/w mixture of 4-(acetylamino)-2,2,6,6-tetramethyl-1-oxo-piperidinium tetrafluoroborate (Bobbitt's Salt) (8.79 g, 26.4 mmol, 1.4 equiv.) and silica gel (8.79 g) were added to the flask and left to stir vigorously

for 60 minutes. Upon consumption of starting material, the reaction was concentrated *in–vacuo* and was directly purified with flash column chromatography with 10% EtOAc/Hexanes to yield **12** as a yellow oil (2.70 g, 13.9 mmol) in a 74% yield over 3 steps.

3B. Oxidation Procedure: typical example that yielded Epimerized compound S2

The crude sample from reaction **2** (assumed to be 1.3 g, 6.6 mmol, 1 equiv.) in a 100 mL round–bottom-flask, with a stir-bar, was dissolved in anhydrous DCM (33 mL), to it was added Dess–Martin–periodinane (3.1 g, 7.3 mmol, 1.1 equiv.). The mixture was stirred at room temperature for 3 hours. Upon consumption of starting material, the reaction flask was concentrated *in–vacuo*, and the precipitated solids were filtered over Celite® and washed with Et_2O (2 x 15 mL). The organic layer was concentrated *in–vacuo* and purified by flash column chromatography (10% EtOAc/Hexanes), to yield **S2** (0.55 g, 2.8 mmol) in a 43% yield as pale-yellow crystals.

Desired Epimer 12:

^{1}H NMR (400 MHz, $CDCl_3$) δ: 2.44 (dd, *J* = 11.7, 7.5 Hz, 1H), 2.33 – 2.17 (m, 2H), 2.10 (ddd, *J* = 13.1, 4.8, 2.5 Hz, 1H), 2.05 – 1.96 (m, 1H), 1.96 – 1.83 (m, 2H), 1.79 – 1.66 (m, 1H), 1.59 (dd, *J* = 13.0, 5.0 Hz, 1H), 1.55 – 1.46 (m, 2H), 1.40 – 1.27 (m, 2H), 0.97 (d, *J* = 6.5 Hz, 3H), 0.88 (d, *J* = 6.6 Hz, 3H), 0.64 (s, 3H).

R_f (EtOAc/Hex = 10%): 0.30 (*p*-anisaldehyde: orange)

Undesired Epimer S2:

^{1}H NMR (400 MHz, $CDCl_3$) δ: 2.39 – 2.23 (m, 3H), 2.21 – 2.10 (m, 1H), 1.96 – 1.78 (m, 3H), 1.79 – 1.70 (m, 2H), 1.64 – 1.47 (m, 2H), 1.41 – 1.28 (m, 1H), 1.29 – 1.17 (m, 1H), 1.04 (s, 3H), 0.92 (d, *J* = 6.6 Hz, 3H), 0.85 overlapped with proton grease (1H) (d, *J* = 6.6 Hz, 3H).

Analytical data is in accordance with the literature.[1]

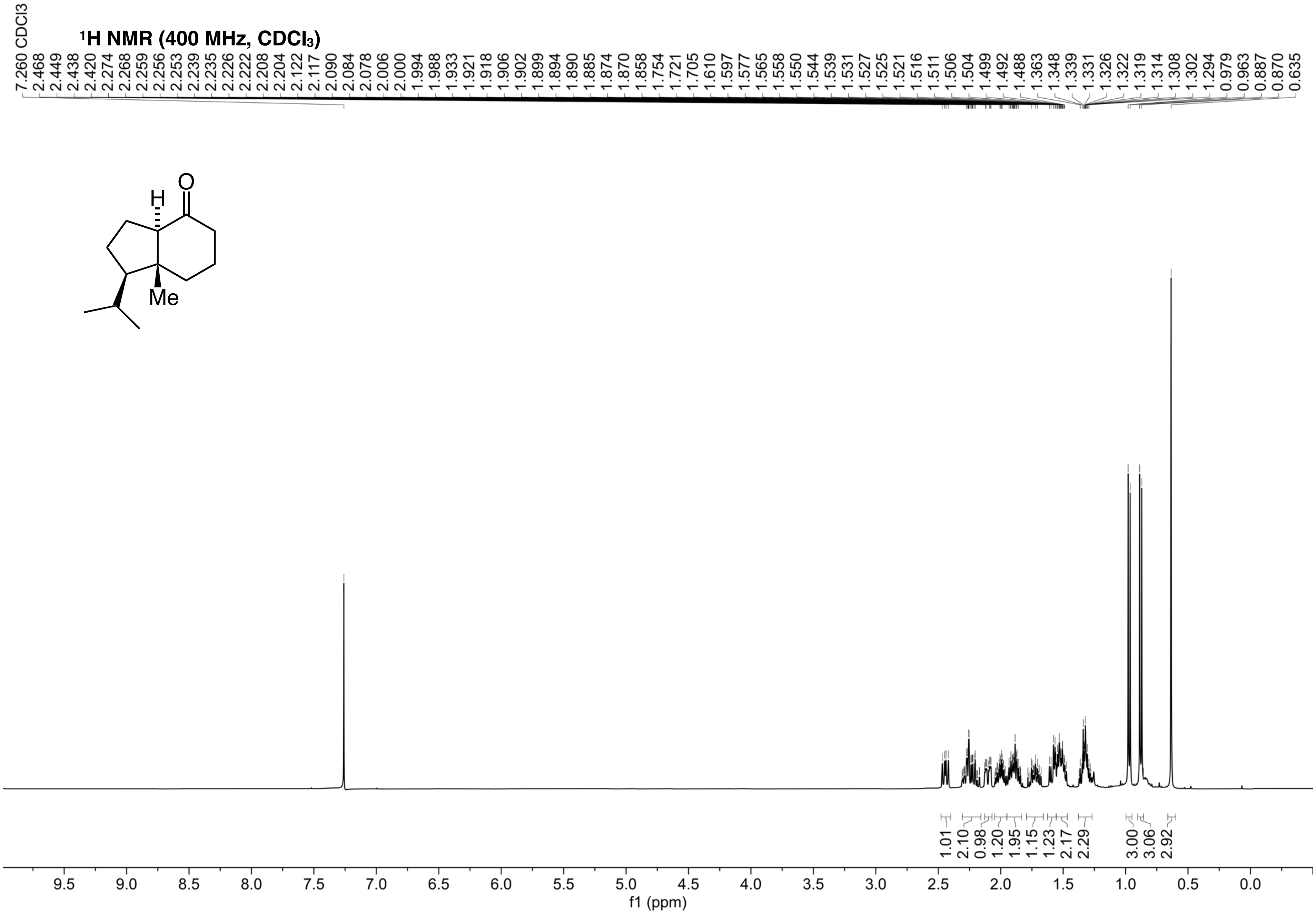

¹H NMR (400 MHz, CDCl₃)
7.260 CDCl3
2.468
2.449
2.438
2.420
2.274
2.268
2.259
2.256
2.253
2.239
2.235
2.226
2.222
2.208
2.204
2.122
2.117
2.090
2.084
2.078
2.006
2.000
1.994
1.988
1.933
1.921
1.918
1.906
1.902
1.899
1.894
1.890
1.885
1.874
1.870
1.858
1.754
1.721
1.705
1.610
1.597
1.577
1.565
1.558
1.550
1.544
1.539
1.531
1.527
1.525
1.521
1.516
1.511
1.506
1.504
1.499
1.492
1.488
1.363
1.348
1.339
1.331
1.326
1.322
1.319
1.314
1.308
1.302
1.294
0.979
0.963
0.887
0.870
0.635
H
O
Me
1.01
2.10
0.98
1.20
1.95
1.15
1.23
2.17
2.29
3.00
3.06
2.92
9.5
9.0
8.5
8.0
7.5
7.0
6.5
6.0
5.5
5.0
4.5
4.0
3.5
3.0
2.5
2.0
1.5
1.0
0.5
0.0
f1 (ppm)

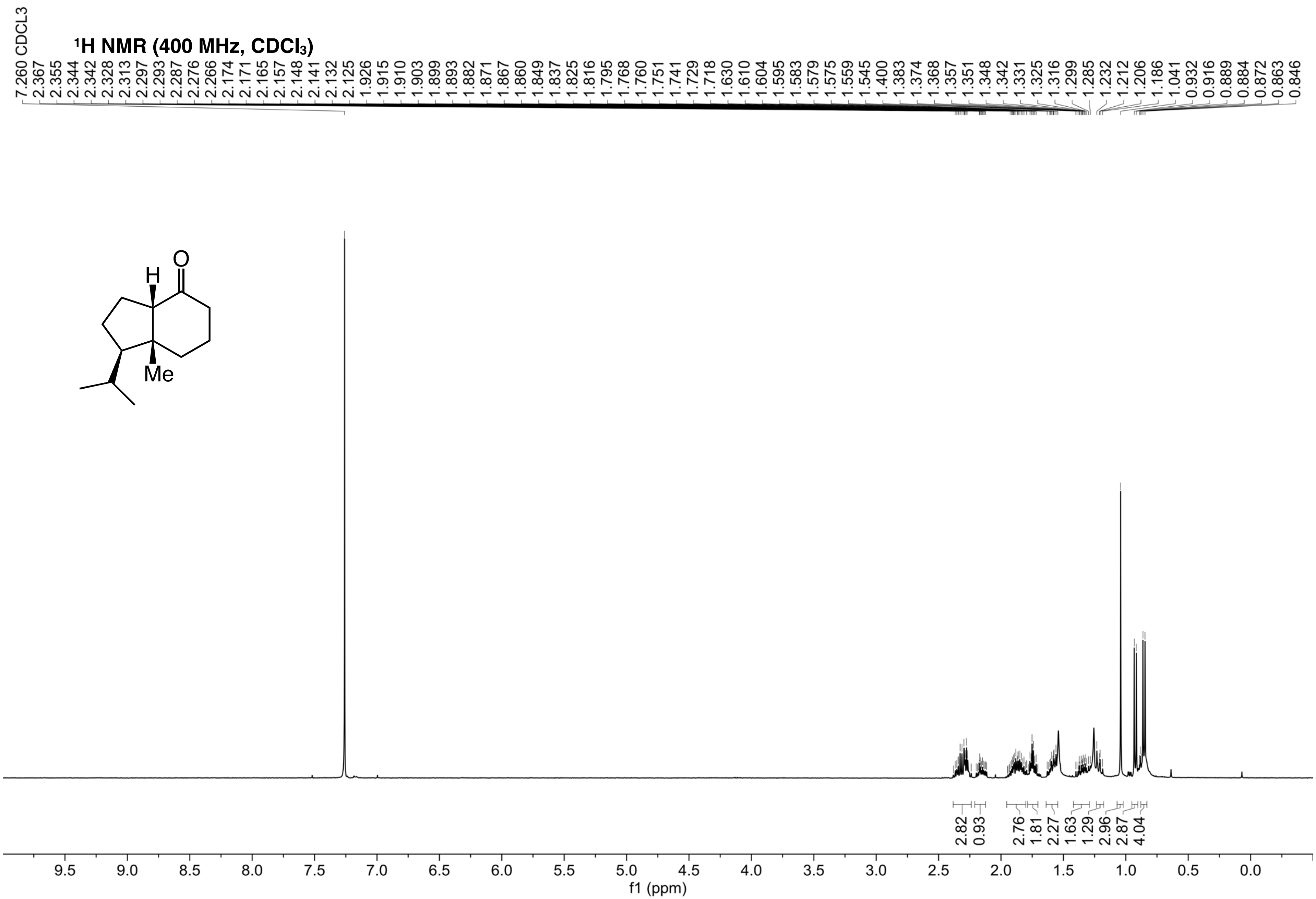

1H NMR (400 MHz, CDCl3)
7.260 CDCL3
2.367
2.355
2.344
2.342
2.328
2.313
2.297
2.293
2.287
2.276
2.266
2.174
2.171
2.165
2.157
2.148
2.141
2.132
2.125
1.926
1.915
1.910
1.903
1.899
1.893
1.882
1.871
1.867
1.860
1.849
1.837
1.825
1.816
1.795
1.768
1.760
1.751
1.741
1.729
1.718
1.630
1.610
1.604
1.595
1.583
1.579
1.575
1.559
1.545
1.400
1.383
1.374
1.368
1.357
1.351
1.348
1.342
1.331
1.325
1.316
1.299
1.285
1.232
1.212
1.206
1.186
1.041
0.932
0.916
0.889
0.884
0.872
0.863
0.846
O
H
Me
2.82
0.93
2.76
1.81
2.27
1.63
1.29
2.96
2.87
4.04
9.5
9.0
8.5
8.0
7.5
7.0
6.5
6.0
5.5
5.0
4.5
4.0
3.5
3.0
2.5
2.0
1.5
1.0
0.5
0.0
f1 (ppm)

β-keto-ester **S3**

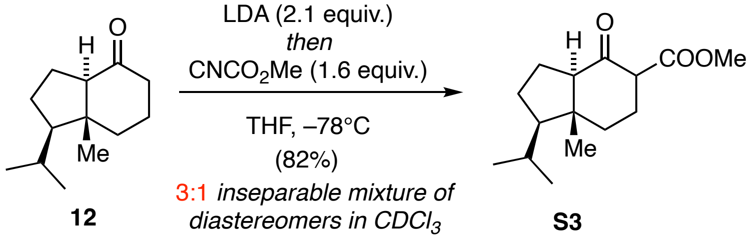

In a 250 mL flame-dried round bottom flask with stir bar was added a solution of LDA [prepared *in–situ* from freshly distilled diisopropylamine (1.07 mL, 7.55 mmol, 2.2 equiv.) and *n*-BuLi (2.5 M in hexane, 2.87 mL, 2.1 equiv.) in anhydrous THF (50 mL) at –78°C. Ketone **12** (664 mg, 3.42 mmol, 1 equiv.) in THF (10 mL) was added dropwise over 15 minutes (at –78°C). Stirring was continued for 60 minutes at –78°C. Subsequently, a solution of methyl cyanoformate (Mander's reagent) (434 *μ*L, 5.47 mmol, 1.6 equiv.) in THF (10 mL) was added dropwise over 15 minutes. The reaction was left to stir at –78°C for 1 hour, and determined to be complete by TLC analysis. At –78°C, saturated aqueous NH_4Cl (25 mL) was slowly added, followed by Et_2O (25 mL). The reaction slowly warmed to room temperature while stirring and the phases were separated. The organic phase was washed with brine (25 mL), dried with anhydrous Na_2SO_4 and concentrated *in–vacuo.* Flash column chromatography (10% EtOAc/Hexanes to 20% EtOAc/Hexanes) yielded **S3** as an inseparable 3:1 mixture of diastereomers with a combined yield of (710 mg, 3.42 mmol, 82% yield) as a pale-yellow oil.

^{1}H NMR (500 MHz, $CDCl_3$) δ: 3.64 (s, 3H), 3.61 (s, 1H), 3.26 (dd, *J* = 12.7, 6.9 Hz, 1H), 3.20 (d, *J* = 6.4 Hz, 0.3H), 2.52 (dd, *J* = 11.5, 7.3 Hz, 0.3H), 2.39 (dd, *J* = 11.7, 7.5 Hz, 1H), 2.33 – 2.19 (m, 1H), 2.11 – 2.01 (m, 2H), 1.99– 1.89 (m, 0.6H), 1.85 – 1.76 (m, 1H), 1.74 – 1.61 (m, 1.5H), 1.58 (td, *J* = 13.7, 4.8 Hz, 1H), 1.48 – 1.37 (qd, *J* = 10.2, 4.8 Hz, 3H), 1.30 – 1.19 (m, 3H), 0.87 (t, *J* = 6.6 Hz, 4H), 0.78 (t, *J* = 5.3 Hz, 4H), 0.59 (s, 3H), 0.56 (s, 1H).

^{13}C NMR (151 MHz, $CDCl_3$) δ: 205.6, 205.5, 170.6, 170.3, 61.8 (two peaks overlapped), 60.3, 58.6, 58.4, 56.8 55.6, 52.4, 51.8, 50.5, 50.25, 37.8, 36.3, 30.6, 27.7, 27.4, 27.3, 26.6 22.8, 22.4, 22.3, 19.0, 18.9, 12.5, 12.4.

IR (Diamond–ATR, neat): ν_{max} (cm^{-1}): 2955, 2871,1743, 1712, 1436, 1384, 1362, 1322, 1299.

HRMS (ESI): m/z $[M+H]^+$: calcd for $C_{15}H_{25}O_3^+$: 253.1804, found: 253.1816.

R_f (EtOAc/Hex = 20%): 0.55 and 0.53 (two spots). (UV, *p*-anisaldehyde: Brown)

***Note:** Optical Rotation was not taken for this compound due to the inseparable mixture of diastereomers.
***Note:** There is also believed to contain some of the enol form of the diastereomeric mixture.

^{1}H NMR (500 MHz, $CDCl_3$)

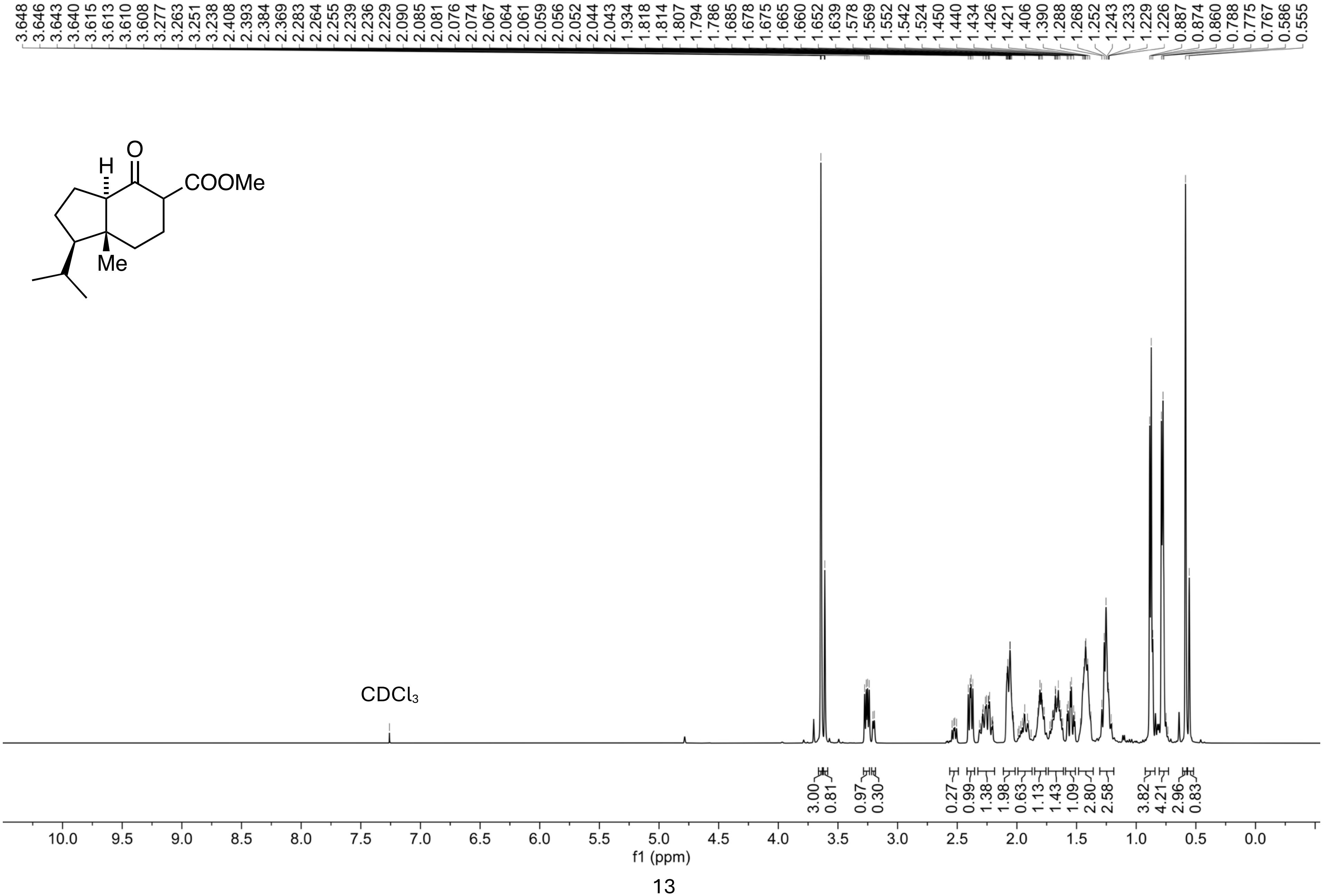

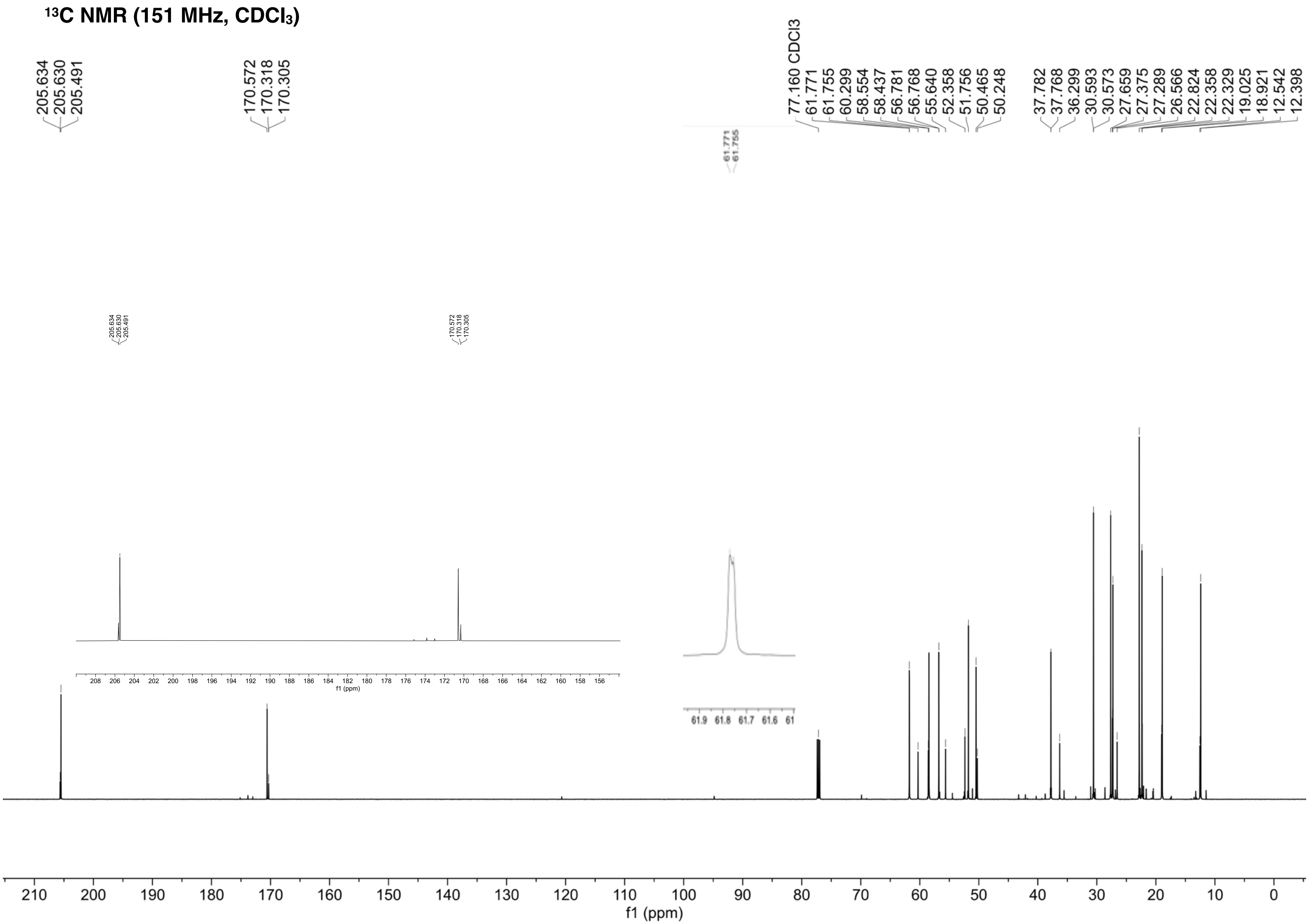

13C NMR (151 MHz, CDCl3)
205.634
205.630
205.491
170.572
170.318
170.305
77.160 CDCl3
61.771
61.755
60.299
58.554
58.437
56.781
56.768
55.640
52.358
51.756
50.465
50.248
37.782
37.768
36.299
30.593
30.573
27.659
27.375
27.289
26.566
22.824
22.358
22.329
19.025
18.921
12.542
12.398
f1 (ppm)

Tricarbonyl **13**

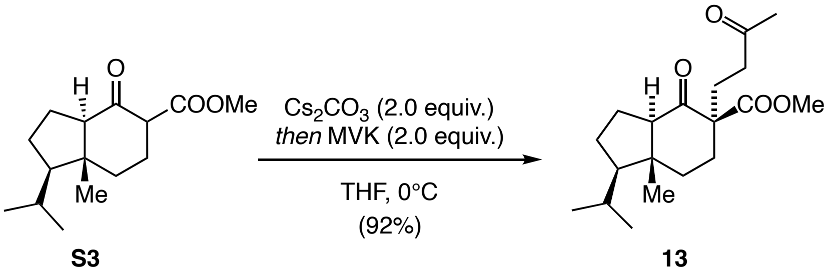

To a solution of **S3** (827 mg, 3.28 mmol, 1 equiv.) in anhydrous THF (50 mL) was added Cs_2CO_3 (2.14 g, 6.55 mmol, 2.0 equiv.) at 0°C. The mixture was stirred at the same temperature for 20 minutes and freshly distilled methyl vinyl ketone (546 µL, 6.55 mmol, 2.0 equiv.) in THF (5 mL) was added over 2 minutes. The reaction was then warmed to room temperature and left to stir for 2 hours to completion. The reaction was quenched with saturated aqueous NH_4Cl solution (50 mL) and extracted with EtOAc (3 × 50 mL). The combined organic layers were washed with brine (60 mL), dried over anhydrous Na_2SO_4, filtered and concentrated *in–vacuo.* Purification of the residue by flash column chromatography (30% EtOAc/Hexanes) yielded diketone **13** (972 mg**,** 3.01 mmol) in a 92% yield as a pale-yellow oil, that upon storage at –20°C overnight crystallized into a white solid.

$[\alpha]_D^{23}$ **=** –122.5° (c = 0.25, $CHCl_3$).

^{1}H NMR (500 MHz, $CDCl_3$) δ: 3.72 (s, 3H), 2.72 (ddd, *J* = 18.6, 9.9, 5.1 Hz, 1H), 2.61 – 2.48 (m, 2H), 2.25 – 2.11 (m, 2H), 2.09 (s, 3H), 2.09 – 2.01 (m, 1H), 1.94 – 1.62 (m, 4H), 1.53 – 1.44 (m, 2H), 1.36 – 1.27 (m, 2H), 0.95 (d, *J* = 6.5 Hz, 3H), 0.85 (d, *J* = 6.6 Hz, 3H), 0.72 (s, 3H).

^{13}C NMR (151 MHz, $CDCl_3$) δ: 209.8, 208.0, 173.6, 60.9, 58.5, 58.5, 52.3, 49.8, 39.3, 35.7, 33.1, 30.8, 30.1, 28.0, 27.7, 23.0, 22.6, 19.3, 12.6.

IR (Diamond–ATR, neat): v_{max} (cm^{-1}): 2953, 2887, 1737, 1704, 1469,1414,1374, 1302, 1252,1232,1216,1186,1111,1065, 994, 855, 583, 555.

HRMS (ESI): m/z $[M+H]^+$: calcd for $C_{19}H_{31}O_4^+$: 323.2222, found: 323.2242.

R_f (EtOAc/Hex = 30%): 0.33 (*p*-anisaldehyde: red)

m$_p$: 54.3 – 56.2°C

***Note**: It is essential to stir the reaction mixture vigorously and to quench after two hours as longer times lead to the formation of an inseparable 1:1 mixture of the *cis–(not shown)* and the desired *trans*–hydrindane **13**.

¹H NMR (500 MHz, $CDCl_3$)

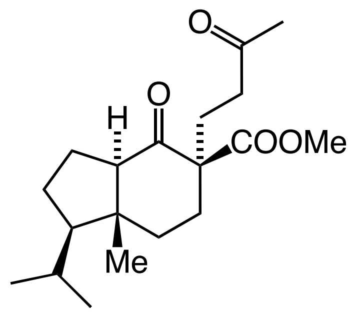

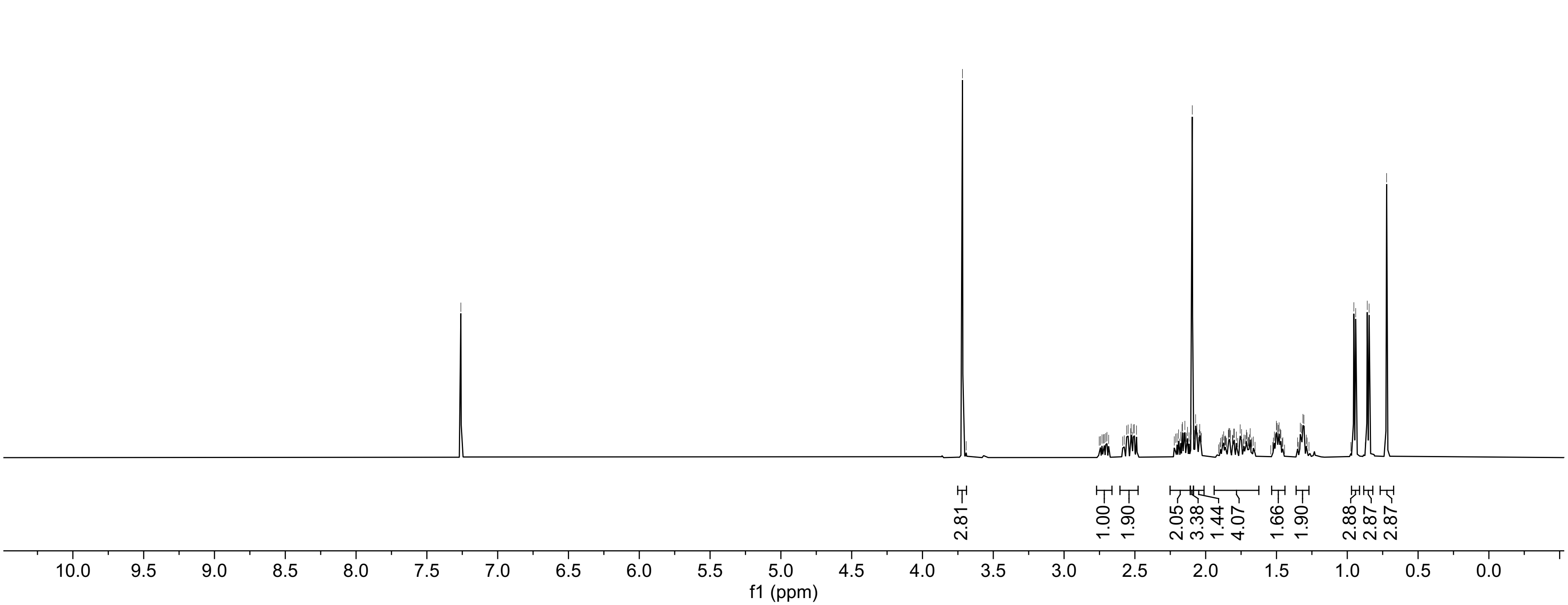

16

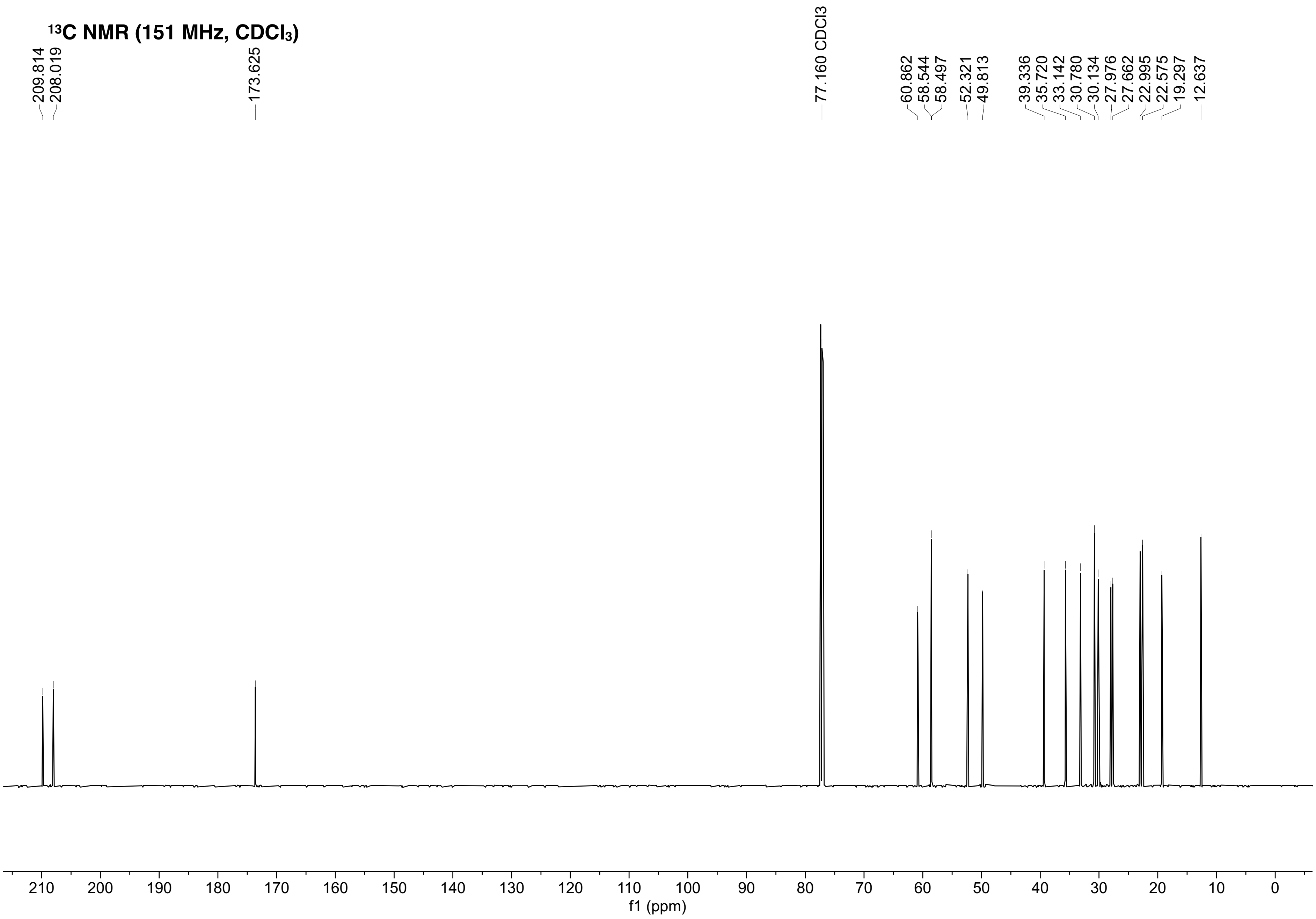

^{13}C NMR (151 MHz, CDCl$_3$)
209.814
208.019
173.625
77.160 CDCl3
60.862
58.544
58.497
52.321
49.813
39.336
35.720
33.142
30.780
30.134
27.976
27.662
22.995
22.575
19.297
12.637
210 200 190 180 170 160 150 140 130 120 110 100 90 80 70 60 50 40 30 20 10 0
f1 (ppm)

Olefin **14**

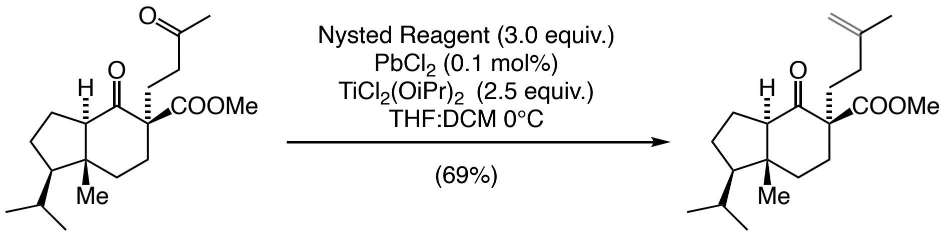

The following preparation of the Nysted Reagent and $TiCl_2(OiPr)_2$ were adapted from the literature[3] with modification:

$TiCl_2(OiPr)_2$[3]

In a 40 mL flame–dried septa-capped vial under argon atmosphere, equipped with a stir bar, $TiCl_4$ (15 mL, 1 M in DCM) was added. Subsequently, $Ti(OiPr)_4$ (4.26 mL, 15.0 mmol, 1 equiv.) was added dropwise. The mixture was stirred for 30 min at room temperature. The vial containing the brown solution was covered sealed with electrical tape and stored in a –20˚C freezer.

*__Note__: $TiCl_2(OiPr)_2$ crystallizes at –20°C and should be warmed to ambient temperature before use.

Nysted Reagent[3] ($CH_2(ZnBr)_2$ in THF)**:**

In a 40 mL flame–dried septa-capped vial with a stir bar under argon atmosphere, Zn dust 325 mesh grade (3.27 g, 50.0 mmol, 2.5 equiv.) and $PbCl_2$ (5.6 mg, 20.0 μmol, 0.1 mol%) were suspended in 4 mL of dry THF. TMSCl (not distilled) (130 μL, 1.00 mmol, 2 mol% per mol of Zn) was added followed by addition of dibromomethane (140 μL, 2.00 mmol, 10 mol%). The Zn was activated by sonication in an ultrasonic bath at 25˚C for 15 min. Then 16 mL of dry THF was added, followed by the dropwise addition of dibromomethane (1.40 mL, 20.0 mmol, 1 equiv.). Sonication of this mixture at 25˚C for 1 h provided the active reagent as a grey/white suspension, which was directly used.

The following procedure is a slightly modified version of a reported procedure.[3]

To a flame-dried 250 mL round bottom flask was added **13** (1.30 g, 4.03 mmol, 1 equiv.), dissolved in 81 mL of anhydrous THF, and cooled to 0°C. A 1.0 M $Ti(Oi\text{-}Pr)_2Cl_2$ solution in DCM (10.1 mL, 10.1 mmol, 2.5 equiv.; prepared as described) was added, followed by 0.5 M mixture of $CH_2(ZnBr)_2$ in THF (24.2 mL, 12.1 mmol, 3 equiv. prepared as described above) added dropwise over 10 minutes. The solution turned a dark brown color, and the reaction was left to stir for 20 minutes until the starting material was consumed. Upon consumption, the reaction was diluted with 200 mL of Et_2O and filtered through a pad of Celite® along with 100 mL of Et_2O washings of the solid residue. The black solution was then carefully quenched with ice cold aqueous 1 M HCl (100 mL) and then transferred to a separatory funnel. The organic layer was

washed one more time with aqueous 1 M HCl, and the organic phase became clear. The solution was extracted with Et_2O (3 x 50 mL) and the combined organic layers were washed with brine, dried over anhydrous Na_2SO_4 and concentrated *in–vacuo.* Purification of the residue by flash column chromatography (10% Et_2O/Hexanes) yielded *exo*–olefin **14** (890 mg, 2.78 mmol) in a 69% yield as a yellow oil.

$[\alpha]_D^{23}$ **=** –90.5° (c = 0.1, $CHCl_3$).

^{1}H NMR (600 MHz, C_6D_6) δ: 4.86 (s, 1H), 4.83 (s, 1H), 3.47 (s, 3H), 2.60 (td, *J* = 14.1, 5.0 Hz, 1H), 2.48 (ddd, *J* = 15.4, 11.7, 4.4 Hz, 1H), 2.22 (dd, *J* = 11.5, 7.4 Hz, 1H), 2.11 – 1.96 (m, 1H), 1.93 – 1.79 (m, 2H), 1.74 – 1.63 (m, 5H), 1.50 (ddd, *J* = 13.2, 6.7, 3.6 Hz, 1H), 1.40 (ddd, *J* = 13.5, 7.2, 3.1 Hz, 1H), 1.31 (td, *J* = 13.6, 4.7 Hz, 1H), 1.21 (dp, *J* = 9.9, 6.6 Hz, 1H), 1.03 – 0.93 (m, 1H), 0.80 (dd, *J* = 10.9, 8.0 Hz, 4H), 0.75 (d, *J* = 6.6 Hz, 3H), 0.62 (s, 3H).

^{13}C NMR (151 MHz, C_6D_6) δ: 207.8, 173.2, 145.8, 110.2, 61.7, 58.5, 58.3, 51.8, 49.6, 36.0, 33.50, 32.7, 32.2, 30.8, 27.8, 23.0, 22.8, 22.6, 19.6, 12.6.

IR (Diamond–ATR, neat): v_{max} (cm^{-1}): 2954, 2871, 1737, 1708, 1649**,** 1452, 1384, 1253,1240,1167, 1112, 1092, 997.

HRMS (ESI): m/z $[M+H]^+$: calcd for $C_{20}H_{32}O_3^+$: 321.2434, found: 321.2430.

R_f (EtOAc/Hex = 5%): 0.38 (*p*-anisaldehyde: dark blue)

^{1}H NMR (600 MHz, C_6D_6)

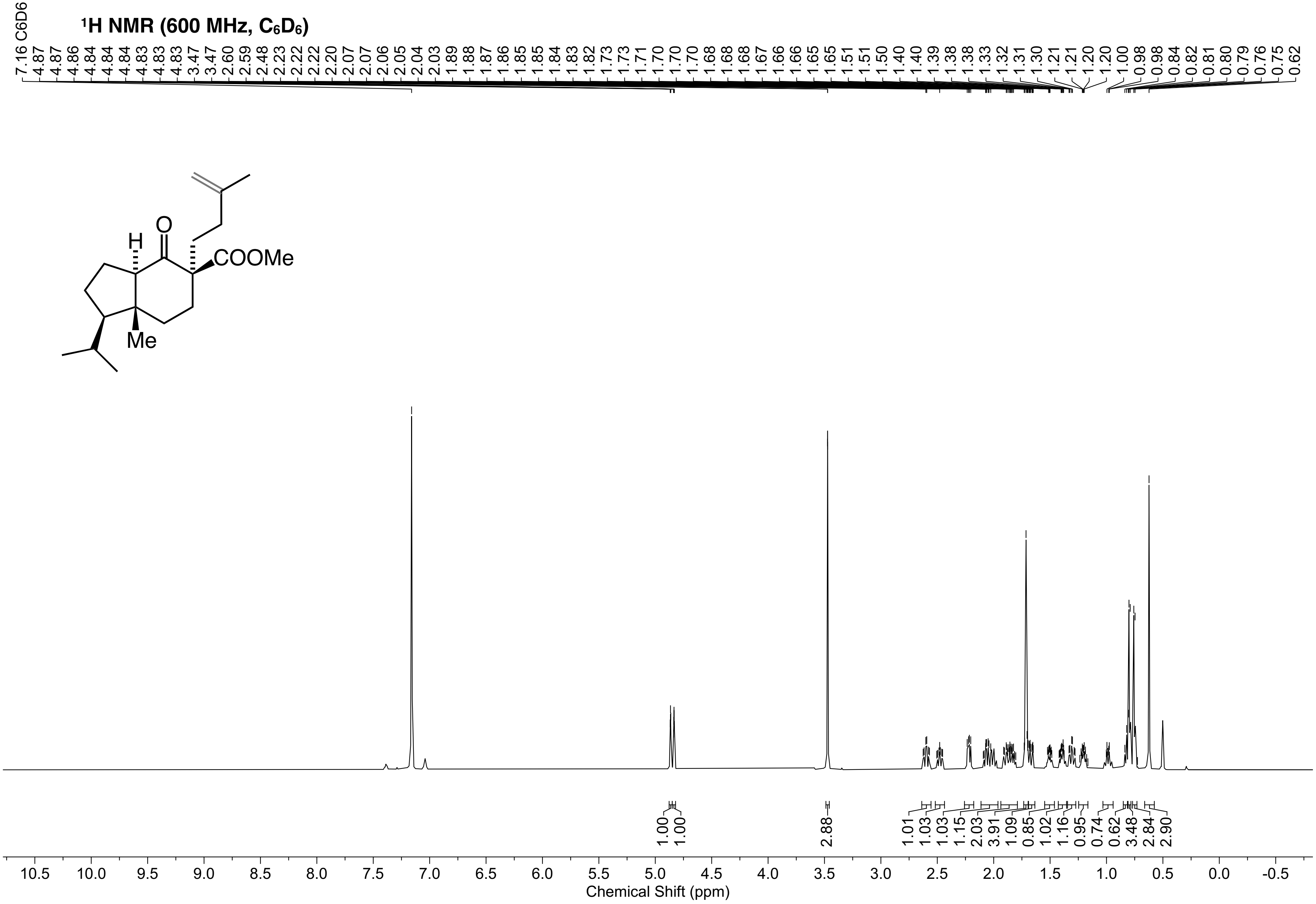

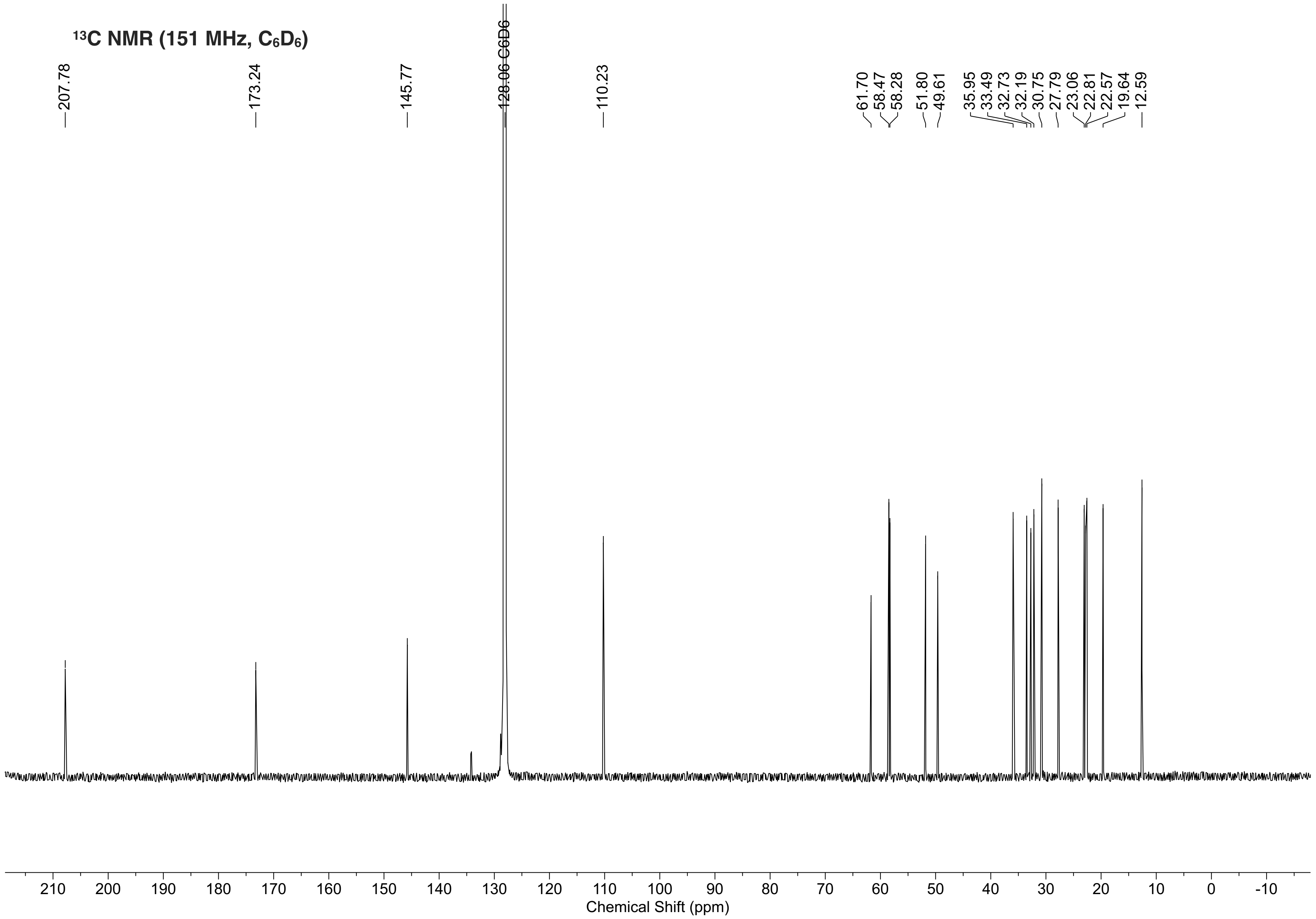

^{13}C NMR (151 MHz, C_6D_6)
207.78
173.24
145.77
128.06 C6D6
110.23
61.70
58.47
58.28
51.80
49.61
35.95
33.49
32.73
32.19
30.75
27.79
23.06
22.81
22.57
19.64
12.59
210 200 190 180 170 160 150 140 130 120 110 100 90 80 70 60 50 40 30 20 10 0 -10
Chemical Shift (ppm)

Diene **15**

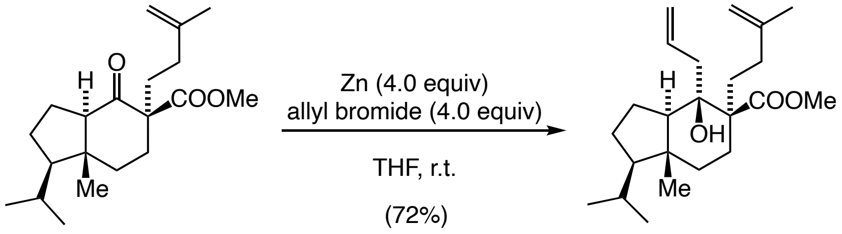

14 **15**

The following is a modification from the following report.[4]

To a flame–dried 40 mL vial, equipped with a septum and a stir bar, 325 mesh grade Zn (81.6 mg, 1.26 mmol, 4.0 equiv.) and allyl bromide (108 µL, 1.25 mmol 4.0 equiv.) in THF (5 mL) were added. The solution was left to stir for 5 minutes, and **14** (100 mg, 312 µmol, 1 equiv.) was added as a solution in THF (3 mL) with (2 x 1 mL) additional backwashes. The mixture was stirred and TMSCl (3.17 µL, 25.0 µmol, 0.08 equiv.) was added; then the reaction was left to stir for 30 minutes as a gray–opaque solution. The reaction was determined to be complete upon formation of light gray–translucent solution, and confirmed by TLC analysis of starting material consumption (see below). The reaction was immediately quenched with saturated aqueous $NaHCO_3$ (10 mL), and the aqueous layer was extracted (3 x 10 mL) with EtOAc. The combined organic layer was washed with brine, dried over anhydrous Na_2SO_4 and concentrated *in–vacuo.* Purification *via* flash column chromatography (10% Et_2O/Hexanes) afforded (82.2 mg, 0.224 mmol) of diene **15** in 72% yield as a clear oil.

$[\alpha]_D^{23}$ = –15.7° (c = 0.2, $CHCl_3$).

^{1}H NMR (500 MHz, C_6D_6) δ: 5.86 (dddd, *J* = 16.8, 10.1, 8.3, 6.5 Hz, 1H), 5.10 (ddt, *J* = 17.2, 2.6, 1.4 Hz, 1H), 5.00 (ddt, *J* = 10.1, 2.1, 1.0 Hz, 1H), 4.87 (d, *J* = 17.5 Hz, 2H), 3.31 (s, 3H), 3.07 – 3.04 (m, 1H), 2.79 (dd, *J* = 14.1, 8.3 Hz, 1H), 2.62 (dt, *J* = 11.2, 2.9 Hz, 1H), 2.29 (dd, *J* = 14.0, 6.5 Hz, 1H), 2.16 (td, *J* = 8.8, 2.6 Hz, 1H), 2.08 (td, *J* = 12.8, 4.8 Hz, 1H), 1.97 (td, *J* = 12.6, 3.5 Hz, 1H), 1.78 (td, *J* = 5.5, 2.6 Hz, 1H), 1.74 (s, 3H), 1.71 – 1.66 (m, 3H), 1.58 – 1.49 (m, 1H), 1.48 – 1.40 (m, 1H), 1.41 – 1.34 (m, 1H), 1.24 – 1.14 (m, 2H), 1.12 (s, 3H), 0.90 (d, *J* = 6.5 Hz, 3H), 0.86 (d, *J* = 6.6 Hz, 3H), 0.75 (d, *J* = 9.6 Hz, 1H).

^{13}C NMR (151 MHz, C_6D_6) δ: 177.2, 146.1, 135.3, 118.6, 110.4, 77.0, 58.9, 54.7, 51.3, 43.2, 42.3, 35.9, 33.8, 30.8, 29.1, 28.1, 25.0, 23.2, 22.7, 22.5, 21.6, 13.9.

IR (Diamond–ATR, neat): v_{max} (cm^{-1}): 3508, 2950, 1715, 1648, 1451, 1382, 1239, 1171, 1004, 885.

HRMS (ESI): m/z [M+Na]$^+$: calcd for $C_{23}H_{38}O_3Na^+$: 385.2724, found: 385.2713.

R_f (EtOAc/Hex = 10%): 0.38 (*p*-anisaldehyde: deep violet)

***Note:** The R_f of **14** and **15** are almost the same, thus, the reaction can be determined to be completed *via* LC–MS analysis by monitoring consumption of starting material. Fresh *p*-anisaldehyde stain will stain the compound a deep violet relative to a dark blue from the ketone. Slight separation can also be seen on TLC in 10% Et_2O/Hexanes with a co–spot of the starting material and reaction mix. It is recommended to use a glass-backed plate to observe starting material consumption on the back of the plate.

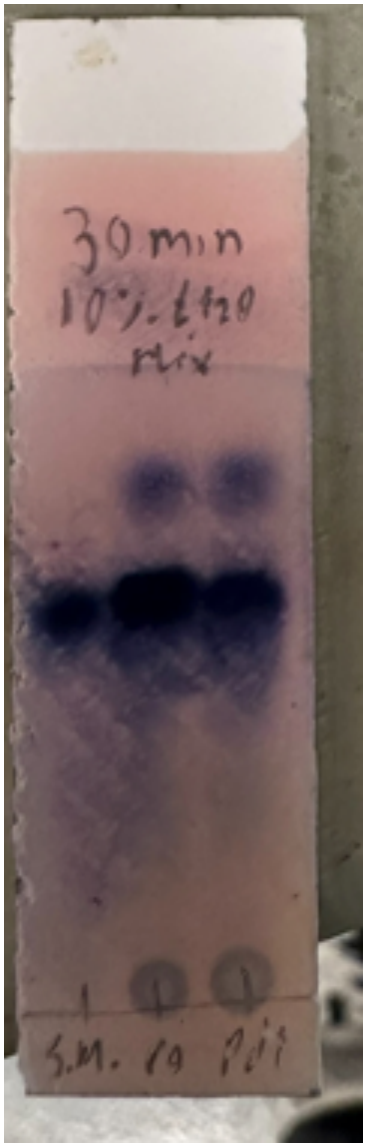

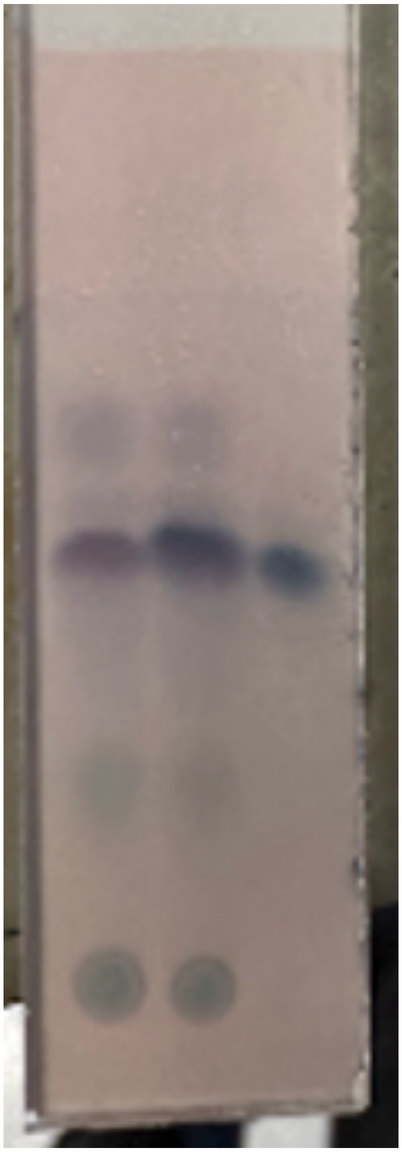

Front of TLC plate after 30 minutes. Difficult to see consumption,
glass back of TLC plate shows compound with similar R_f however stains a different color with p-anisaldehyde stain.

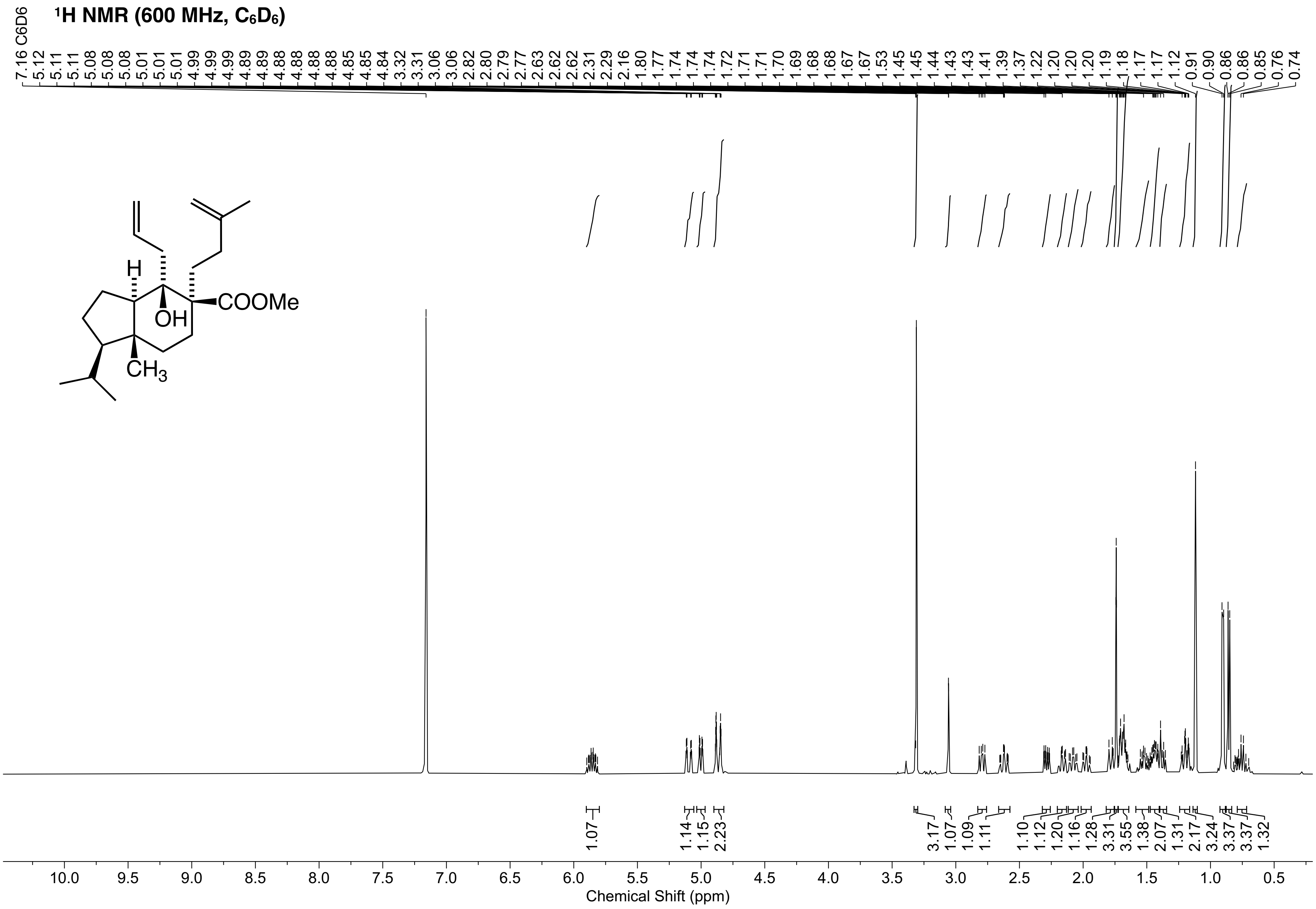
1H NMR (600 MHz, C6D6)
7.16 C6D6
5.12 5.11 5.11 5.08 5.08 5.08 5.01 5.01 5.01 4.99 4.99 4.99 4.89 4.89 4.88 4.88 4.88 4.88 4.85 4.85 4.84 3.32 3.31 3.06 3.06 2.82 2.80 2.79 2.77 2.63 2.62 2.62 2.31 2.29 2.16 1.80 1.77 1.74 1.74 1.74 1.72 1.71 1.71 1.70 1.69 1.68 1.68 1.67 1.67 1.53 1.45 1.45 1.44 1.43 1.43 1.41 1.39 1.37 1.22 1.20 1.20 1.19 1.18 1.17 1.17 1.12 0.91 0.90 0.86 0.86 0.85 0.76 0.74
H
OH
COOMe
CH3
1.07
1.14 1.15 2.23
3.17 1.07 1.09 1.11 1.10 1.12 1.20 1.16 1.28 3.31 3.55 1.38 2.07 1.31 2.17 3.24 3.37 3.37 1.32
10.0 9.5 9.0 8.5 8.0 7.5 7.0 6.5 6.0 5.5 5.0 4.5 4.0 3.5 3.0 2.5 2.0 1.5 1.0 0.5
Chemical Shift (ppm)

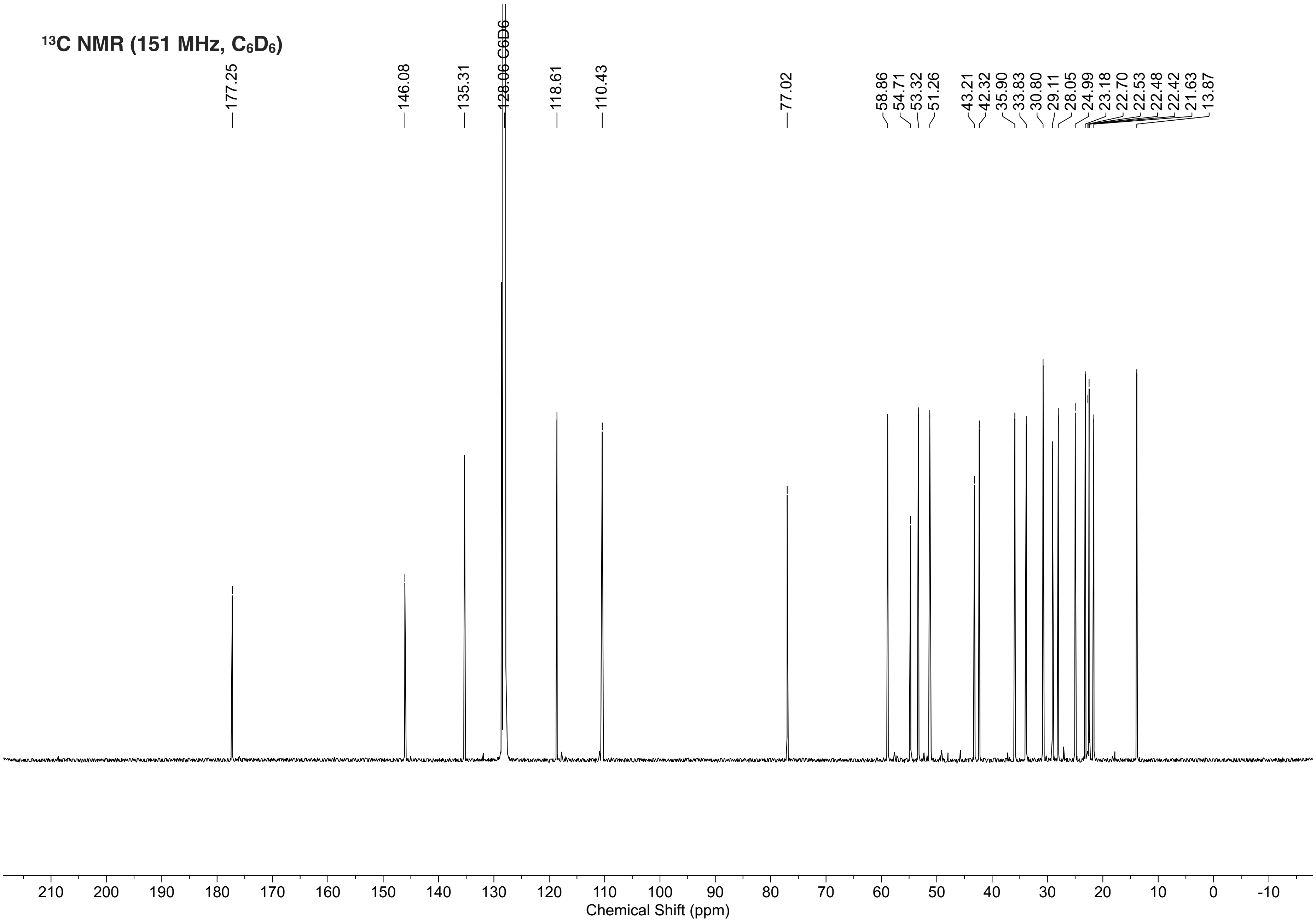

13C NMR (151 MHz, C6D6)
177.25
146.08
135.31
128.06 C6D6
118.61
110.43
77.02
58.86
54.71
53.32
51.26
43.21
42.32
35.90
33.83
30.80
29.11
28.05
24.99
23.18
22.70
22.53
22.48
22.42
21.63
13.87
210
200
190
180
170
160
150
140
130
120
110
100
90
80
70
60
50
40
30
20
10
0
-10
Chemical Shift (ppm)

Hydroxy-Ester **16**

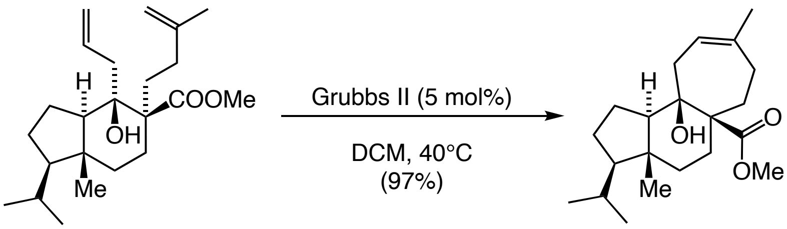

15 **16**

CCDC: 2390964

To a flame-dried–500 mL round bottom flask with stir bar was added **15** (159 mg, 0.438 mmol, 1 equiv.) in anhydrous DCM (219 mL, 2 mM).* Grubbs II catalyst (18.6 mg, 21.9 µmol, 5 mol%) was added while stirring. The reddish–brown solution was placed on a heating mantle and covered with aluminum foil at 40°C overnight. After 12 hours, the mud–brown reaction mixture was removed from heating. TLC analysis and crude NMR analysis indicated consumption of starting material, and the reaction was quenched with DMSO (155 µL, 2.19 mmol, 5.0 equiv.) (40 equiv. relative to Grubbs II) and left to stir for 8 hours. The solution was concentrated *in–vacuo* to afford a black solution that was purified by flash column chromatography with (10% Et-$_2$O/Hexanes) to afford **16** as a white crystalline solid (142 mg, 0.425 mmol) in 97% yield.

$[\alpha]_D^{23}$ **=** –21.6° (c = 0.37, $CHCl_3$)

^{1}H NMR (600 MHz, $CDCl_3$): δ 5.34 (ddq, *J* = 8.6, 5.0, 1.6 Hz, 1H), 4.26 (s, 1H), 3.72 (s, 3H), 2.81 (ddd, *J* = 14.4, 5.3, 2.4 Hz, 1H), 2.29 (td, *J* = 14.0, 4.0 Hz, 1H), 2.14 (t, *J* = 13.3 Hz, 1H), 2.02 (dd, *J* = 14.4, 8.8 Hz, 1H), 1.94 (ddd, *J* = 13.7, 11.9, 1.6 Hz, 1H), 1.89 – 1.76 (m, 4H), 1.75 – 1.71 (m, 2H), 1.70 (s, 3H), 1.65 – 1.54 (m, 4H), 1.53 – 1.38 (m, 4H), 1.32 – 1.23 (m, 1H), 1.00 (t, overlapped, 1H), 0.97 (s, 3H), 0.89 (d, *J* = 6.5 Hz, 3H), 0.83 (d, *J* = 6.6 Hz, 3H).

^{13}C NMR (151 MHz, $CDCl_3$): δ 179.4, 141.5, 120.9, 75.1, 59.3, 56.5, 51.6, 49.2, 43.0, 38.1, 35.6, 32.1, 31.0, 30.6, 29.8, 27.8, 25.3, 23.1, 22.4, 19.9, 14.1.

HRMS (ESI): m/z $[M+H]^+$: calcd for $C_{21}H_{34}O_3^+$: 335.2581, found: 335.2596.

IR (Diamond–ATR, neat): v_{max} (cm^{-1}): 3505, 2943, 2873, 1696, 1448, 1315, 1251,1009, 817, 531, 667, 440.

R_f (EtOAc/Hex = 10%): 0.58 (*p*-anisaldehyde: purple)

m_p: 96.3 – 99.2°C

***Note:** On larger scales (>250 mg), the concentration of DCM was increased to 0.05 M without noticeable deterioration in yield (typically 93% isolated yield).

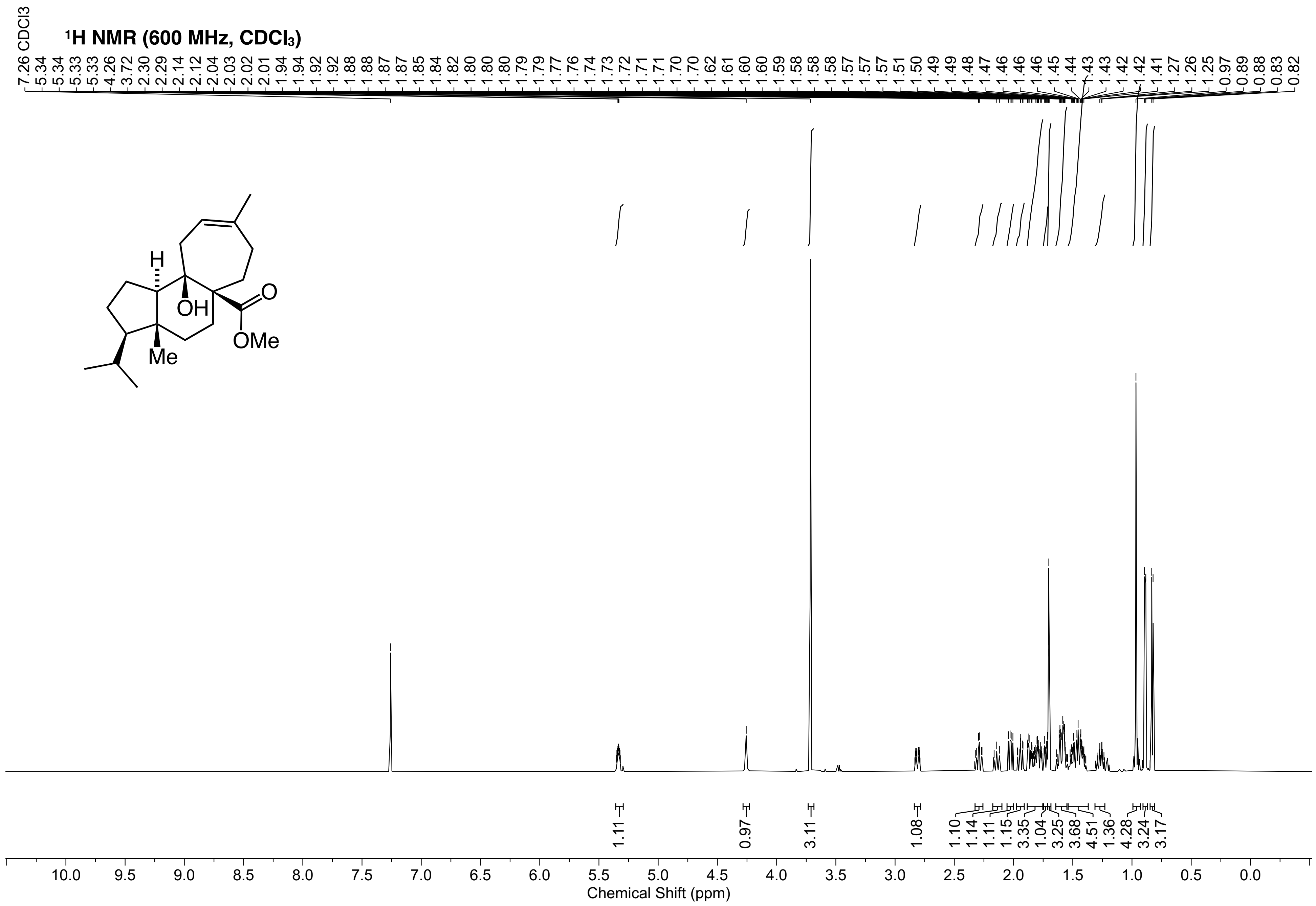
1H NMR (600 MHz, CDCl3)
7.26 CDCl3
5.34 5.34 5.33 5.33 4.26 3.72 2.30 2.29 2.14 2.12 2.04 2.03 2.02 2.01 1.94 1.92 1.92 1.88 1.88 1.87 1.87 1.85 1.84 1.82 1.80 1.80 1.80 1.79 1.79 1.77 1.76 1.74 1.73 1.72 1.71 1.71 1.70 1.70 1.62 1.61 1.60 1.60 1.59 1.58 1.58 1.58 1.57 1.57 1.57 1.51 1.50 1.49 1.49 1.48 1.47 1.46 1.46 1.46 1.45 1.44 1.43 1.43 1.42 1.42 1.41 1.27 1.26 1.25 0.97 0.89 0.88 0.83 0.82
H
OH
O
OMe
Me
1.11 0.97 3.11 1.08 1.10 1.14 1.11 1.15 3.35 1.04 3.25 3.68 4.51 1.36 4.28 3.24 3.17
10.0 9.5 9.0 8.5 8.0 7.5 7.0 6.5 6.0 5.5 5.0 4.5 4.0 3.5 3.0 2.5 2.0 1.5 1.0 0.5 0.0
Chemical Shift (ppm)

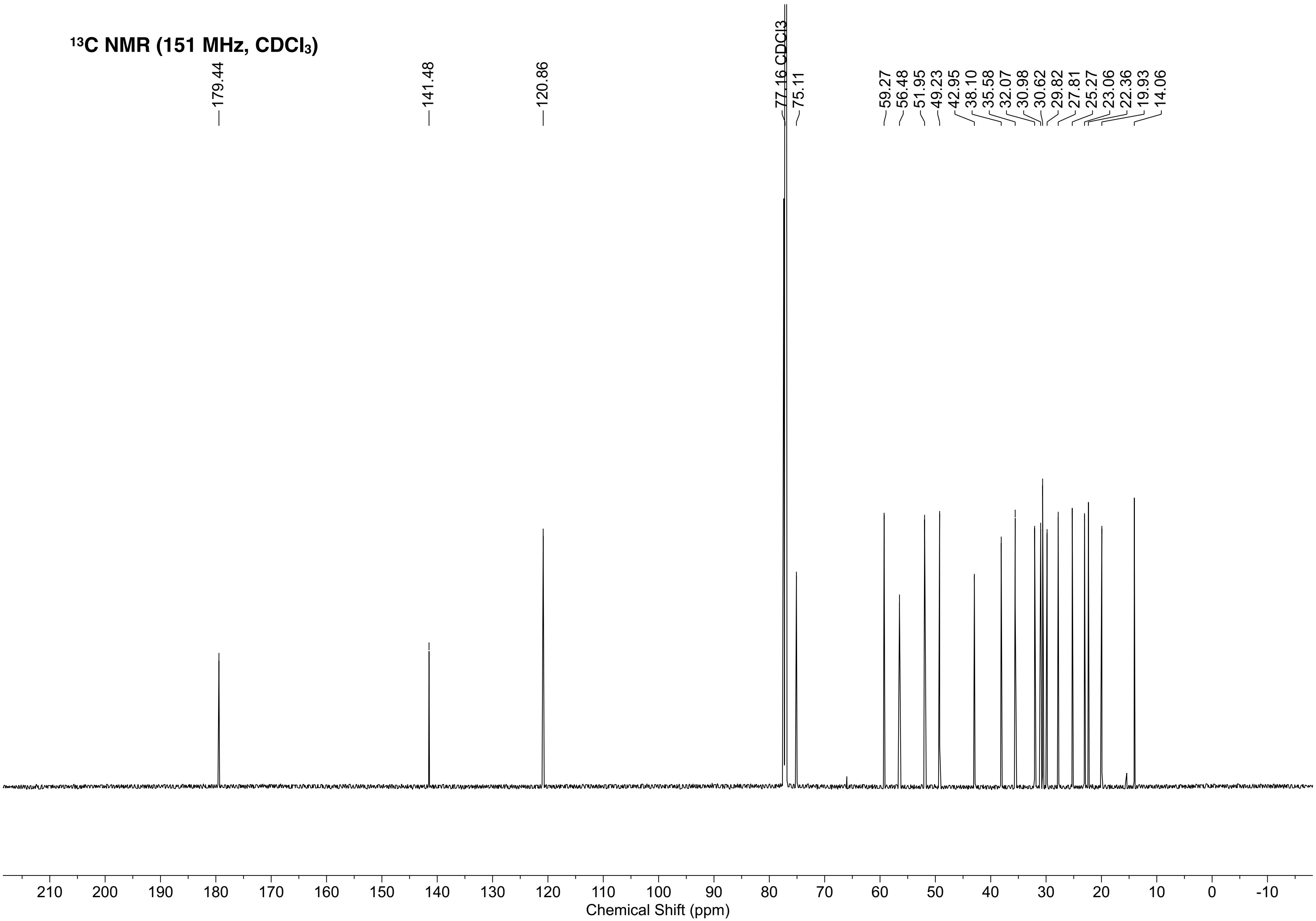
13C NMR (151 MHz, CDCl3)
179.44
141.48
120.86
77.16 CDCl3
75.11
59.27
56.48
51.95
49.23
42.95
38.10
35.58
32.07
30.98
30.62
29.82
27.81
25.27
23.06
22.36
19.93
14.06
210 200 190 180 170 160 150 140 130 120 110 100 90 80 70 60 50 40 30 20 10 0 -10
Chemical Shift (ppm)

Hydroxy-Acid **17**

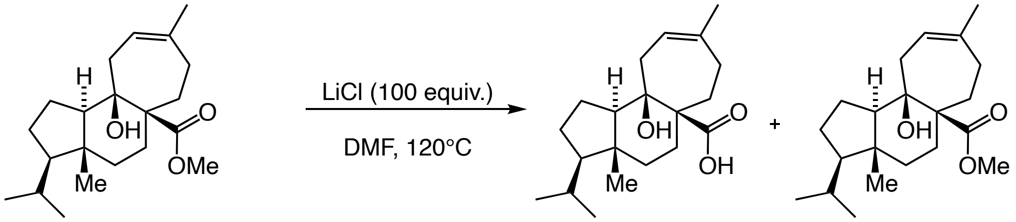

16   **17** (66%)   **16** (16% RSM)

To a 20 mL microwave vial equipped with a stir bar was added LiCl (1.65 g, 100 equiv. 38.9 mmol) The vial was crimped, flame-dried under vacuum, and sparged with argon. The reaction vessel was evacuated and backfilled with argon twice more. **16** (130 mg, 1.0 equiv. 0.389 mmol) as a solution in DMF (7.77 mL, 0.05 M), was added to the reaction mixture and allowed to stir. The reaction was heated to 120°C for 48 hours. After cooling to room temperature, the reaction mixture was diluted with EtOAc (10 mL) and quenched with 1 M aqueous HCl (10 mL). The aqueous layer was extracted with EtOAc (3 x 10 mL), washed with brine (6 x 10 mL), dried over anhydrous Na_2SO_4, filtered, and concentrated *in–vacuo*. Flash column chromatography in 20% EtOAc/Hexanes + 1% AcOH provided a pale-yellow oil that was azeotroped with heptanes (3x3 mL) to yield a white crystalline solid **17** (82 mg, 0.260 mmol) in 66% yield along with **16** (21 mg, 0.062 mmol) in 16% yield of the starting material recovered.

$[\alpha]_D^{23}$ = –15.3° (c = 0.13, $CHCl_3$).

^{1}H NMR (600 MHz, C_6D_6): **δ** 5.38 – 5.30 (m, 1H), 2.94 (dd, *J* = 14.4, 2.4 Hz, 1H), 2.46 (td, *J* = 10.0, 4.0 Hz, 1H), 2.41 (t, *J* = 13.7 Hz, 1H), 2.05 (dd, *J* = 14.4, 8.8 Hz, 1H), 1.98 (ddd, *J* = 14.6, 7.0, 1H), 1.91 (t, *J* = 1H), 1.75 – 1.62 (m, 5H), 1.60 (s, 3H), 1.54 (dt, *J* = 13.8, 3.5 Hz, 1H), 1.43 (dp, *J* = 10.2, 6.5 Hz, 2H), 1.36 (td, *J* = 13.6, 3.7 Hz, 1H), 1.24 – 1.16 (m, 1H), 1.07 (s, 3H), 0.86 (d, *J* = 6.5 Hz, 3H), 0.80 (d, *J* = 6.7 Hz, 3H), app. 0.80–0.75 (m, 1H) overlapped with d.

Note: *Hydroxyl proton (OH) and carboxylic acid proton (COOH) could not be detected.*

^{13}C NMR (151 MHz, C_6D_6): **δ** 182.1, 142.0, 120.7, 75.8, 59.3, 56.3, 49.7, 43.2, 38.7, 35.7, 32.6, 31.3, 30.7, 29.9, 27.9, 25.2, 23.1, 22.4, 20.0, 14.3.

IR (Diamond–ATR, neat): v_{max} (cm^{-1}): 3401, 2941, 2867, 1689, 1451, 1438, 1381,1364, 1235,1193, 1121,1004, 791,756, 724, 541.

HRMS (ESI): m/z $[M+Na]^+$: calcd for $C_{20}H_{32}O_3Na^+$: 343.2224, found: 343.2224.

R_f (EtOAc/Hex = 30%): 0.38 streaky (*p*-anisaldehyde: dark blue)

m$_p$: 180. – 183°C (melts with decomposition)

***Note**: the compound exhibits decent solubility in C_6D_6, but provided better separation of peaks compared to $CDCl_3$, thus was the preferred solvent of choice for NMR analysis. The ^{1}HNMR in $CDCl_3$ is provided here.

^{1}H NMR (400 MHz, $CDCl_3$): **δ** 5.45 (t, *J* = 6.8 Hz, 1H), 3.05 (dd, *J* = 14.5, 4.5 Hz, 1H), 2.55 (dt, *J* = 18.9, 14.0 Hz, 2H), 2.23 – 2.05 (m, 2H), 1.98 (t, *J* = 12.4 Hz, 1H), 1.85 – 1.74 (m, 5H), 1.71 (s, 3H), 1.64 (dt, *J* = 13.7, 3.4 Hz, 1H), 1.60 – 1.41 (m, 3H), 1.36 – 1.25 (m, 1H), 1.18 (s, 3H), 0.97 (d, *J* = 6.5 Hz, 3H), 0.90 (d, *J* = 6.5 Hz, 3H) app. 0.90–0.84 (m, 1H) overlapped.

¹H NMR (600 MHz, C_6D_6):

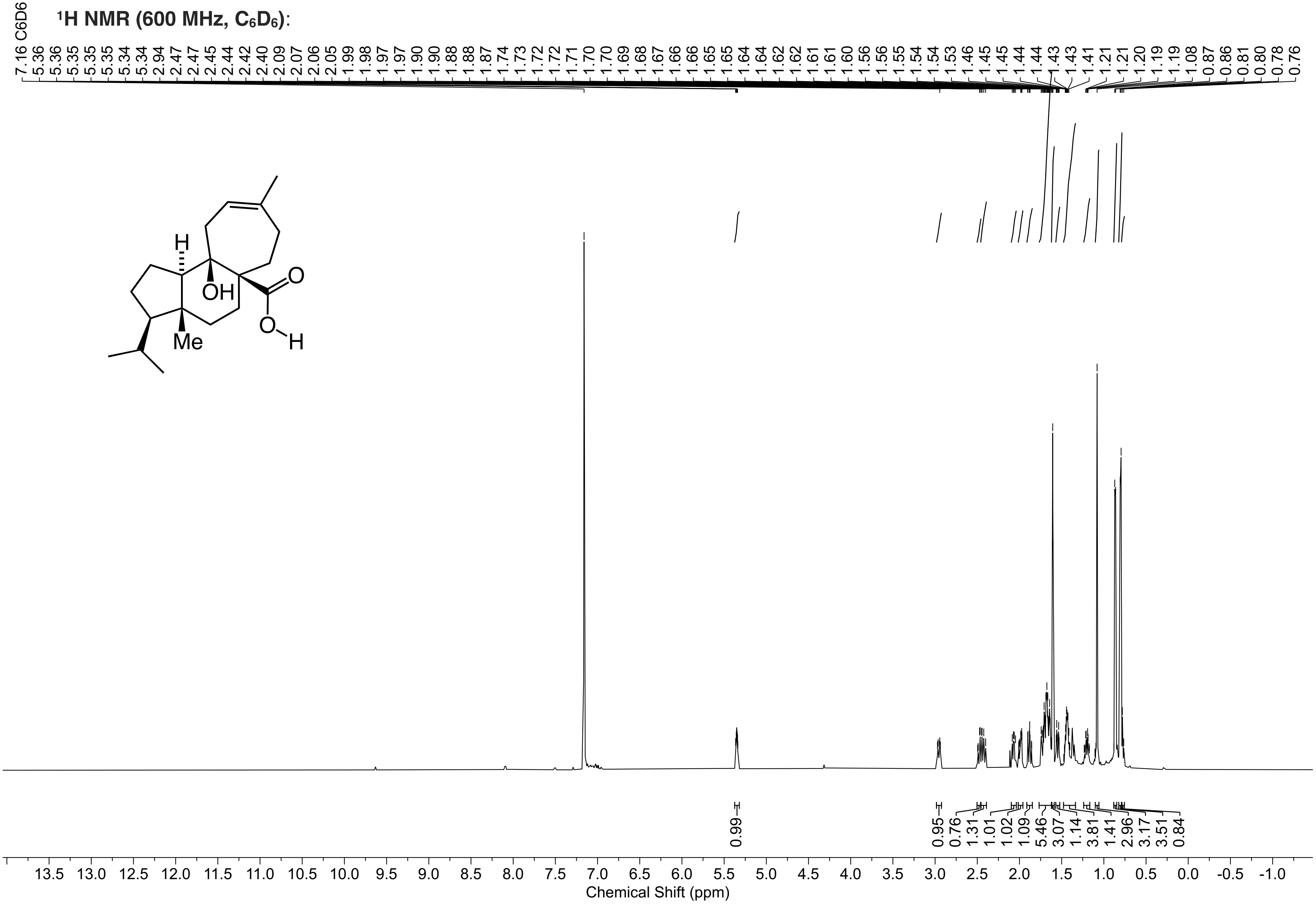

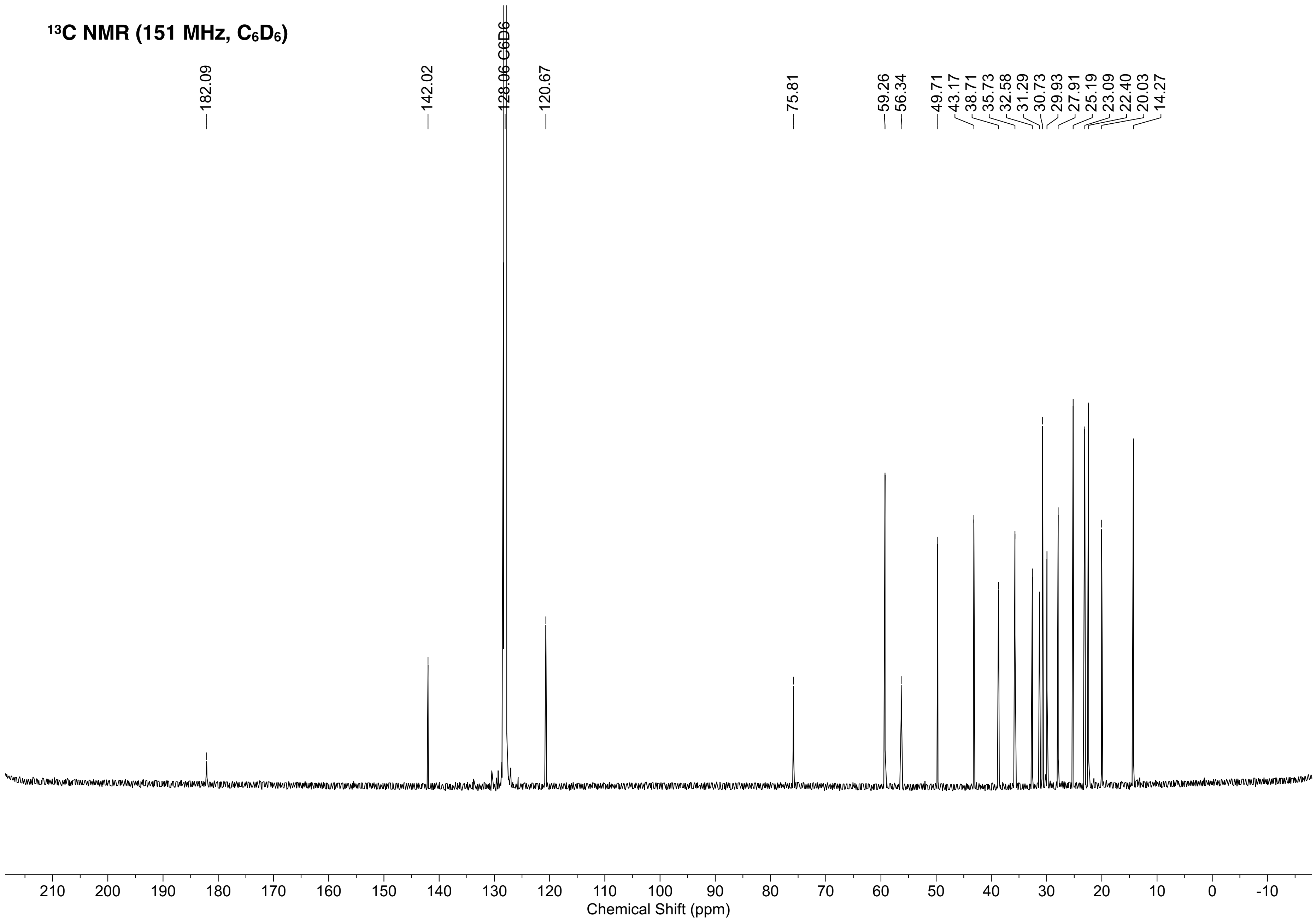

^{13}C NMR (151 MHz, C_6D_6)
182.09
142.02
128.06 C6D6
120.67
75.81
59.26
56.34
49.71
43.17
38.71
35.73
32.58
31.29
30.73
29.93
27.91
25.19
23.09
22.40
20.03
14.27
210 200 190 180 170 160 150 140 130 120 110 100 90 80 70 60 50 40 30 20 10 0 -10
Chemical Shift (ppm)

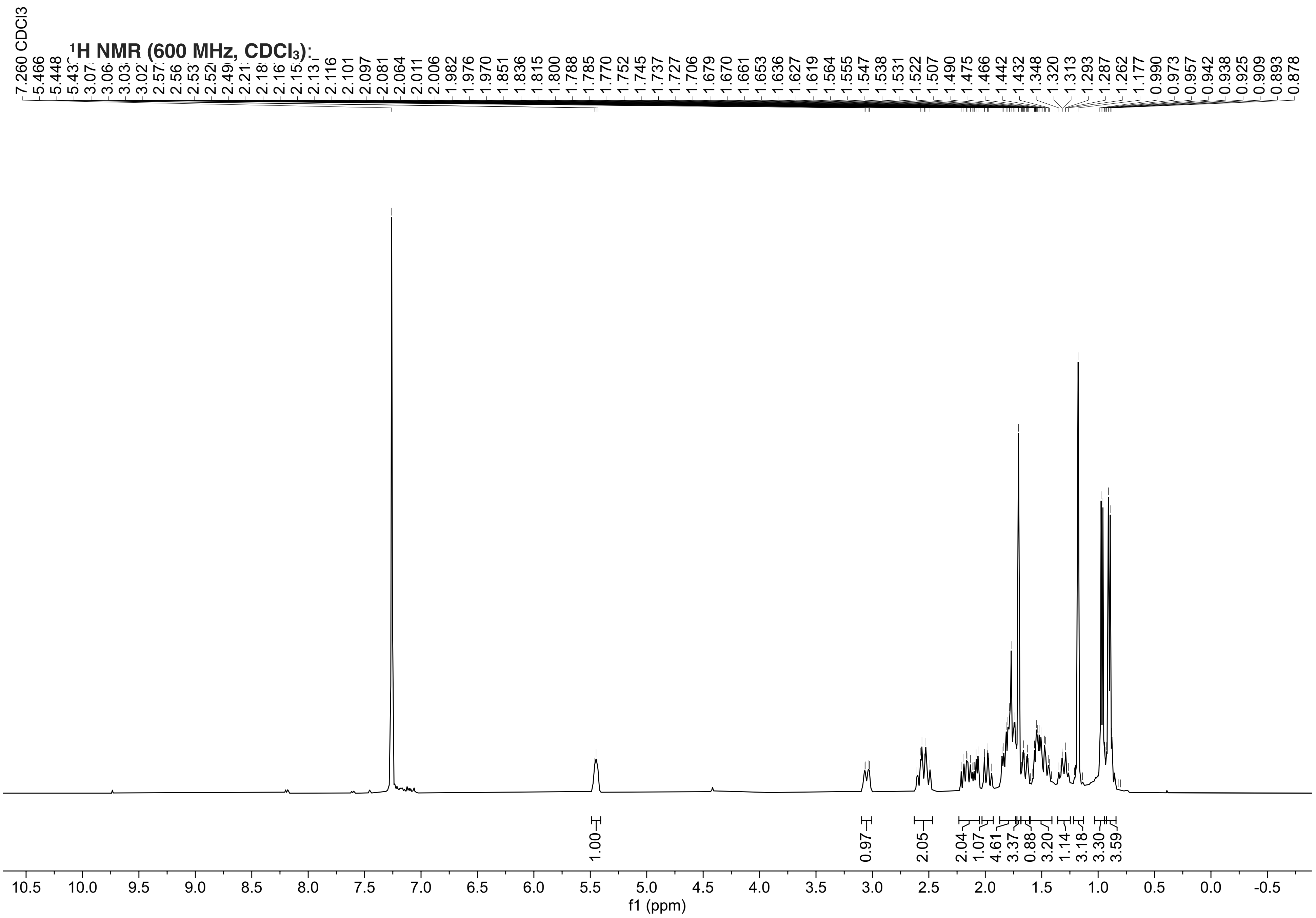

¹H NMR (600 MHz, CDCl₃):
7.260 CDCl3
f1 (ppm)

δ–Lactone **18** and tetrasubstituted alkene **19**

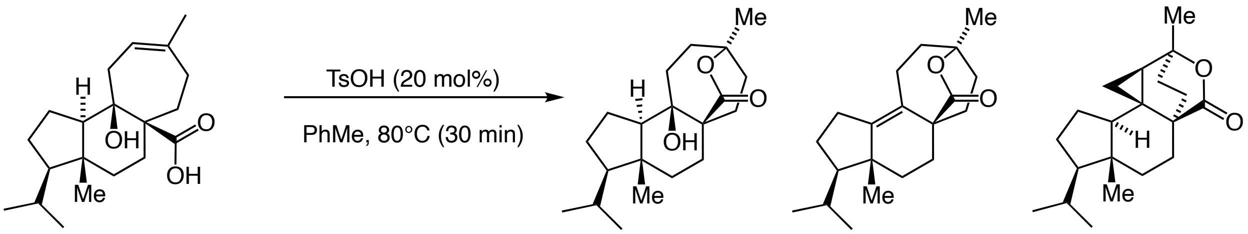

17 **18** (62%) **CCDC:** 2390965 **19** (32%) **azorellolide** *(trace)*

To a flame–dried screw–cap 1 dram vial with a stir bar was added **17** (10.0 mg, 31 µmol, 1 equiv.), dissolved in toluene (3.1 mL, 0.01 M). *p*-Toluenesulfonic acid monohydrate (1.1 mg, 5.8 µmol, 20 mol%) was added. The mixture was stirred at room temperature for 5 minutes. Subsequently, the reaction mixture was heated from room temperature to 80°C and stirred for 30 minutes.* The reaction was cooled to room temperature and quenched with saturated $NaHCO_3$ (1 mL). The organic layer was separated, and the aqueous layer was extracted with EtOAc (3 x 2 mL). The organic layers were combined and washed with brine (2 mL). The brine layer was back-extracted with EtOAc (3 x 1 mL). The organic layer was dried over anhydrous Na_2SO_4, filtered, and concentrated *in–vacuo*. The crude product mixture was then purified by flash column chromatography using 30–50% Et_2O/Pentanes to afford **18** as a white crystalline solid (6.2 mg, 19 µmol) in a 62% yield, **19** (3.0 mg, 9.8 µmol) in a 32% yield as a white solid), and trace **azorellolide** as a thin film (characterized fully on pages 49-51 of SI) (< 0.5 mg).

The following set of characterization is for the **delta–lactone 18**

$[\alpha]_D^{23}$ **=** +7.81° (c = 0.64, $CHCl_3$).

^{1}H NMR (600 MHz, $CDCl_3$): δ 2.86 (td, *J* = 15.0, 4.0 Hz, 1H), 2.37 – 2.27 (m, 1H), 2.13 (td, *J* = 10.2, 4.0 Hz, 1H), 1.92 (app t, *J* = ~8.5 Hz, 2H), 1.90 – 1.83 (m, 2H), 1.73 (ddd, *J* = 14.5, 4.8, 2.6 Hz, 1H), 1.68 (dd, *J* = 8.7, 5.0 Hz, 1H), 1.65 – 1.42 (m, 7H), 1.37 (s, 3H), 1.36 – 1.29 (m, 2H), 1.19 (ddd, *J* = 14.8, 13.1, 3.4 Hz, 1H), 0.99 – 0.94 (m, 1H), 0.93 (s, 3H), 0.91 (d, *J* = 6.5 Hz, 3H), 0.84 (d, *J* = 6.6 Hz, 3H).

^{13}C NMR (151 MHz, $CDCl_3$): δ 176.2, 79.9, 74.8, 59.1, 54.1, 48.8, 42.4, 36.3, 35.4, 34.9, 30.5, 30.3, 30.1, 29.5, 27.5, 27.0, 23.0, 22.4, 19.7, 12.4.

IR (Diamond–ATR, thin film): ν_{max} (cm^{-1}): 3445, 2927, 2873,1770,1459, 1377, 1281,1199, 1154, 1125, 1100, 995, 969.

HRMS (ESI): m/z $[M+Na]^+$: calcd for $C_{20}H_{33}O_3Na^+$: 343.2224, found: 343.2224.

R_f (EtOAc/Hex = 30%): 0.42 (*p*-anisaldehyde: dark blue)

m_p: 101.3 – 104.5°C

Characterization for Compound **19**

$[\alpha]_D^{23}$ **=** +20.0° (c = 0.45, $CHCl_3$).

^{1}H NMR (600 MHz, $CDCl_3$): δ 2.62 (td, *J* = 13.6, 1.6 Hz, 1H), 2.41 (dt, *J* = 15.5, 6.0 Hz, 1H), 2.20 (t, *J* = 7.7 Hz, 2H), 2.18 – 2.08 (m, 2H), 1.98 – 1.90 (m, 2H), 1.90 – 1.85 (m, 3H), 1.84 – 1.72 (m, 2H), 1.60-1.55 (m, 1H) overlapped with H_2O, 1.49 (td, *J* = 8.8, 3.2 Hz, 1H), 1.47–1.37 (overlapped m, 2H), 1.41 (s, 3H), 1.41 – 1.37 (m, 1H), 1.12 – 1.06 (q, *J* = 8.8 Hz, 1H), 0.97 (d, *J* = 6.6, Hz, 3H), 0.91 (s, 3H), 0.90 (d, *J* = 6.6, Hz, 3H).

^{13}C NMR (151 MHz, $CDCl_3$): δ 176.5, 145.1, 125.7, 81.2, 57.9, 45.2, 43.8, 37.5, 33.8, 33.6, 33.5, 31.5, 30.6, 30.2, 27.7, 26.6, 26.4, 23.0, 22.8, 18.8.

IR (Diamond–ATR, thin film): ν_{max} (cm^{-1}): 3053, 2960, 1722, 1446, 1205, 1099, 911.

HRMS (ESI): m/z $[M+H]^+$**:** calcd for $C_{20}H_{30}O_2^+$: 303.2335, found: 303.2319.

R_f (EtOAc/Hex = 20%): 0.35 (*p*-anisaldehyde: dark blue)

***Note:** If the reaction is left to stir for 10 minutes rather than 30 minutes, the yield of **18** can be substantially improved (typically >90% isolated yield).

[1] H NMR (600 MHz, $CDCl_3$)

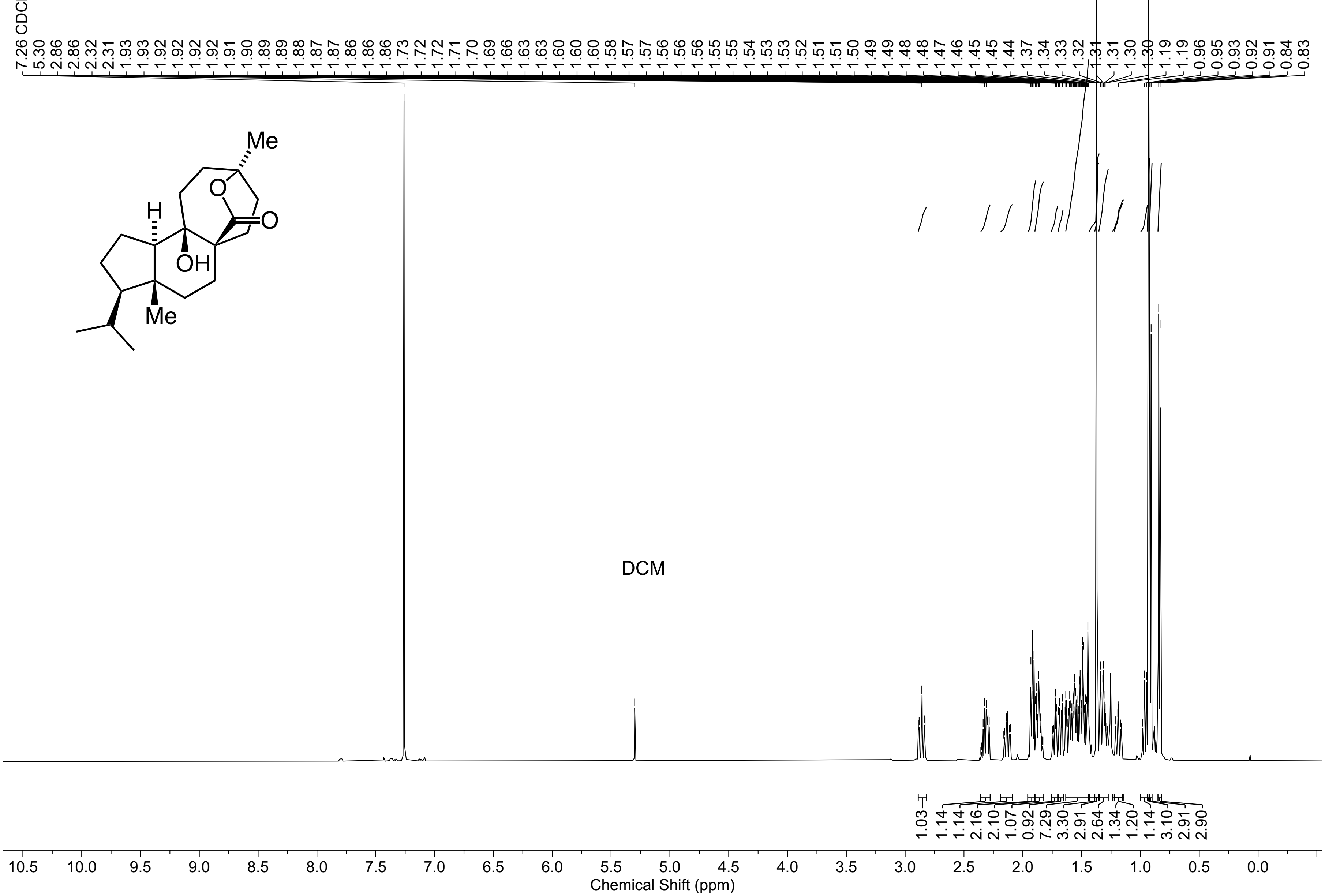

^{13}C NMR (151 MHz, $CDCl_3$)

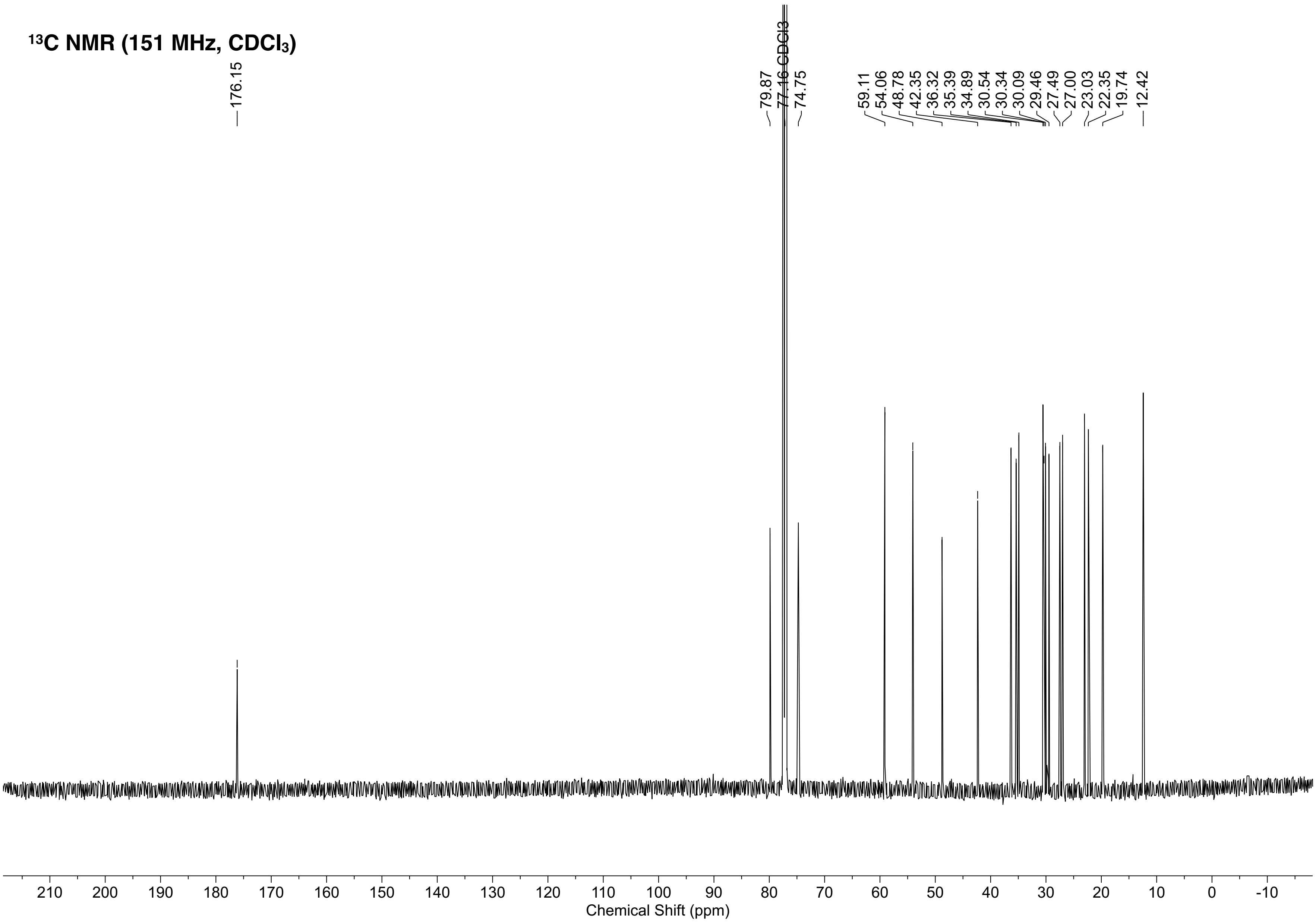

¹H NMR (600 MHz, CDCl₃)

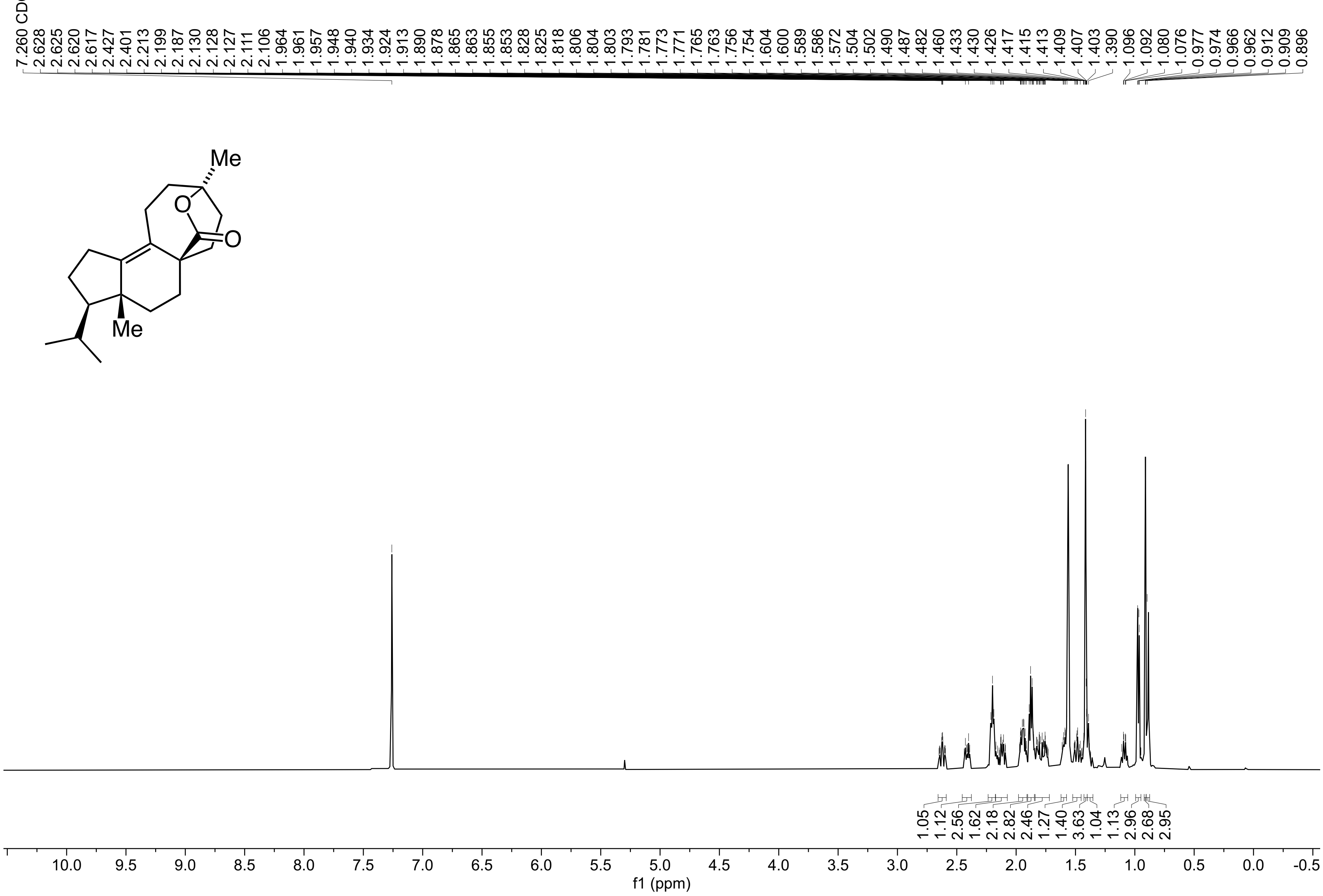

^{13}C NMR (151 MHz, $CDCl_3$)

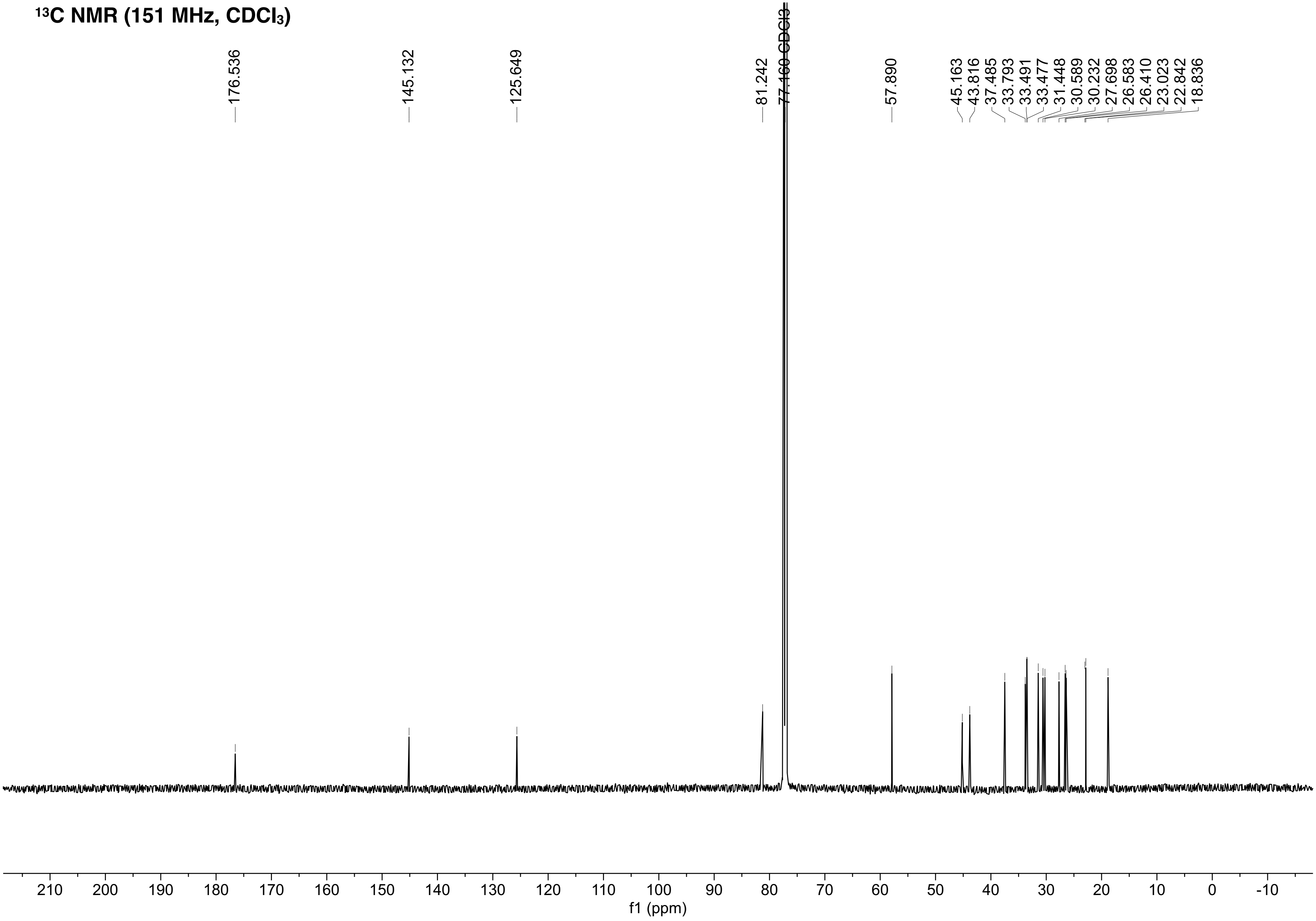

β-lactone **8**

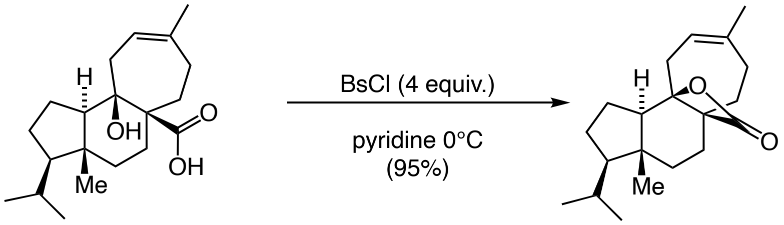

17 **8**

CCDC: 2390963

To a 1 dram flame–dried screw cap vial with stir bar was added **17** (10.0 mg, 31.2 µmol, 1 equiv.) and dissolved in anhydrous pyridine (0.64 mL, 0.05 M) under argon. This was set up 2 more times in parallel as a batch reaction (total: 30.0 mg, 93.6 µmol). The reactions were cooled to 0°C. Meanwhile, benzenesulfonyl chloride (16.1 µL, 4 equiv., 0.12 mmol) (total: 48.3 µL, 0.36 mmol) was added dropwise over 30 seconds to each reaction vessel, and the reactions were left to stir for 90 minutes at 0°C. Upon consumption the starting material, the reactions are quenched with ice (~ 1 g to each reaction vessel), combined, and extracted with Et_2O (3 x 3 mL). The organic extract is washed with saturated aqueous $CuSO_4$ (2 x 3 mL), brine (1 x 3 mL), dried over anhydrous Na_2SO_4, filtered, and concentrated *in–vacuo* to yield yellow crystals. Crystals were taken up in EtOAc (5 x 3 mL) and filtered through a disposable pipette column with (9:1 Celite®: activated charcoal) (500 mg). Concentration *in–vacuo* provided **8** as white crystals (26.8 mg, 29.6 µmol), 95% yield.
***Note:** Compound **8** degraded if not properly stored at –20°C as a solution in benzene.

$[\alpha]_D^{23}$ **=** –28.7° (c = 0.24, $CHCl_3$).

^{1}H NMR (600 MHz, $CDCl_3$): **δ** 5.25 – 5.19 (m, 1H), 2.83 (ddq, *J* = 15.0, 4.7, 2.4 Hz, 1H), 2.57 (ddd, *J* = 14.4, 11.9, 5.0 Hz, 1H), 2.39 (dd, *J* = 15.1, 9.3 Hz, 1H), 2.29 – 2.21 (m, 2H), 2.20 – 2.14 (m, 1H), 2.14 – 2.07 (m, 1H), 1.93 (dtd, *J* = 13.4, 9.4, 6.1 Hz, 1H), 1.78 – 1.72 (m, 2H), 1.68 – 1.60 (m, 2H), 1.64 (s, 3H), 1.60 – 1.49 (m, 3H), 1.34 (dddd, *J* = 13.4, 11.6, 9.9, 3.6 Hz, 1H), 1.15 – 1.09 (m, 1H), 1.03 (q, *J* = 9.6 Hz, 1H), 0.92 (d, *J* = 6.6 Hz, 3H), 0.85 (d, *J* = 6.6 Hz, 3H), 0.78 (s, 3H).

^{13}C NMR (151 MHz, $CDCl_3$): δ 176.4, 138.2, 117.0, 87.7, 58.0, 54.6, 50.6, 41.7, 36.7, 32.6, 30.7, 30.5, 27.7, 27.6, 25.7, 24.7, 22.8, 22.3, 19.7, 12.2.

IR (Diamond–ATR, neat): ν_{max}(cm^{-1}): 2814, 1800, 1459, 1380, 1289, 1193, 1125, 846, 790, 723

R_f (EtOAc/Hex = 20%): 0.58 (*p*-anisaldehyde: dark blue)

HRMS (ESI) = m/z [M+H]$^+$: calcd for $C_{20}H_{31}O_2^+$: 303.2324, found: 303.2327

m$_p$: 95.9 – 98.2°C

^1H NMR (600 MHz, $CDCl_3$)

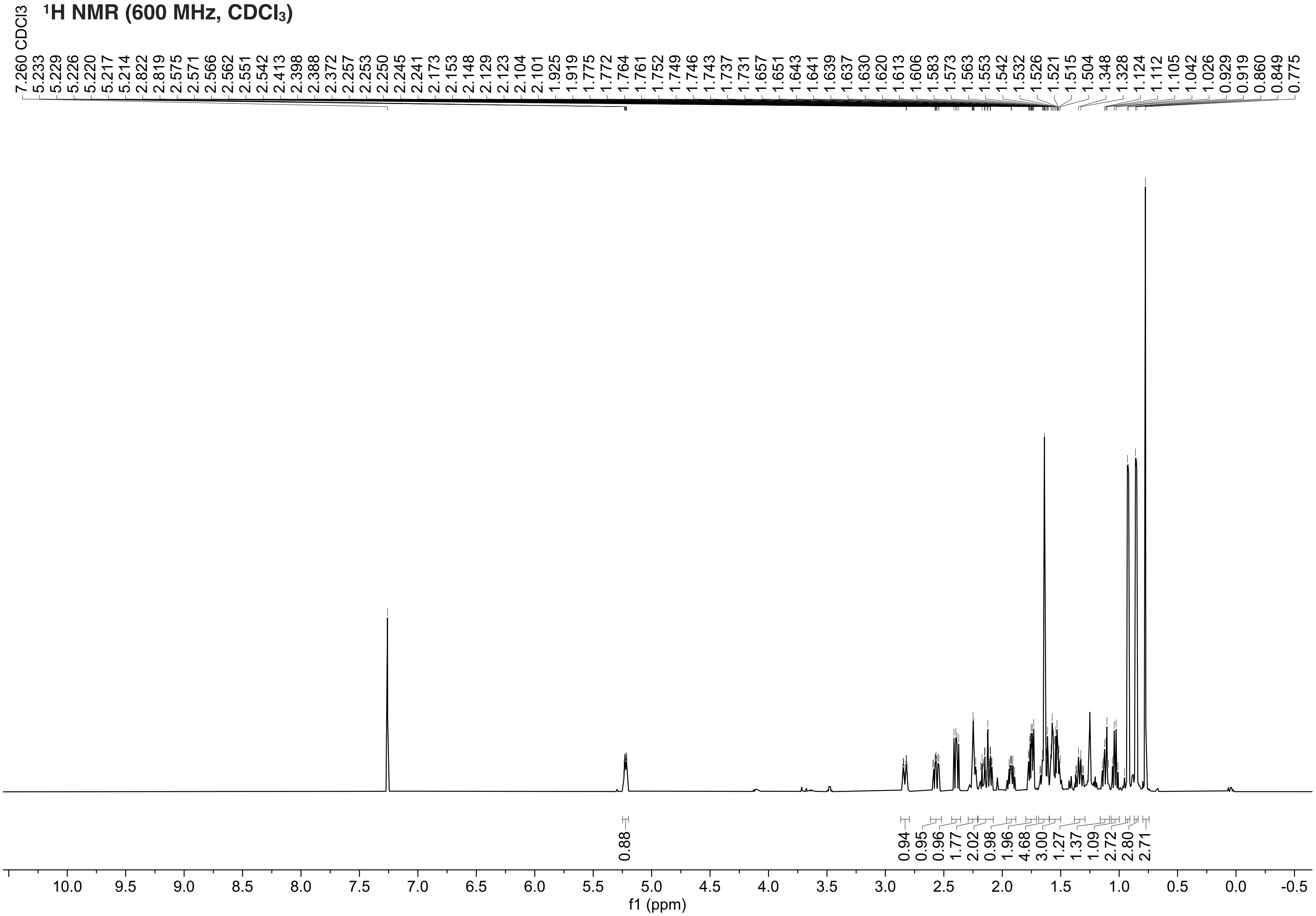

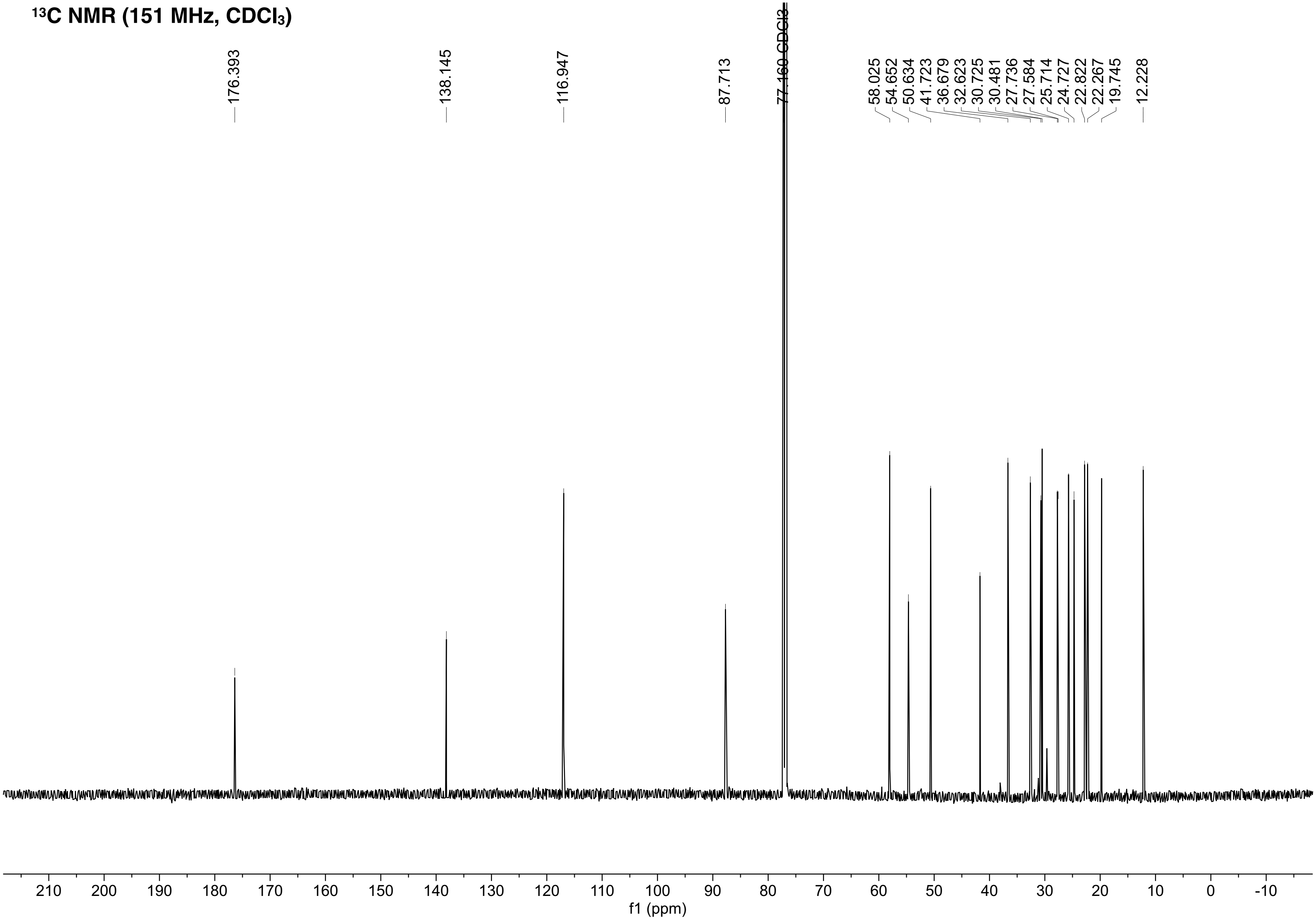
13C NMR (151 MHz, CDCl3)
176.393
138.145
116.947
87.713
77.160 CDCl3
58.025
54.652
50.634
41.723
36.679
32.623
30.725
30.481
27.736
27.584
25.714
24.727
22.822
22.267
19.745
12.228
210 200 190 180 170 160 150 140 130 120 110 100 90 80 70 60 50 40 30 20 10 0 -10
f1 (ppm)

Dyotropic shift **21**

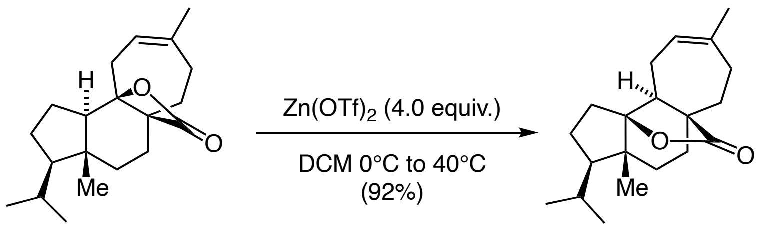

8

21
CCDC: 2390967

To a flame–dried 1 dram vial with septa–cap, equipped with a stir bar, was added **8** (5.0 mg, 16.5 µmol, 1 equiv.) in anhydrous DCM (800 µL, 0.02 M). Next, $Zn(OTf)_2$ (24.0 mg, 66.1 µmol, 4.0 equiv.) was added at 0°C and the reaction mixture was left to stir. No reactivity was detected after 15 minutes, thus the reaction was then refluxed overnight. Upon consumption of starting material by TLC analysis, the reaction is concentrated *in–vacuo*, dissolved in a minimal amount of toluene (<1 mL) and purified with flash column chromatography in 10% (Et_2O/Pentanes) to yield **21** as a white crystalline solid (4.6 mg, 15 µmol) in 92% yield.

$[\alpha]_D^{23}$ **=** +16.9° (c = 0.5, $CHCl_3$).

^{1}H NMR (400 MHz, C_6D_6) δ: 5.34 – 5.30 (m, 1H), 2.20 (ddd, *J* = 14.3, 10.6, 1.9 Hz, 1H), 2.02 – 1.93 (m, 2H), 1.92 (s, 1H), 1.92 – 1.84 (m, 2H), 1.81 – 1.68 (m, 2H), 1.69 – 1.62 (m, 1H), 1.64 – 1.60 (m, 1H), 1.59 (s, 3H), 1.46 (ddd, *J* = 14.3, 8.2, 1.9 Hz, 1H), 1.41 – 1.26 (m, 4H), 1.26 – 1.06 (m, 2H), 0.78 (s, 3H), 0.74 (t, *J* = 6.7 Hz, 6H).

^{13}C NMR (101 MHz, $CDCl_3$) δ: 181.3, 140.3, 122.3, 95.7, 57.1, 49.5, 46.5, 46.4, 33.7, 31.5, 29.9, 29.1, 28.8, 27.4, 26.7, 25.9, 24.7, 22.8, 22.6, 15.8.

IR (Diamond–ATR, neat): ν_{max} (cm^{-1}): 2928, 1766, 1454, 1291, 1201, 1170, 1138, 1092, 1061, 998, 969, 953, 887, 797, 588.

HRMS (ESI): m/z $[M+Na]^+$: calcd for $C_{20}H_{30}O_2Na^+$: 325.2143, found: 325.2138.

R_f (EtOAc/Hex = 20%): 0.43 (*p*-anisaldehyde: purple then blue)

m_p: 106.6 – 108.2°C

^{1}H NMR (400 MHz, C_6D_6)

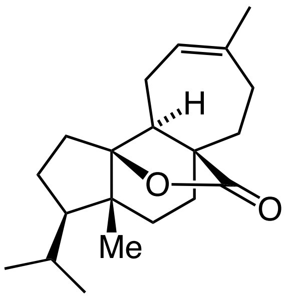

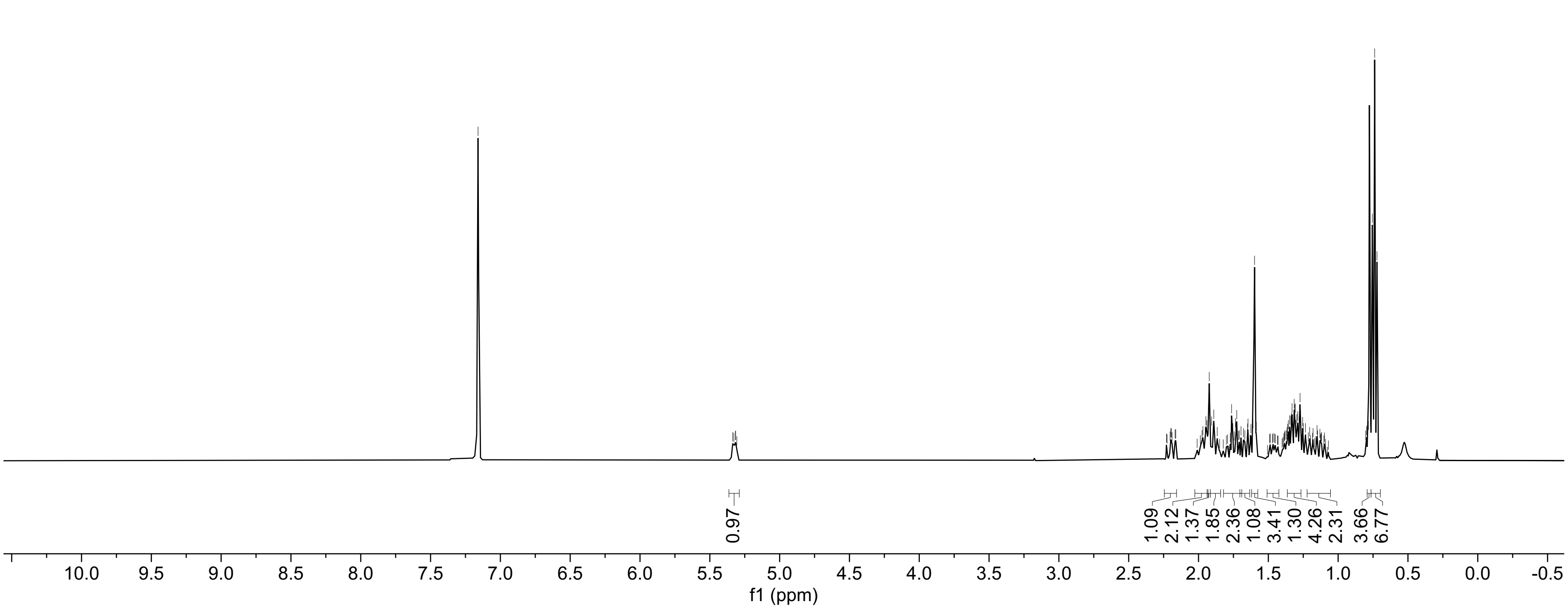

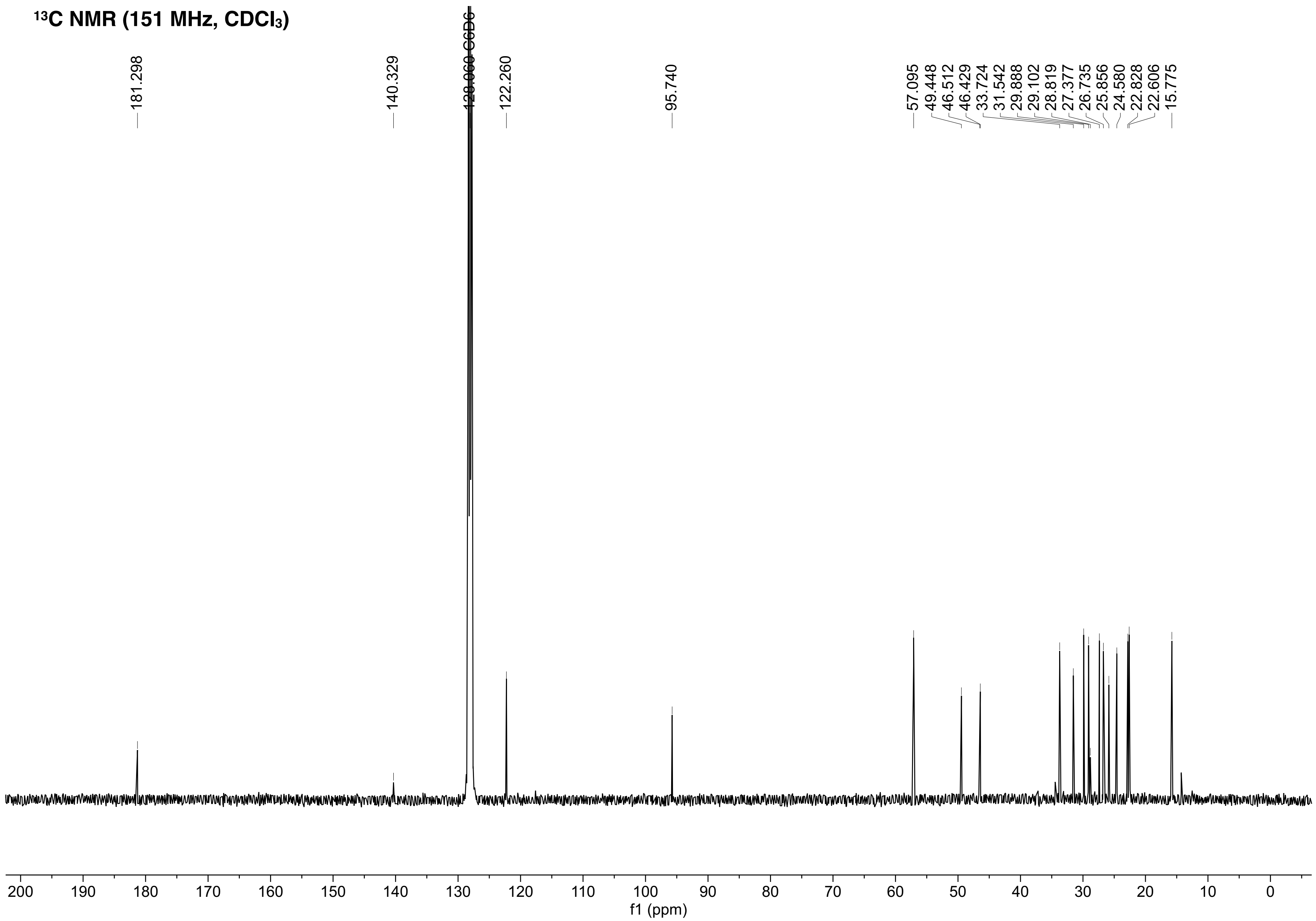

13C NMR (151 MHz, CDCl3)
181.298
140.329
122.260
95.740
57.095
49.448
46.512
46.429
33.724
31.542
29.888
29.102
28.819
27.377
26.735
25.856
24.580
22.828
22.606
15.775
200 190 180 170 160 150 140 130 120 110 100 90 80 70 60 50 40 30 20 10 0
f1 (ppm)

20

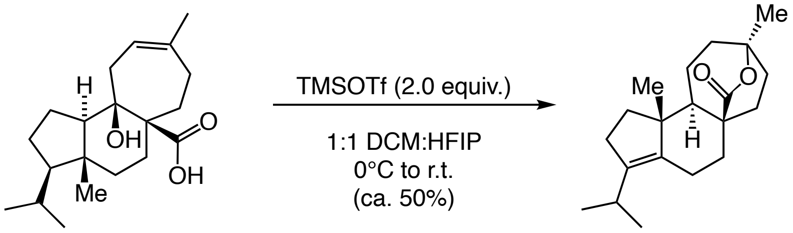

17

20
CCDC: 2390966

To a flame–dried 1 dram vial with septa–cap was added **17** (11.2 mg,34.9 µmol, 1 equiv.) in anhydrous DCM (0.87 mL, 0.02 M) and hexafluoroisopropanol (HFIP) (0.87 mL, 0.02 M) and cooled to 0°C. Trimethylsilyl trifluoromethanesulfonate (15.5 mg, 12.7 µL, 69.9 µmol, 2.1 equiv.) was added as a solution in DCM (100 µL) *via* 250 µL Hamilton® syringe (ca. 1 minute) and left to stir at 0°C. Upon addition, the solution turned a dark yellow. After 20 minutes, TLC analysis indicated consumption of starting material, the reaction was immediately quenched with saturated aqueous $NaHCO_3$ (2 mL) at 0°C and the DCM layer was removed. The aqueous layer was extracted with EtOAc (3 x 2 mL), washed with brine, dried over anhydrous Na_2SO_4, filtered, and concentrated *in–vacuo*. The crude product was purified with flash column chromatography with 10% to 20% (Et_2O/Pentanes) to give **20** as a crystalline solid (5.3 mg, 18 µmol) in 50% yield. ***Note**: after extensive optimization, there is difficulty in separating the compound from other impurities that may be present, however this compound was able to crystalize post purification. It is hypothesized the impurities are double bond isomers around the isopropyl unit and 5-membered ring.
$[\alpha]_D^{23}$ **=** –10.5° (c = 0.1, $CHCl_3$).

^{1}H NMR (600 MHz, $CDCl_3$) δ: 2.77 – 2.69 (m, 1H), 2.63 (p, *J* = 6.8 Hz, 1H), 2.28 (dt, *J* = 15.5, 4.7 Hz, 1H), 2.26 – 2.11 (m, 2H), 2.07 – 2.00 (m, 1H), 1.93 – 1.85 (m, 4H), 1.76 – 1.66 (m, 4H), 1.47 – 1.41 (m, 2H), 1.39 (s, 3H), 1.33 – 1.28 (m, 2H), 1.08 (ddd, *J* = 13.2, 11.8, 4.8 Hz, 1H), 0.96 (d, *J* = 6.8 Hz, 3H), 0.95 (s, 3H), 0.92 (d, *J* = 6.1, Hz, 3H).

^{13}C NMR (151 MHz, $CDCl_3$) δ: 178.3, 138.9, 137.2, 81.3, 55.4, 51.7, 44.3, 40.6, 37.9, 36.9, 34.2, 31.9, 30.7, 27.7, 26.6, 23.2, 21.70, 21.3, 20.9, 19.0.

IR (Diamond–ATR, thin film): v_{max} (cm^{-1}): 2956, 2867, 1726, 1195, 1087, 918, 600.

HRMS (ESI): m/z $[M+Na]^+$: calcd for $C_{20}H_{30}O_2Na^+$: 325.2143, found: 325.2138.

R_f (EtOAc/Hex = 20%): 0.38 (*p*-anisaldehyde: dark blue).

m_p: 110.5 – 112.3°C

^{1}H NMR (600 MHz, CDCl$_3$)

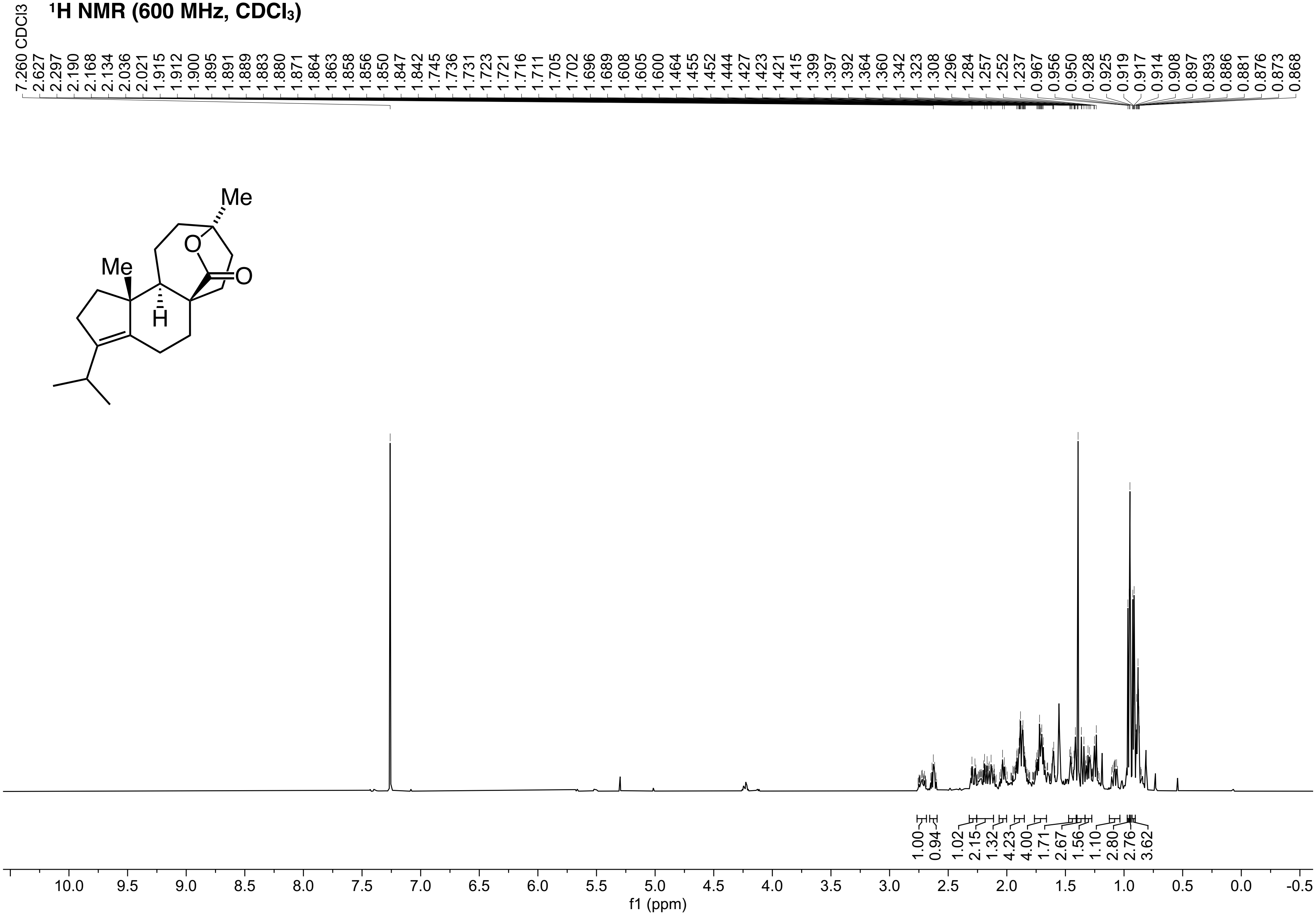

^{13}C NMR (151 MHz, $CDCl_3$)

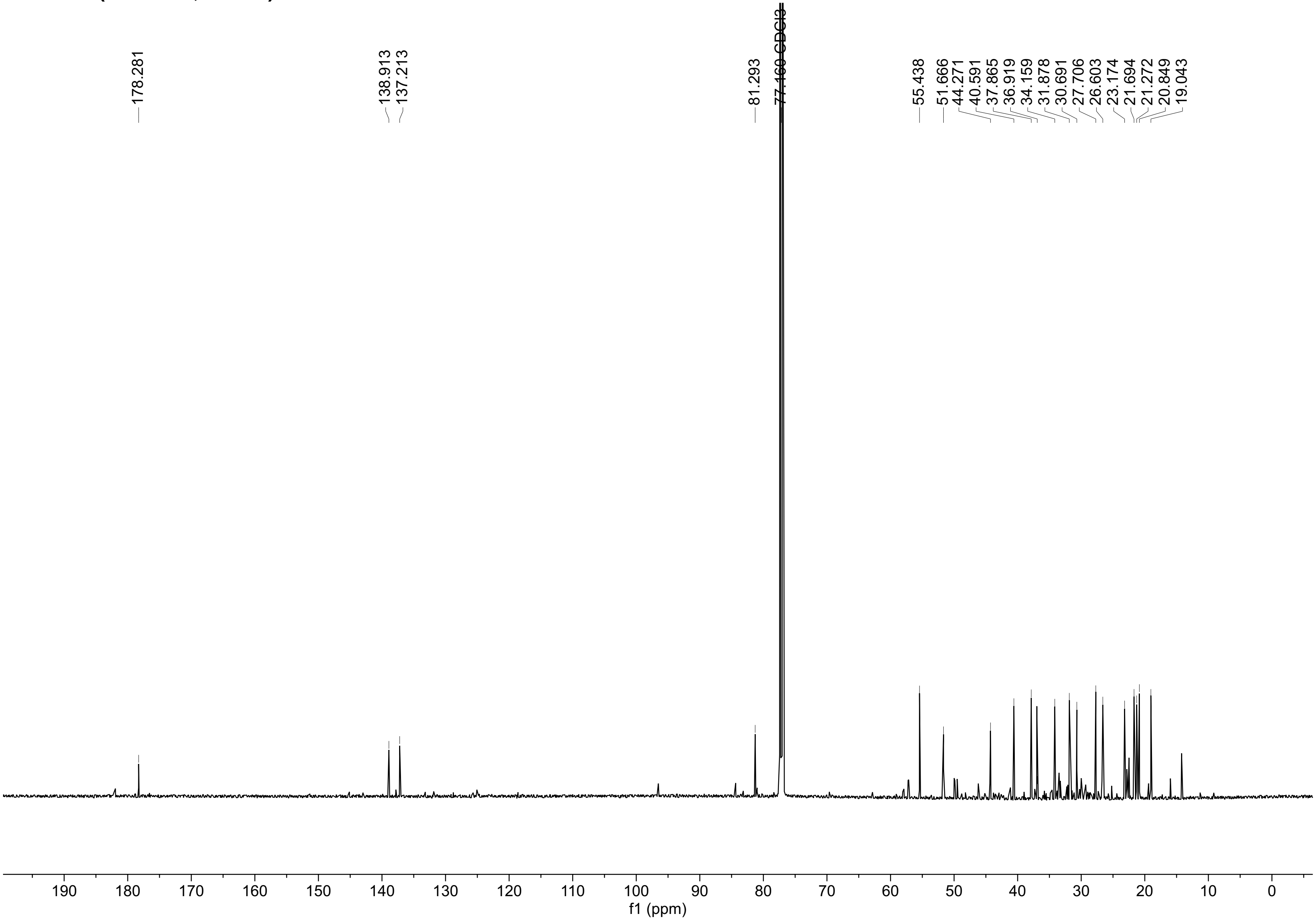

Azorellolide

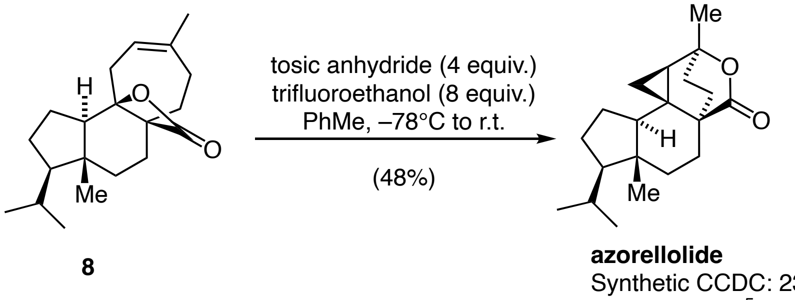

azorellolide
Synthetic CCDC: 2390968
Natural CCDC[5]: 249835

Preparation of "anhydrous" Tosic acid:
To a flame-dried 1 dram vial with stir bar, under argon, tosic anhydride (110 mg, 0.28 mmol, 10 equiv.) and anhydrous toluene (1 mL) were added. After cooling to 0°C, 2,2,2-trifluoroethan-1-ol (56 mg, 48 µL, 0.66 mmol, 20 equiv.) was added dropwise and the reaction was left to stir vigorously for 10 minutes. Upon addition of TFE, a solid precipitated immediately. This solution was left to stir under argon.

8 (10.0 mg, 33 µmol, 1 equiv.) in anhydrous toluene (1 mL) was cooled to –78°C. 200 µL of the above prepared anhydrous tosic acid was added dropwise over 30 seconds. The reaction mixture was left to stir 12 hours at –78 °C, with the bath naturally warming to room temperature. The reaction was then cooled again to –78 °C and 200 µL of the anhydrous tosic acid solution was added dropwise over 30 seconds into the reaction mixture. Once again, the bath was naturally allowed to warm to room temperature and quenched (full conversion of the starting material was not observed) with saturated aqueous $NaHCO_3$ (1 mL). The toluene layer is separated, and the aqueous layer is extracted (3 x 1 mL Et_2O). The organic layer is collected and washed with brine (1 mL). The brine layer is back-extracted (3 x 1 mL Et_2O), dried over anhydrous Na_2SO_4, filtered, and concentrated *in–vacuo*, The reaction is purified by flash-column chromatography (25% Et_2O/Pentanes) to yield **azorellolide** as colorless needles (4.8 mg, 16 µmol) in a 48% yield, and **8** (0.5 mg, 2 µmol, 5% yield) 51% BRSM.

$[\alpha]_D^{23}$ **=** –39.0° (c = 0.41, MeOH).
Reported: $[\alpha]_D^{19.8}$ **=** –64.94° (c = 0.56, $CHCl_3$).

^{1}H NMR (500 MHz, $CDCl_3$): **δ** 2.42 (ddd, *J* = 13.4, 10.8, 4.0 Hz, 1H), 2.27 (td, *J* = 14.5, 4.8 Hz, 1H), 2.00 (ddd, *J* = 13.3, 4.8, 2.6 Hz, 1H), 1.93 (dd, *J* = 12.6, 7.3 Hz, 1H), 1.85 (ddd, *J* = 13.3, 6.8, 3.7 Hz, 2H), 1.73 (ddd, *J* = 13.3, 11.0, 4.8 Hz, 1H), 1.54 – 1.48 (m, 1H), 1.46 (s, 3H), 1.47 – 1.41 (m, 1H), 1.36 – 1.30 (m, 2H), 1.31 – 1.22 (m, 2H), 1.17 – 1.12 (m, 1H), 1.12 – 1.06 (m, 1H), 0.96 – 0.94 (m, 1H), 0.95 (d, *J* = 6.6 Hz 3H), 0.85 (d, *J* = 6.6 Hz, 3H), 0.74 (s, 3H), 0.71 (dd, *J* = 7.9, 3.7 Hz, 1H), 0.61 (t, *J* = 7.4 Hz, 1H), 0.39 (ddd, *J* = 6.5, 3.3, 0.8 Hz, 1H).

^{13}C NMR (151 MHz, $CDCl_3$): δ 176.5, 78.0, 58.3, 45.7, 43.6, 43.0, 35.8, 32.9, 31.1, 28.0, 25.9, 25.4, 24.8, 23.3, 22.8, 21.4, 20.2, 18.8, 11.5, 5.3.

HRMS (ESI): m/z $[M+H]^+$: calcd for $C_{20}H_{31}O_2^+$: 303.2324, found: 303.2326.

R_f (EtOAc/Hex = 20%): 0.40 (*p*-anisaldehyde: dark blue)

m_p: 138.0 – 141.2°C
Reported **m_p**: 146 – 147°C

Comparison of the Original Isolation by **Sosa** [5] vs. Synthetic Standard

**Natural (500 MHz) ^{1}H NMR**	**Synthetic (500 MHz) ^{1}H NMR**	**Δ ppm (Synthetic–Natural)**
2.42 (ddd, *J* = 13.6, 11.3,4.0 Hz)	2.42 (ddd, *J* = 13.4, 10.8, 4.0 Hz)	0
2.27 (td, *J* = 14.5, 5.0 Hz)	2.27 (td, *J* = 14.5, 4.8 Hz)	0
1.99 (ddd, *J* = 13.3, 5.0, 3.0 Hz)	2.00 (ddd, *J* = 13.3, 4.8, 2.6 Hz)	0.01
1.93 (dd, *J* = 12.7, 7.5 Hz)	1.93 (dd, *J* = 12.6, 7.3 Hz)	0
1.85 (ddd, *J* = 13.4, 10.8,4.0 Hz)	1.85 (ddd, *J* = 13.3, 6.8, 3.7 Hz)	0
1.83 (m)	--	
1.72 (ddd, *J* = 13.4, 11.3, 5.0 Hz)	1.73 (ddd, *J* = 13.3, 11.0, 4.8 Hz)	0.01
1.51 (m)	1.54 – 1.48 (m) overlapped with water	0.03
1.43 (s)	1.46 (s)	0.03
1.41 (m)	1.47 – 1.41 (m)	0.06
1.32 (m)	1.36 – 1.30 (m)	0.04
1.28 (m)	1.31 – 1.22 (m)	0.01
1.26 (ddd, *J* = 13.6,11.3, 4.0 Hz)	1.31 – 1.22 (m)	0.05
1.15 (m)	1.17 – 1.12 (m)	0.03
1.08 (m)	1.12 – 1.06 (m)	0.04
0.96 (dddd, *J* = 12.7, 12.0, 10.6, 7.5 Hz)	0.96 – 0.94 (m, overlapped with 0.95 d)	0.02
0.92 (d, *J* = 6.5 Hz)	0.95 d (*J* = 6.6 Hz)	0.03
0.82 (d, *J* = 6.5 Hz)	0.85 d (*J* = 6.6 Hz)	0.03
0.71 (s)	0.74 (s)	0.03
0.69 (dd, *J* = 8.0, 3.5 Hz)	0.71 (dd, *J* = 7.9, 3.7 Hz)	0.02
0.62 (dd, *J* = 8.0, 7.0 Hz)	0.61 (t, *J* = 7.4 Hz)	-0.01
0.39 (ddd, *J* = 7.0, 3.5, 1.0 Hz)	0.39 (ddd, *J* = 6.8, 3.7, 0.8 Hz)	0

^{13}**C NMR** Comparison of Original Isolation by **Sosa*** vs. Synthetic

Original Isolation Natural (125 MHz)	Synthetic (151 MHz)	Δ (Synthetic–Natural)
177.0	176.5	-0.5
78.5	77.98	-0.52
58.8	58.31	-0.49
46.2	45.72	-0.48
44.0	43.56	-0.44
43.4	42.98	-0.42
36.2	35.76	-0.44
33.4	32.94	-0.46
31.6	31.15	-0.45
28.5	27.97	-0.53
26.3	25.85	-0.45
25.9	25.45	-0.45
25.3	24.78	-0.52
23.8	23.26	-0.54
23.3	22.76	-0.54
21.8	21.36	-0.44
20.7	20.24	-0.46
19.3	18.79	-0.51
11.9	11.5	-0.4
5.8	5.3	-0.5

***Note**: There is another isolation of **azorellolide** by *Lima** *et. al.*[6] that contains ^{1}HNMR data, and ^{13}C data that is in accordance with the natural isolation by Sosa[5] and our synthetic **azorellolide**.

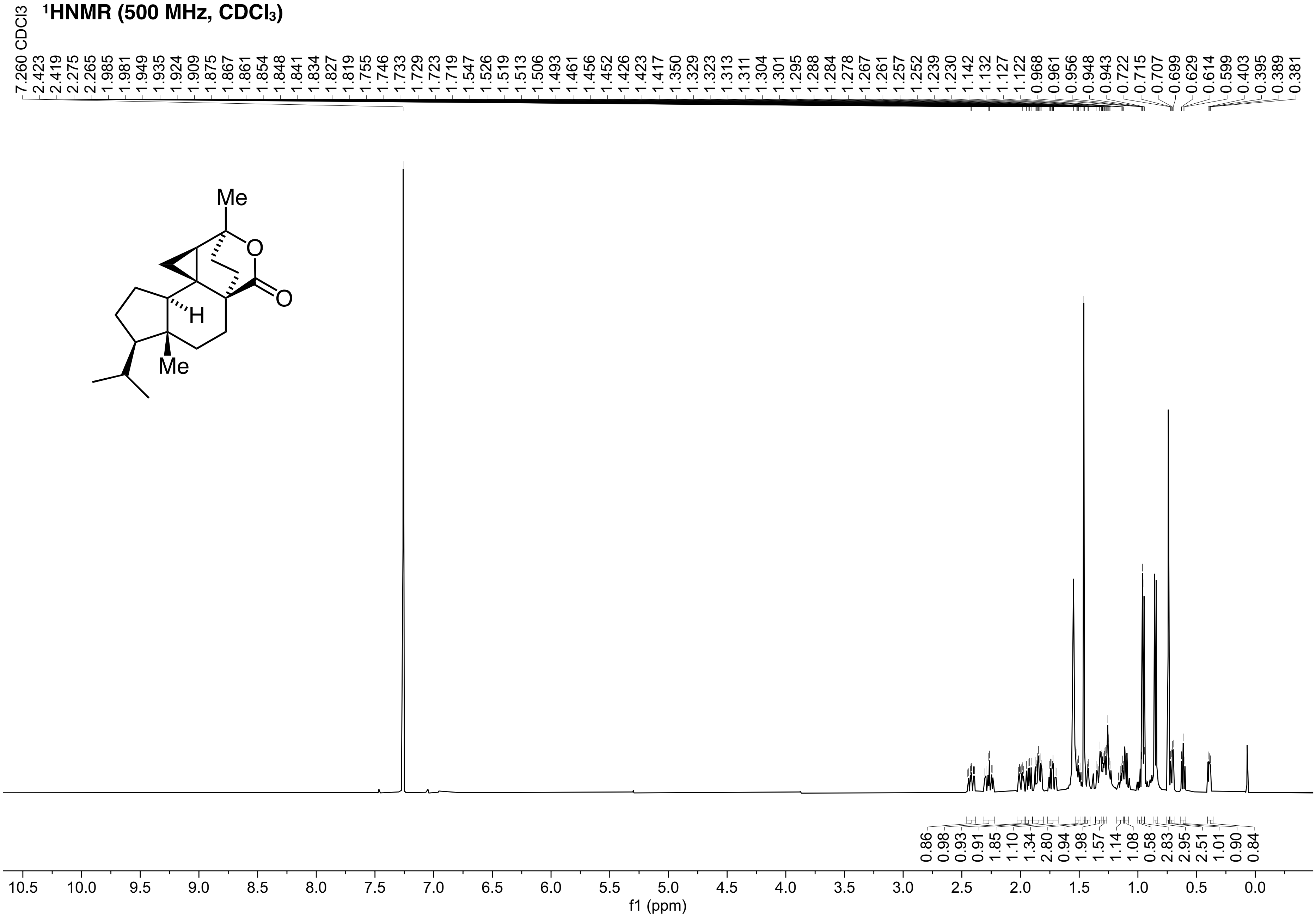

¹HNMR (500 MHz, CDCl₃)
7.260 CDCl3
2.423
2.419
2.275
2.265
1.985
1.981
1.949
1.935
1.924
1.909
1.875
1.867
1.861
1.854
1.848
1.841
1.834
1.827
1.819
1.755
1.746
1.733
1.729
1.723
1.719
1.547
1.526
1.519
1.513
1.506
1.493
1.461
1.456
1.452
1.426
1.423
1.417
1.350
1.329
1.323
1.313
1.311
1.304
1.301
1.295
1.288
1.284
1.278
1.267
1.261
1.257
1.252
1.239
1.230
1.142
1.132
1.127
1.122
0.968
0.961
0.956
0.948
0.943
0.722
0.715
0.707
0.699
0.629
0.614
0.599
0.403
0.395
0.389
0.381
Me
O
O
H
Me
0.86
0.98
0.93
0.91
1.85
1.10
1.34
2.80
0.94
1.98
1.57
1.14
1.08
0.58
2.83
2.95
2.51
1.01
0.90
0.84
10.5
10.0
9.5
9.0
8.5
8.0
7.5
7.0
6.5
6.0
5.5
5.0
4.5
4.0
3.5
3.0
2.5
2.0
1.5
1.0
0.5
0.0
f1 (ppm)

^{13}CNMR (151 MHz, $CDCl_3$)

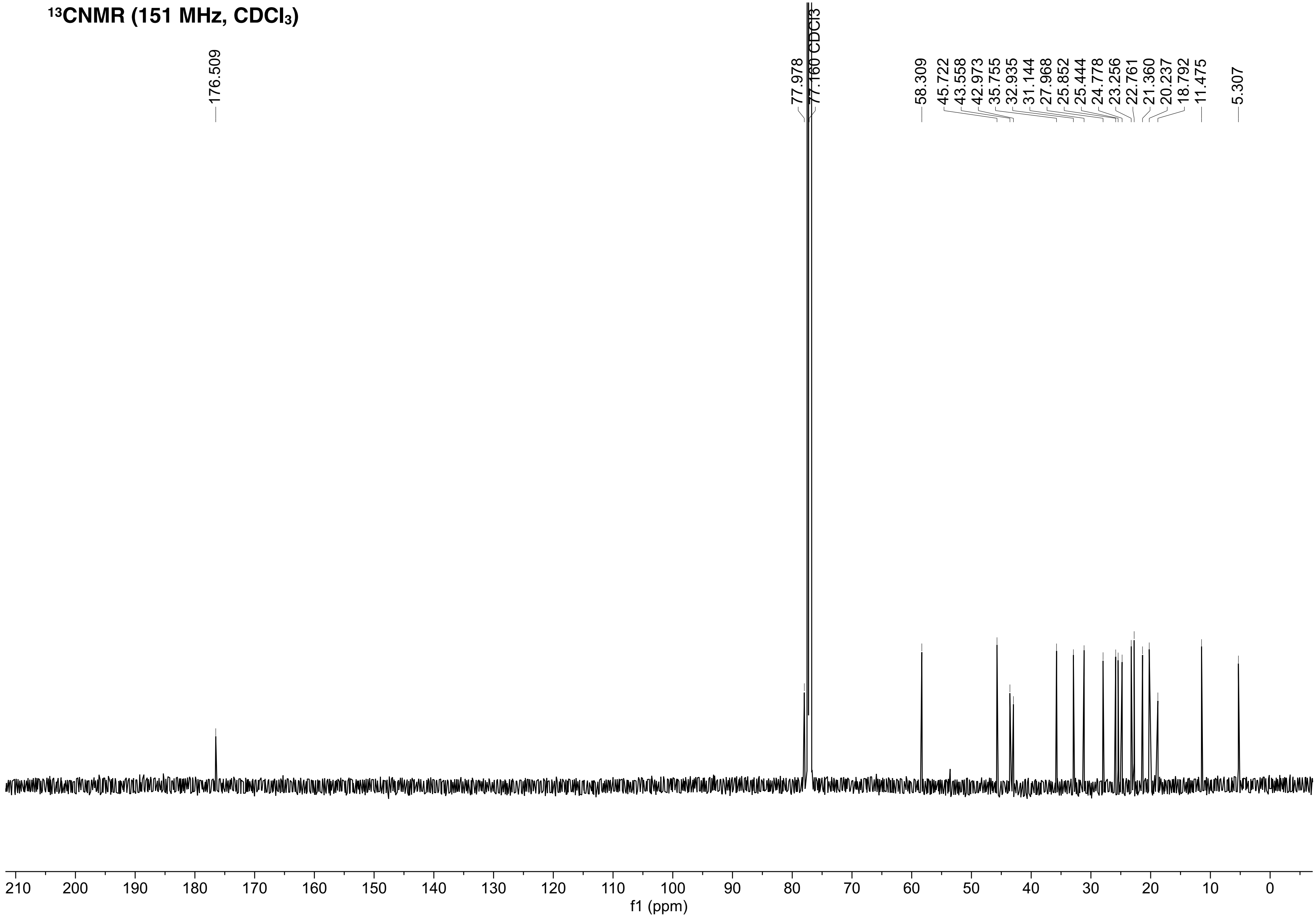

General Procedure for sulfonic-acid promoted rearrangement of **8** to **azorellolide**, **19**, **21**

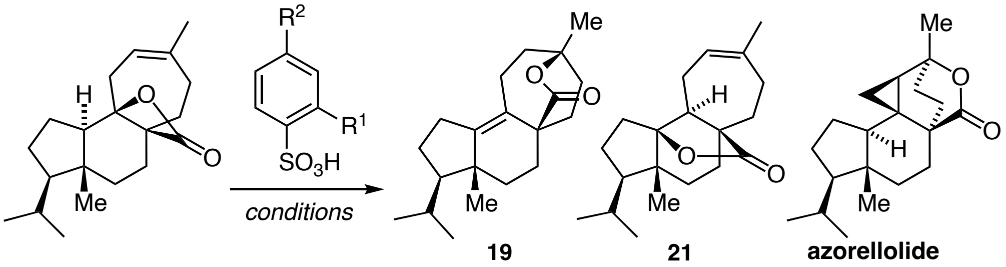

entry	*R*[1]	*R*[2]	*conditions*	*equiv.*	**19 (%)**	**21 (%)**	**azorellolide** (%)
1	H	Cl	PhMe, 0°C to r.t.	5.0	25	50	20
2	NO_2	CF_3	PhMe, 0°C to r.t.	1.0	60	21	14
3	H	H	PhMe, 0°C to 40°C	1.0	42	15	30
4	H	OMe	PhMe, 0°C to 40°C	1.0	54	19	27
5	Br	CF_3	PhMe, 0°C	1.0	35	28	33
6[***a,b***]	Br	CF_3	PhMe, MeCN 0°C	1.0	*trace*	*trace*	39

*reactions performed at 5.0 mg scale. (**a**) acid was predissolved in MeCN (**b**) 5:1 PhMe:MeCN

General Procedure for sulfonic-acid promoted rearrangement of **8** to **azorellolide**

General Procedure A (Entries 1-5)

To a flame–dried 1 dram vial with septa–cap and stir bar was added **8** (5.0 mg, 17 µmol, 1 equiv.), anhydrous PhMe (0.55 mL, 0.03 M) and cooled to 0°C. Sulfonic acid was added (entry *xx* equiv.) and the reaction was left to stir. if minimal, or no reactivity was detected by TLC at 0°C, the reaction was brought to room temperature (entires 1-4). If no reactivity was detected at room temperature, the reaction was brought to 40°C (entries 3-4). Once the starting material was consumed (typically 30 minutes to 8 hours), the reactions were brought to 0°C, and quenched with cold saturated aqueous $NaHCO_3$ (1 mL). The toluene layer is separated, and the aqueous layer is extracted (3 x 1 mL Et_2O). The organic layer is collected and washed with brine (1 mL). The brine layer is back-extracted (3 x 1 mL Et_2O), dried over anhydrous Na_2SO_4, filtered, and concentrated *in–vacuo*, The reaction is purified with flash-column chromatography (25% Et_2O/Pentanes) to give **azorellolide, 19,** and **21** in isolated yields above.

General Procedure B (Entry 6)

To a flame–dried 1 dram vial with septa–cap and stir bar was added **8** (5.0 mg, 17 µmol, 1 equiv.), anhydrous PhMe (0.460 mL) and cooled to 0°C. Sulfonic acid was added (*entry 6, 1.0* equiv.) as a solution in MeCN (91 µL) (total 0.03 M) and the reaction was left to stir. After 15 minutes, the starting material was consumed, and the reaction was quenched with cold saturated aqueous $NaHCO_3$ (1 mL). The toluene layer is separated, and the aqueous layer is extracted (3 x 1 mL Et_2O). The organic layer is collected and washed with brine (1 mL). The brine layer is back-extracted (3 x 1 mL Et_2O), dried over anhydrous Na_2SO_4, filtered, and concentrated *in–vacuo*, The reaction is purified with flash-column chromatography (25% Et_2O/Pentanes) to give **azorellolide** in 39% yield.

X-ray Structure Determination of Compound S2

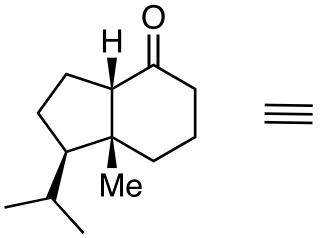

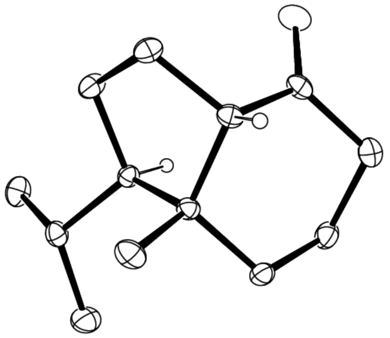

S2
CCDC: 2390969

Compound **S2**, $C_{13}H_{22}O$, crystallizes in the orthorhombic space group $P2_12_12_1$ (systematic absences h00: h=odd, 0k0: k=odd, 00l: l=odd) with a=7.0324(2) Å, b=7.6751(2) Å, c=21.2537(5) Å, α=90°, β=90°, γ=90°, V=1147.16(5) $Å^3$, Z=4, and d_{calc}=1.125 g/cm^3. X-ray intensity data were collected on a Rigaku XtaLAB Synergy-S diffractometer[7] equipped with an HPC area detector (Dectris Pilatus3 R 200 K) and employing confocal multilayer optic-monochromated Mo-Kα radiation (λ=0.71073 Å) at a temperature of 100 K. Preliminary indexing was performed from a series of thirty 0.5° rotation frames with exposures of 0.25 seconds. A total of 2292 frames (20 runs) were collected employing ω scans with a crystal to detector distance of 34.0 mm, rotation widths of 0.5° and exposures of 6 seconds.

Rotation frames were integrated using CrysAlisPro[8], producing a listing of unaveraged F^2 and $\sigma(F^2)$ values. A total of 44920 reflections were measured over the ranges $5.644 \leq 2\theta \leq 56.56°$, $-9 \leq h \leq 9$, $-10 \leq k \leq 10$, $-28 \leq l \leq 28$ yielding 2846 unique reflections (R_{int} = 0.0343). The intensity data were corrected for Lorentz and polarization effects and for absorption using SCALE3 ABSPACK[9] (minimum and maximum transmission 0.62139, 1.00000). The structure was solved by dual methods – SHELXT[10]. Refinement was by full-matrix least squares based on F^2 using SHELXL[11]All reflections were used during refinement. The weighting scheme used was $w=1/[\sigma^2(F_o^2)+(0.0444P)^2+0.1401P]$ where $P=(F_o^2+2F_c^2)/3$. Non-hydrogen atoms were refined

anisotropically and hydrogen atoms were refined using a riding model. Refinement converged to R1=0.0274 and wR2=0.0727 for 2793 observed reflections for which $F > 4\sigma(F)$ and R1=0.0278 and wR2 = 0.0731 and GOF = 1.080 for all 2846 unique, non-zero reflections and 130 variables. The maximum Δ/σ in the final cycle of least squares was 0.001 and the two most prominent peaks in the final difference Fourier were +0.26 and –0.15 e/Å^3.

Table SI1. lists cell information, data collection parameters, and refinement data. Final positional and equivalent isotropic thermal parameters are given in **Tables SI2**. and **SI3**. Anisotropic thermal parameters are in **Table SI4**. **Tables SI5**. and **SI6**. list bond distances and bond angles. **Figure 3**. is an ORTEP representation of **S2** with 50% probability thermal ellipsoids displayed.

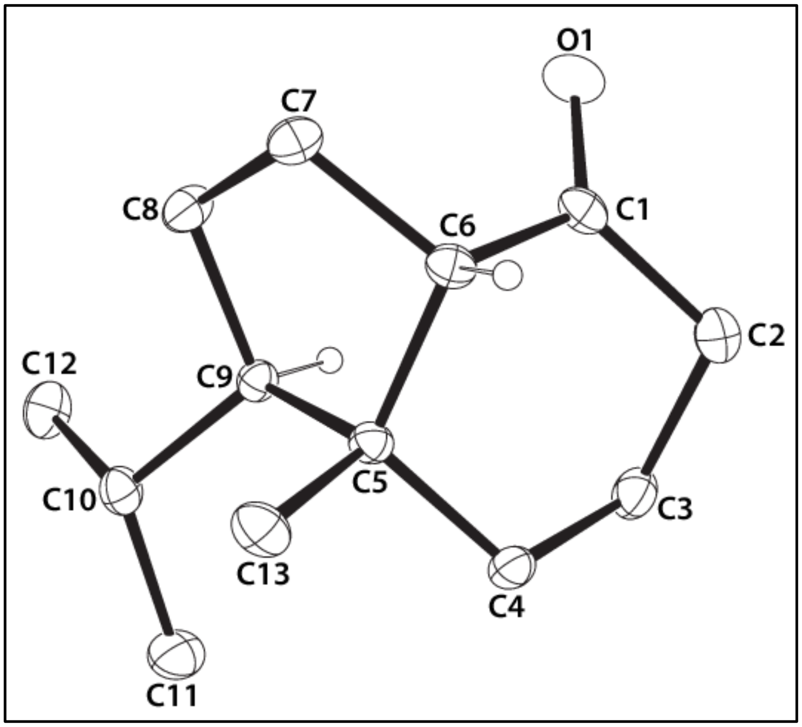

Figure 3. ORTEP drawing of **S2** with 50% thermal ellipsoids.

Table SI1. Summary of Structure Determination of Compound S2

Empirical formula	$C_{13}H_{22}O$
Formula weight	194.30
Diffractometer	Rigaku XtaLAB Synergy-S (Dectris Pilatus3 R 200 K)
Temperature/K	100
Crystal system	orthorhombic
Space group	$P2_12_12_1$
a	7.0324(2) Å
b	7.6751(2) Å
c	21.2537(5) Å
α	90°
β	90°
γ	90°
Volume	1147.16(5) $Å^3$
Z	4
d_{calc}	1.125 g/cm^3
μ	0.068 mm^{-1}
F(000)	432.0
Crystal size, mm	0.39 × 0.37 × 0.15
2θ range for data collection	5.644 - 56.56°
Index ranges	$-9 \le h \le 9$, $-10 \le k \le 10$, $-28 \le l \le 28$
Reflections collected	44920
Independent reflections	2846[R(int) = 0.0343]
Data/restraints/parameters	2846/0/130
Goodness-of-fit on F^2	1.080
Final R indexes [I>=2σ (I)]	$R_1 = 0.0274$, $wR_2 = 0.0727$
Final R indexes [all data]	$R_1 = 0.0278$, $wR_2 = 0.0731$
Largest diff. peak/hole	0.26/-0.15 $eÅ^{-3}$
Flack parameter	–0.1(2)

Table SI2. Refined Positional Parameters for Compound S2

Atom	*x*	*y*	*z*	U(eq)
O1	0.60715(12)	0.83977(12)	0.74902(4)	0.0217(2)
C1	0.44691(16)	0.88286(14)	0.73345(5)	0.0154(2)
C2	0.27993(17)	0.86983(16)	0.77761(5)	0.0196(2)
C3	0.12921(16)	0.74951(15)	0.74833(5)	0.0179(2)
C4	0.07238(15)	0.81430(14)	0.68312(5)	0.0153(2)
C5	0.23892(15)	0.83943(13)	0.63688(5)	0.0123(2)
C6	0.40024(15)	0.94999(14)	0.66762(5)	0.0138(2)
C7	0.56826(15)	0.92991(14)	0.62284(5)	0.0167(2)
C8	0.55087(16)	0.74150(15)	0.59814(5)	0.0170(2)
C9	0.35101(15)	0.67195(13)	0.61788(5)	0.0120(2)
C10	0.26758(16)	0.55143(14)	0.56683(5)	0.0152(2)
C11	0.06668(17)	0.48501(16)	0.58057(6)	0.0235(3)
C12	0.39873(18)	0.39323(15)	0.55769(6)	0.0200(2)
C13	0.16274(17)	0.93534(15)	0.57864(5)	0.0177(2)

Table SI3. Positional Parameters for Hydrogens in Compound S2.

Atom	*x*	*y*	*z*	U(eq)
H2A	0.225147	0.98694	0.784875	0.024
H2B	0.321944	0.822217	0.818607	0.024
H3A	0.180663	0.629789	0.745049	0.021
H3B	0.01565	0.745882	0.775832	0.021
H4A	0.005137	0.926873	0.687812	0.018
H4B	–0.018425	0.730163	0.664575	0.018
H6	0.360902	1.075047	0.669815	0.017
H7A	0.690186	0.947022	0.645293	0.02
H7B	0.56011	1.014825	0.587884	0.02
H8A	0.652076	0.667631	0.616492	0.02
H8B	0.563867	0.739551	0.551762	0.02
H9	0.368431	0.600484	0.656796	0.014
H10	0.263841	0.617808	0.526363	0.018
H11A	0.063825	0.430658	0.622278	0.035
H11B	0.030343	0.39898	0.548685	0.035
H11C	–0.0228	0.582853	0.57953	0.035
H12A	0.527339	0.433397	0.547458	0.03

H12B	0.350271	0.320886	0.523245	0.03
H12C	0.402183	0.324545	0.596545	0.03
H13A	0.121486	1.05267	0.59066	0.027
H13B	0.054661	0.870833	0.561184	0.027
H13C	0.263485	0.943679	0.546923	0.027

Table SI4. Refined Thermal Parameters (U's) for Compound S2

Atom	U_{11}	U_{22}	U_{33}	U_{23}	U_{13}	U_{12}
O1	0.0175(4)	0.0246(4)	0.0229(4)	0.0018(3)	–0.0065(3)	–0.0001(3)
C1	0.0169(5)	0.0126(4)	0.0165(5)	–0.0024(4)	–0.0029(4)	–0.0022(4)
C2	0.0212(5)	0.0238(5)	0.0138(5)	–0.0021(4)	0.0001(4)	–0.0007(5)
C3	0.0178(5)	0.0192(5)	0.0167(5)	–0.0011(4)	0.0050(4)	–0.0019(4)
C4	0.0113(4)	0.0164(5)	0.0183(5)	–0.0037(4)	0.0009(4)	0.0009(4)
C5	0.0114(4)	0.0121(4)	0.0133(4)	–0.0008(4)	–0.0009(4)	0.0008(4)
C6	0.0134(5)	0.0121(4)	0.0157(5)	–0.0002(4)	–0.0013(4)	–0.0016(4)
C7	0.0143(5)	0.0169(5)	0.0189(5)	0.0014(4)	0.0020(4)	–0.0029(4)
C8	0.0135(5)	0.0182(5)	0.0193(5)	–0.0004(4)	0.0037(4)	–0.0002(4)
C9	0.0119(4)	0.0120(4)	0.0122(4)	0.0003(4)	0.0004(4)	0.0010(4)
C10	0.0176(5)	0.0136(5)	0.0145(5)	–0.0027(4)	–0.0002(4)	0.0008(4)
C11	0.0187(5)	0.0218(5)	0.0299(6)	–0.0099(5)	–0.0001(5)	–0.0037(5)
C12	0.0249(6)	0.0143(5)	0.0208(5)	–0.0026(4)	0.0037(4)	0.0024(4)
C13	0.0188(5)	0.0164(5)	0.0179(5)	0.0017(4)	–0.0053(4)	0.0031(4)

Table SI5. Bond Distances in Compound S2, Å

O1-C1	1.2202(14)	C1-C2	1.5066(16)	C1-C6	1.5267(15)
C2-C3	1.5373(16)	C3-C4	1.5258(16)	C4-C5	1.5410(15)
C5-C6	1.5600(15)	C5-C9	1.5610(14)	C5-C13	1.5367(14)
C6-C7	1.5250(15)	C7-C8	1.5433(16)	C8-C9	1.5610(15)
C9-C10	1.5418(14)	C10-C11	1.5300(16)	C10-C12	1.5370(15)

Table SI6. Bond Angles in Compound S2, °

O1-C1-C2	122.20(10)	O1-C1-C6	122.58(10)	C2-C1-C6	115.20(9)
C1-C2-C3	108.97(9)	C4-C3-C2	110.64(9)	C3-C4-C5	114.90(9)
C4-C5-C6	110.72(8)	C4-C5-C9	116.47(9)	C6-C5-C9	100.90(8)
C13-C5-C4	107.98(9)	C13-C5-C6	109.29(9)	C13-C5-C9	111.23(9)
C1-C6-C5	110.88(9)	C7-C6-C1	111.79(9)	C7-C6-C5	104.31(8)
C6-C7-C8	104.22(9)	C7-C8-C9	107.48(9)	C8-C9-C5	104.04(8)
C10-C9-C5	118.95(9)	C10-C9-C8	111.02(9)	C11-C10-C9	114.64(9)
C11-C10-C12	108.36(9)	C12-C10-C9	109.55(9)		

This report has been created with Olex2[12], compiled on 2022.04.07 svn.rca3783a0 for OlexSys.

X-ray Structure Determination of Compound 16

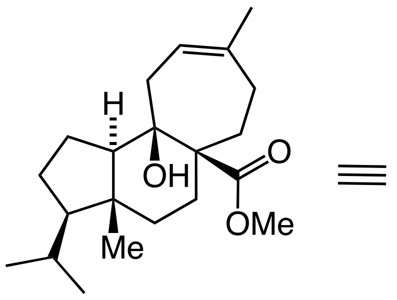

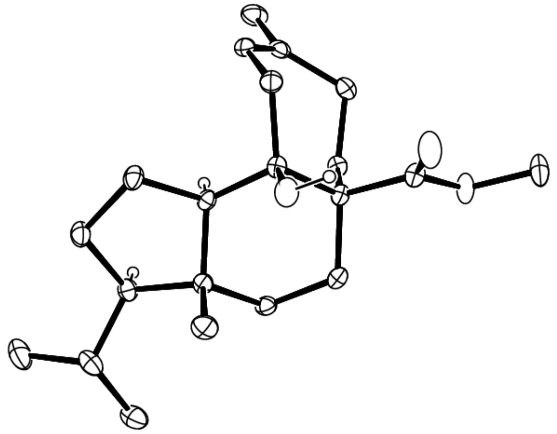

16
CCDC: 2390964

Compound **16**, $C_{21}H_{34}O_3$, crystallizes in the orthorhombic space group $P2_12_12_1$ (systematic absences h00: h=odd, 0k0: k=odd, 00l: l=odd) with a=9.0753(2) , b=11.7407(3) Å, c=17.8204(4) Å, α=90°, β=90°, γ=90°, V=1898.77(8) Å^3, Z=4, and d_{calc}=1.170 g/cm^3. X-ray intensity data were collected on a Rigaku XtaLAB Synergy-S diffractometer[7] equipped with an HPC area detector (HyPix-6000HE) and employing confocal multilayer optic-monochromated Mo-Kα radiation (λ=0.71073 Å) at a temperature of 100 K. Preliminary indexing was performed from a series of thirty 0.5° rotation frames with exposures of 10 seconds. A total of 1084 frames (14 runs) were collected employing ω scans with a crystal to detector distance of 34.0 mm, rotation widths of 0.5° and exposures of 60 seconds.

Rotation frames were integrated using CrysAlisPro[8], producing a listing of unaveraged F^2 and $\sigma(F^2)$ values. A total of 33411 reflections were measured over the ranges $4.154 \leq 2\theta \leq 56.558°$, $-11 \leq h \leq 12$, $-15 \leq k \leq 15$, $-23 \leq l \leq 23$ yielding 4708 unique reflections (R_{int} = 0.0379). The intensity data were corrected for Lorentz and polarization effects and for absorption using SCALE3 ABSPACK[9] (minimum and maximum transmission 0.75199, 1.00000). The structure was solved by dual methods – SHELXT.[10] Refinement was by full-matrix least squares based on F^2 using SHELXL.[11] All reflections were used during refinement. The weighting scheme used was

$w=1/[\sigma^2(F_o^2)+(0.0515P)^2+0.2586P]$ where $P=(F_o^2+2F_c^2)/3$. Non-hydrogen atoms were refined anisotropically and hydrogen atoms were refined using a riding model. Refinement converged to R1=0.0333 and wR2=0.0845 for 4406 observed reflections for which $F > 4\sigma(F)$ and R1=0.0364 and wR2=0.0859 and GOF =1.036 for all 4708 unique, non-zero reflections and 223 variables. The maximum Δ/σ in the final cycle of least squares was 0.002 and the two most prominent peaks in the final difference Fourier were +0.28 and –0.15 e/Å^3.

Table **SI7**. lists cell information, data collection parameters, and refinement data. Final positional and equivalent isotropic thermal parameters are given in **Tables SI8**. And **SI9**. Anisotropic thermal parameters are in **Table SI10**. **Tables SI11** and **SI12**. list bond distances and bond angles. **Figure 4**. is an ORTEP representation of **16** with 50% probability thermal ellipsoids displayed.

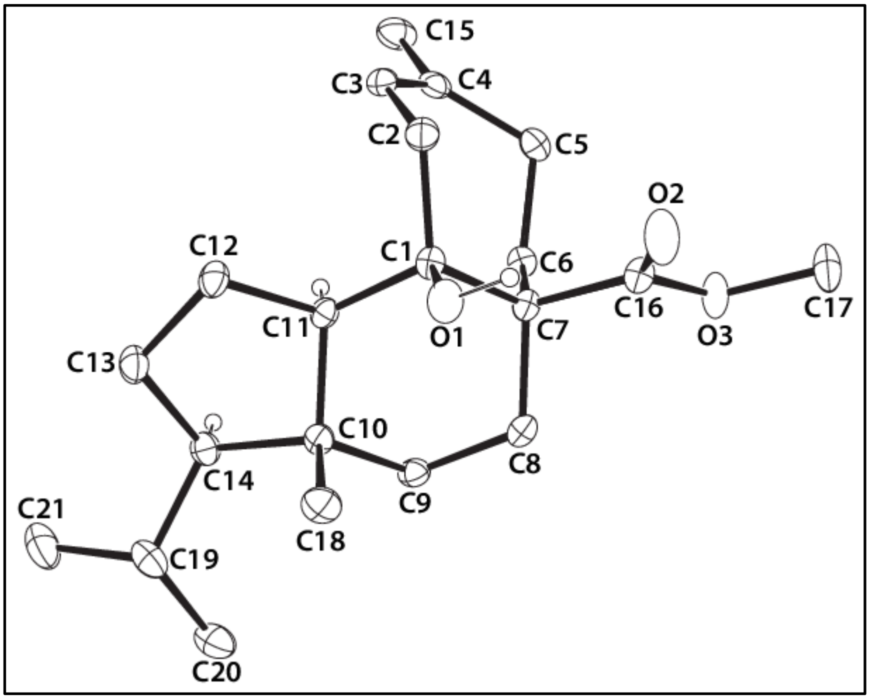

Figure 4. ORTEP drawing of **16** with 50% thermal ellipsoids.

Table SI7. Summary of Structure Determination of Compound 16

Empirical formula	$C_{21}H_{34}O_3$
Formula weight	334.48
Diffractometer	Rigaku XtaLAB Synergy-S (HyPix-6000HE)
Temperature/K	100
Crystal system	orthorhombic
Space group	$P2_12_12_1$
a	9.0753(2) Å
b	11.7407(3) Å
c	17.8204(4) Å
α	90°
β	90°
γ	90°
Volume	1898.77(8) $Å^3$
Z	4
d_{calc}	1.170 g/cm^3
μ	0.076 mm^{-1}
F(000)	736.0
Crystal size, mm	0.18 × 0.08 × 0.06
2θ range for data collection	4.154 - 56.558°
Index ranges	$-11 \le h \le 12$, $-15 \le k \le 15$, $-23 \le l \le 23$
Reflections collected	33411
Independent reflections	4708[R(int) = 0.0379]
Data/restraints/parameters	4708/0/223
Goodness-of-fit on F^2	1.036
Final R indexes [I>=2σ (I)]	R_1 = 0.0333, wR_2 = 0.0845
Final R indexes [all data]	R_1 = 0.0364, wR_2 = 0.0859
Largest diff. peak/hole	0.28/–0.15 e$Å^{-3}$
Flack parameter	0.1(3)

Table SI8. Refined Positional Parameters for Compound 16

Atom	*x*	*y*	*z*	U(eq)
O1	0.18534(12)	0.38479(9)	0.26658(6)	0.0189(2)
O2	–0.05028(13)	0.45834(10)	0.18764(8)	0.0277(3)
O3	–0.08424(12)	0.64622(10)	0.18792(7)	0.0222(3)
C1	0.25466(16)	0.47624(12)	0.22671(8)	0.0136(3)
C2	0.28940(17)	0.43152(13)	0.14684(8)	0.0175(3)
C3	0.39355(17)	0.50028(13)	0.09901(8)	0.0177(3)
C4	0.36347(17)	0.60361(14)	0.07288(8)	0.0177(3)
C5	0.22189(17)	0.66298(13)	0.09429(8)	0.0173(3)
C6	0.20894(17)	0.68486(12)	0.17895(8)	0.0145(3)
C7	0.15200(16)	0.58429(12)	0.22719(8)	0.0138(3)
C8	0.13305(16)	0.62745(13)	0.30942(8)	0.0163(3)
C9	0.27953(17)	0.65255(12)	0.34935(8)	0.0158(3)
C10	0.37943(16)	0.54701(12)	0.35111(8)	0.0143(3)
C11	0.39618(16)	0.50518(12)	0.26889(8)	0.0132(3)
C12	0.51605(17)	0.41391(13)	0.27285(9)	0.0176(3)
C13	0.62175(18)	0.46068(14)	0.33374(9)	0.0192(3)
C14	0.54524(17)	0.56596(13)	0.36994(8)	0.0159(3)
C15	0.46760(19)	0.66815(16)	0.02303(9)	0.0240(3)
C16	–0.00272(17)	0.55313(13)	0.19888(9)	0.0173(3)
C17	–0.23150(18)	0.62840(15)	0.15943(11)	0.0257(4)
C18	0.31474(19)	0.45861(14)	0.40542(9)	0.0207(3)
C19	0.59230(19)	0.58071(14)	0.45243(9)	0.0216(3)
C20	0.5174(2)	0.68024(16)	0.49207(9)	0.0259(4)
C21	0.7604(2)	0.59601(17)	0.45685(11)	0.0301(4)

Table SI9. Positional Parameters for Hydrogens in Compound 16.

Atom	*x*	*y*	*z*	U(eq)
H1	0.103705	0.369914	0.246643	0.028
H2A	0.330672	0.353832	0.151813	0.021
H2B	0.195045	0.424537	0.119333	0.021
H3	0.486344	0.467717	0.086821	0.021
H5A	0.137454	0.615793	0.077843	0.021
H5B	0.216198	0.736652	0.067412	0.021
H6A	0.142119	0.750489	0.186594	0.017
H6B	0.307244	0.707231	0.197996	0.017
H8A	0.072777	0.697731	0.308886	0.02
H8B	0.078497	0.569273	0.338496	0.02
H9A	0.330979	0.715248	0.323014	0.019
H9B	0.259381	0.677763	0.401362	0.019
H11	0.441179	0.570308	0.240917	0.016
H12A	0.567025	0.405587	0.22404	0.021
H12B	0.474469	0.339329	0.287802	0.021
H13A	0.71682	0.483224	0.310903	0.023
H13B	0.640989	0.401715	0.372221	0.023
H14	0.578692	0.635056	0.341903	0.019
H15A	0.418271	0.686516	–0.024368	0.036
H15B	0.554725	0.621301	0.012909	0.036
H15C	0.497727	0.738753	0.048039	0.036
H17A	–0.226163	0.592482	0.109859	0.038
H17B	–0.282171	0.70184	0.155247	0.038
H17C	–0.286049	0.578801	0.193843	0.038
H18A	0.31517	0.489536	0.456493	0.031
H18B	0.374421	0.389086	0.403821	0.031
H18C	0.213341	0.440786	0.390585	0.031
H19	0.566101	0.509332	0.479992	0.026
H20A	0.537588	0.750848	0.464604	0.039
H20B	0.555591	0.686732	0.54332	0.039
H20C	0.410782	0.667083	0.493736	0.039
H21A	0.809036	0.527902	0.436942	0.045
H21B	0.789608	0.607313	0.509257	0.045
H21C	0.789511	0.662554	0.427159	0.045

Table SI10. Refined Thermal Parameters (U's) for Compound 16

Atom	U_{11}	U_{22}	U_{33}	U_{23}	U_{13}	U_{12}
O1	0.0165(5)	0.0138(5)	0.0262(6)	0.0041(4)	0.0001(4)	–0.0042(4)
O2	0.0180(6)	0.0180(6)	0.0471(8)	–0.0003(5)	–0.0070(5)	–0.0028(5)
O3	0.0127(5)	0.0182(5)	0.0358(6)	–0.0011(5)	–0.0055(5)	0.0023(4)
C1	0.0130(6)	0.0102(6)	0.0174(7)	0.0006(5)	0.0000(5)	–0.0002(5)
C2	0.0177(7)	0.0149(7)	0.0200(7)	–0.0040(6)	–0.0014(6)	0.0004(5)
C3	0.0148(7)	0.0221(7)	0.0161(7)	–0.0066(6)	0.0002(5)	0.0007(6)
C4	0.0165(7)	0.0237(8)	0.0130(6)	–0.0045(6)	–0.0017(5)	–0.0044(6)
C5	0.0176(7)	0.0182(7)	0.0161(7)	0.0024(6)	–0.0030(6)	–0.0015(6)
C6	0.0152(7)	0.0116(6)	0.0166(7)	0.0004(5)	–0.0015(5)	–0.0003(5)
C7	0.0119(6)	0.0123(6)	0.0172(6)	–0.0004(5)	0.0004(5)	0.0003(5)
C8	0.0140(7)	0.0167(7)	0.0182(7)	–0.0004(5)	0.0028(5)	0.0022(6)
C9	0.0185(7)	0.0147(6)	0.0142(6)	–0.0011(5)	0.0013(5)	0.0027(5)
C10	0.0149(7)	0.0133(6)	0.0145(6)	0.0021(5)	0.0004(5)	0.0005(5)
C11	0.0129(7)	0.0116(6)	0.0151(6)	0.0010(5)	0.0012(5)	0.0012(5)
C12	0.0159(7)	0.0145(7)	0.0225(7)	–0.0012(6)	–0.0006(6)	0.0037(6)
C13	0.0164(7)	0.0186(7)	0.0227(7)	0.0003(6)	–0.0032(6)	0.0039(6)
C14	0.0157(7)	0.0146(7)	0.0173(7)	0.0018(5)	–0.0014(5)	0.0000(6)
C15	0.0244(8)	0.0290(9)	0.0186(7)	–0.0028(6)	0.0017(6)	–0.0089(7)
C16	0.0139(7)	0.0173(7)	0.0208(7)	0.0001(6)	0.0007(5)	0.0009(6)
C17	0.0138(7)	0.0260(8)	0.0372(9)	–0.0005(7)	–0.0075(7)	0.0018(6)
C18	0.0245(8)	0.0203(7)	0.0172(7)	0.0064(6)	0.0022(6)	–0.0001(6)
C19	0.0261(8)	0.0192(8)	0.0194(7)	0.0024(6)	–0.0074(6)	–0.0001(7)
C20	0.0319(9)	0.0269(8)	0.0190(8)	–0.0026(6)	–0.0046(7)	–0.0006(7)
C21	0.0265(9)	0.0318(9)	0.0320(9)	–0.0015(7)	–0.0136(7)	0.0017(8)

Table SI11. Bond Distances in Compound 16, Å

O1-C1	1.4329(17)	O2-C16	1.2104(19)	O3-C16	1.3342(18)
O3-C17	1.4448(19)	C1-C2	1.550(2)	C1-C7	1.5740(19)
C1-C11	1.526(2)	C2-C3	1.507(2)	C3-C4	1.328(2)
C4-C5	1.511(2)	C4-C15	1.502(2)	C5-C6	1.535(2)
C6-C7	1.549(2)	C7-C8	1.560(2)	C7-C16	1.536(2)
C8-C9	1.536(2)	C9-C10	1.536(2)	C10-C11	1.5528(19)
C10-C14	1.558(2)	C10-C18	1.536(2)	C11-C12	1.529(2)

C12-C13 1.549(2)	C13-C14 1.558(2)	C14-C19 1.541(2)
C19-C20 1.525(2)	C19-C21 1.538(2)	

Table SI12. Bond Angles in Compound 16, °

C16-O3-C17	116.47(13)	O1-C1-C2	106.91(11)	O1-C1-C7	109.96(12)
O1-C1-C11	106.98(11)	C2-C1-C7	113.48(12)	C11-C1-C2	110.89(12)
C11-C1-C7	108.42(11)	C3-C2-C1	117.75(12)	C4-C3-C2	123.97(14)
C3-C4-C5	120.52(14)	C3-C4-C15	122.61(15)	C15-C4-C5	116.85(14)
C4-C5-C6	112.99(12)	C5-C6-C7	116.32(12)	C6-C7-C1	114.45(12)
C6-C7-C8	108.09(11)	C8-C7-C1	109.40(12)	C16-C7-C1	110.32(11)
C16-C7-C6	107.71(12)	C16-C7-C8	106.56(11)	C9-C8-C7	113.70(12)
C10-C9-C8	111.44(12)	C9-C10-C11	107.08(11)	C9-C10-C14	117.35(12)
C11-C10-C14	98.85(11)	C18-C10-C9	109.42(12)	C18-C10-C11	114.72(12)
C18-C10-C14	109.27(12)	C1-C11-C10	116.92(12)	C1-C11-C12	117.74(12)
C12-C11-C10	104.35(11)	C11-C12-C13	102.98(12)	C12-C13-C14	107.19(12)
C13-C14-C10	103.18(12)	C19-C14-C10	119.30(13)	C19-C14-C13	111.16(13)
O2-C16-O3	122.09(14)	O2-C16-C7	126.82(14)	O3-C16-C7	111.09(13)
C20-C19-C14	113.85(13)	C20-C19-C21	109.21(15)	C21-C19-C14	109.69(14)

This report has been created with Olex2[12], compiled on 2022.04.07 svn.rca3783a0 for OlexSys.

X-ray Structure Determination of Compound 18

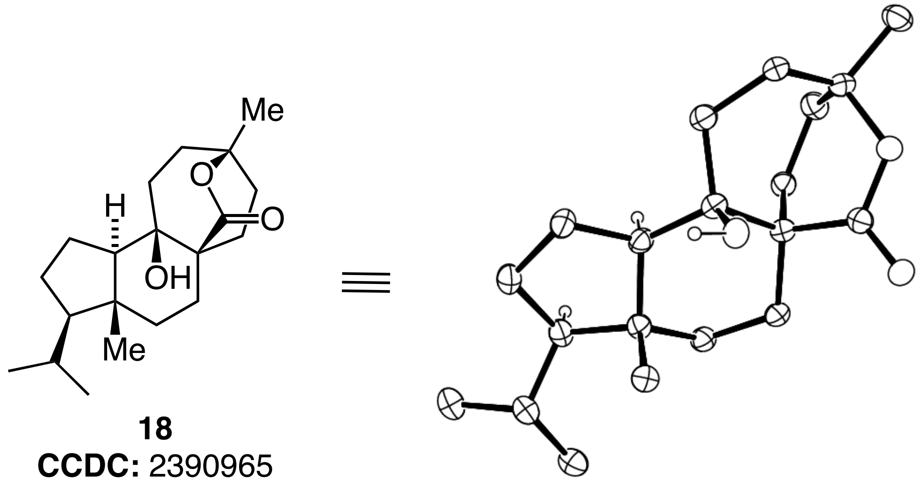

18
CCDC: 2390965

Compound **18**, $C_{20}H_{32}O_3$, crystallizes in the monoclinic space group $P2_1$ (systematic absences 0k0: k=odd) with a=7.52910(10) Å, b=9.9848(2) Å, c=11.5607(2) Å, α=90°, β=102.161(2)°, γ=90°, V=849.59(3)Å^3, Z=2, and d_{calc}=1.253 g/cm^3. X-ray intensity data were collected on a Rigaku XtaLAB Synergy-S diffractometer[7] equipped with an HPC area detector (HyPix-6000HE) and employing confocal multilayer optic-monochromated Cu-Kα radiation (λ=1.54184 Å) at a temperature of 100 K. Preliminary indexing was performed from a series of sixty 0.5° rotation frames with exposures of 0.25 seconds for θ = ±47.554° and 1 second for θ = 113.25°. A total of 4856 frames (42 runs) were collected employing ω scans with a crystal to detector distance of 34.0 mm, rotation widths of 0.5° and exposures of 1 second.

Rotation frames were integrated using CrysAlisPro[8] producing a listing of unaveraged F^2 and $\sigma(F^2)$ values. A total of 13890 reflections were measured over the ranges 7.822 ≤ 2θ ≤ 148.652°, –9 ≤ h ≤ 7, –12 ≤ k ≤ 12, –14 ≤ l ≤ 14 yielding 3332 unique reflections (R_{int} = 0.0428). The intensity data were corrected for Lorentz and polarization effects and for absorption using SCALE3 ABSPACK[9] (minimum and maximum transmission 0.69227, 1.00000). The structure was solved by dual space methods – SHELXT[10]. Refinement was by full-matrix least squares based

on F^2 using SHELXL[11]All reflections were used during refinement. The weighting scheme used was $w=1/[\sigma^2(F_o^2)+(0.0486P)^2+0.2200P]$ where $P=(F_o^2+2F_c^2)/3$. Non-hydrogen atoms were refined anisotropically and hydrogen atoms were refined using a riding model. Refinement converged to R1=0.0368 and wR2=0.0932 for 3119 observed reflections for which $F > 4\sigma(F)$ and R1=0.0401 and wR2=0.0962 and GOF =1.077 for all 3332 unique, non-zero reflections and 213 variables. The maximum Δ/σ in the final cycle of least squares was 0.000 and the two most prominent peaks in the final difference Fourier were +0.17 and –0.21 e/Å^3.

Table **SI13**. lists cell information, data collection parameters, and refinement data. Final positional and equivalent isotropic thermal parameters are given in **Tables SI14**. and **SI15**. Anisotropic thermal parameters are in **Table SI16**. **Tables SI17**. and **SI18.** list bond distances and bond angles. **Figure 5**. is an ORTEP representation of **18** with 50% probability thermal ellipsoids displayed.

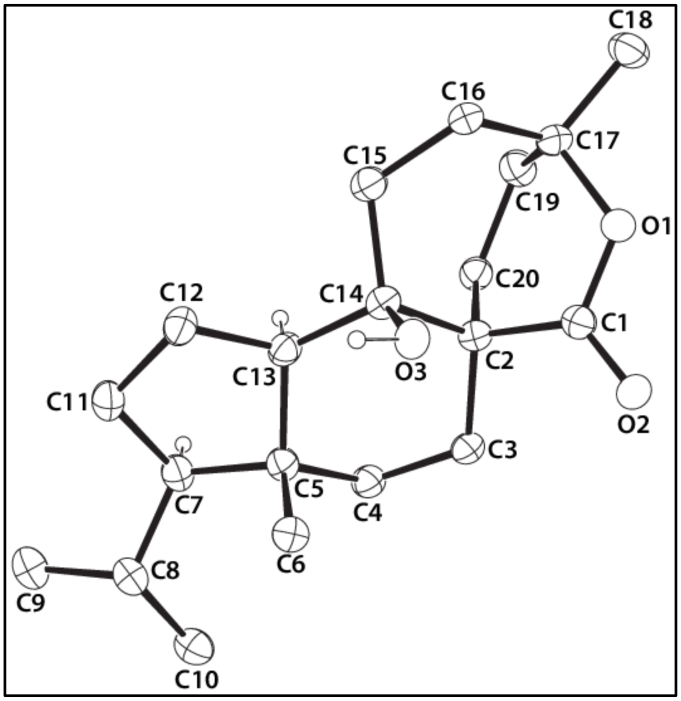

Figure 5. ORTEP drawing of **18** with 50% thermal ellipsoids.

Table SI13. Summary of Structure Determination of Compound 18

Empirical formula	$C_{20}H_{32}O_3$
Formula weight	320.45
Diffractometer	Rigaku XtaLAB Synergy-S (HyPix-6000HE)
Temperature/K	100(2)
Crystal system	monoclinic
Space group	$P2_1$
a	7.52910(10) Å
b	9.9848(2) Å
c	11.5607(2) Å
α	90°
β	102.161(2)°
γ	90°
Volume	849.59(3) $Å^3$
Z	2
d_{calc}	1.253 g/cm^3
μ	0.643 mm^{-1}
F(000)	352.0
Crystal size, mm	0.17 × 0.05 × 0.03
2θ range for data collection	7.822 - 148.652°
Index ranges	$-9 \le h \le 7$, $-12 \le k \le 12$, $-14 \le l \le 14$
Reflections collected	13890
Independent reflections	3332[R(int) = 0.0428]
Data/restraints/parameters	3332/1/213
Goodness-of-fit on F^2	1.077
Final R indexes [I>=2σ (I)]	R_1 = 0.0368, wR_2 = 0.0932
Final R indexes [all data]	R_1 = 0.0401, wR_2 = 0.0962
Largest diff. peak/hole	0.17/–0.21 $eÅ^{-3}$
Flack parameter	0.22(15)

Table SI14. Refined Positional Parameters for Compound 18

Atom	*x*	*y*	*z*	U(eq)
O1	0.3590(2)	0.11514(18)	0.63899(15)	0.0225(4)
O2	0.6338(2)	0.15267(17)	0.61416(16)	0.0232(4)
O3	0.4392(2)	0.42534(18)	0.55222(15)	0.0225(4)
C1	0.5106(3)	0.1912(3)	0.6573(2)	0.0206(5)
C2	0.5167(3)	0.3131(2)	0.7373(2)	0.0206(5)
C3	0.7205(3)	0.3522(3)	0.7739(2)	0.0225(5)
C4	0.7609(3)	0.4851(3)	0.8386(2)	0.0222(5)
C5	0.6656(3)	0.6002(3)	0.7623(2)	0.0210(5)
C6	0.7435(3)	0.6209(3)	0.6512(2)	0.0240(5)
C7	0.6597(3)	0.7356(3)	0.8266(2)	0.0233(5)
C8	0.8250(3)	0.8293(3)	0.8399(2)	0.0257(5)
C9	0.7922(4)	0.9586(3)	0.9038(3)	0.0311(6)
C10	1.0036(4)	0.7664(3)	0.9042(2)	0.0291(6)
C11	0.4835(3)	0.8048(3)	0.7557(2)	0.0269(6)
C12	0.3666(3)	0.6938(3)	0.6839(2)	0.0245(5)
C13	0.4615(3)	0.5643(3)	0.7340(2)	0.0213(5)
C14	0.4031(3)	0.4326(3)	0.6686(2)	0.0213(5)
C15	0.1977(3)	0.4157(3)	0.6610(2)	0.0234(5)
C16	0.1167(3)	0.2817(3)	0.6133(2)	0.0219(5)
C17	0.2013(3)	0.1597(3)	0.6846(2)	0.0233(5)
C18	0.0734(4)	0.0411(3)	0.6605(3)	0.0279(6)
C19	0.2644(3)	0.1865(3)	0.8170(2)	0.0257(5)
C20	0.4419(3)	0.2715(3)	0.8471(2)	0.0222(5)

Table SI15. Positional Parameters for Hydrogens in Compound 18.

Atom	*x*	*y*	*z*	U(eq)
H3	0.408188	0.49753	0.516331	0.034
H3A	0.785203	0.280525	0.825216	0.027
H3B	0.771426	0.355374	0.701676	0.027
H4A	0.893662	0.500994	0.857295	0.027
H4B	0.71857	0.48172	0.91402	0.027
H6A	0.728227	0.53865	0.603922	0.036
H6B	0.873038	0.642772	0.674541	0.036

H6C	0.679072	0.694565	0.604203	0.036
H7	0.640115	0.715773	0.907761	0.028
H8	0.839201	0.854347	0.75856	0.031
H9A	0.678752	1.000318	0.862257	0.047
H9B	0.893449	1.020576	0.90486	0.047
H9C	0.783325	0.937642	0.985231	0.047
H10A	0.991108	0.735527	0.982593	0.044
H10B	1.100945	0.833216	0.913081	0.044
H10C	1.033377	0.690254	0.858356	0.044
H11A	0.416366	0.848078	0.810533	0.032
H11B	0.514941	0.873911	0.701968	0.032
H12A	0.240241	0.696868	0.695706	0.029
H12B	0.365098	0.70253	0.598362	0.029
H13	0.429088	0.553171	0.813042	0.026
H15A	0.173464	0.428622	0.741191	0.028
H15B	0.1334	0.487827	0.61004	0.028
H16A	0.13162	0.271685	0.530674	0.026
H16B	–0.01525	0.28284	0.611836	0.026
H18A	0.04022	0.024671	0.57507	0.042
H18B	–0.036469	0.060227	0.690389	0.042
H18C	0.133583	–0.038452	0.700383	0.042
H19A	0.28537	0.099825	0.859269	0.031
H19B	0.166857	0.234075	0.845817	0.031
H20A	0.417829	0.353281	0.889632	0.027
H20B	0.536316	0.219239	0.901119	0.027

Table SI16. Refined Thermal Parameters (U's) for Compound 18.

Atom	U_{11}	U_{22}	U_{33}	U_{23}	U_{13}	U_{12}
O1	0.0195(8)	0.0213(9)	0.0272(9)	–0.0019(7)	0.0060(6)	–0.0011(7)
O2	0.0197(8)	0.0223(9)	0.0277(9)	–0.0017(7)	0.0055(7)	0.0020(7)
O3	0.0251(8)	0.0204(9)	0.0220(9)	0.0011(7)	0.0048(7)	0.0025(7)
C1	0.0203(11)	0.0205(12)	0.0205(11)	0.0018(10)	0.0032(9)	–0.0004(9)
C2	0.0173(10)	0.0216(13)	0.0227(12)	–0.0003(10)	0.0037(9)	0.0004(9)
C3	0.0168(11)	0.0217(12)	0.0283(13)	0.0017(10)	0.0031(9)	0.0026(9)
C4	0.0197(11)	0.0228(13)	0.0237(12)	0.0012(10)	0.0037(9)	0.0010(9)
C5	0.0207(11)	0.0193(12)	0.0227(12)	–0.0013(10)	0.0037(9)	–0.0004(9)
C6	0.0222(11)	0.0227(13)	0.0276(13)	0.0006(11)	0.0060(9)	–0.0004(10)

C7	0.0268(12)	0.0190(13)	0.0245(12)	0.0005(10)	0.0064(10)	–0.0006(10)
C8	0.0282(13)	0.0214(13)	0.0273(13)	0.0005(10)	0.0055(10)	–0.0033(10)
C9	0.0369(14)	0.0231(14)	0.0330(15)	–0.0017(11)	0.0067(12)	–0.0038(11)
C10	0.0273(12)	0.0289(14)	0.0302(13)	–0.0002(11)	0.0043(10)	–0.0049(11)
C11	0.0284(12)	0.0191(12)	0.0326(14)	0.0016(10)	0.0048(10)	0.0022(10)
C12	0.0245(12)	0.0215(13)	0.0273(12)	0.0006(10)	0.0051(10)	0.0045(10)
C13	0.0202(11)	0.0219(13)	0.0224(12)	–0.0018(10)	0.0061(9)	0.0023(9)
C14	0.0190(11)	0.0221(12)	0.0229(12)	0.000(1)	0.0047(9)	0.0015(9)
C15	0.0199(11)	0.0244(13)	0.0256(13)	–0.0012(10)	0.0042(9)	0.0014(10)
C16	0.0169(10)	0.0220(12)	0.0278(12)	–0.0014(10)	0.0067(9)	0.0005(9)
C17	0.0173(10)	0.0238(13)	0.0300(13)	0.0006(10)	0.0080(9)	–0.0001(9)
C18	0.0241(12)	0.0248(14)	0.0342(14)	0.0026(11)	0.0046(10)	–0.0057(10)
C19	0.0282(12)	0.0258(14)	0.0246(12)	–0.0001(11)	0.0087(10)	–0.0027(11)
C20	0.0217(11)	0.0217(13)	0.0230(12)	0.0002(10)	0.0044(9)	0.0012(10)

Table SI17. Bond Distances in Compound 18, Å

O1-C1	1.349(3)	O1-C17	1.467(3)	O2-C1	1.206(3)
O3-C14	1.429(3)	C1-C2	1.523(3)	C2-C3	1.553(3)
C2-C14	1.581(3)	C2-C20	1.549(3)	C3-C4	1.522(4)
C4-C5	1.532(3)	C5-C6	1.534(3)	C5-C7	1.548(3)
C5-C13	1.545(3)	C7-C8	1.539(4)	C7-C11	1.565(4)
C8-C9	1.534(4)	C8-C10	1.528(4)	C11-C12	1.544(4)
C12-C13	1.531(4)	C13-C14	1.535(4)	C14-C15	1.540(3)
C15-C16	1.525(4)	C16-C17	1.533(4)	C17-C18	1.515(4)
C17-C19	1.527(4)	C19-C20	1.559(3)		

Table SI18. Bond Angles in Compound 18, °

C1-O1-C17	119.6(2)	O1-C1-C2	117.49(19)	O2-C1-O1	117.0(2)
O2-C1-C2	125.4(2)	C1-C2-C3	105.56(19)	C1-C2-C14	110.71(19)
C1-C2-C20	108.2(2)	C3-C2-C14	110.2(2)	C20-C2-C3	110.7(2)
C20-C2-C14	111.28(19)	C4-C3-C2	115.9(2)	C3-C4-C5	110.5(2)
C4-C5-C6	111.6(2)	C4-C5-C7	115.8(2)	C4-C5-C13	105.8(2)
C6-C5-C7	109.8(2)	C6-C5-C13	113.1(2)	C13-C5-C7	100.15(19)
C5-C7-C11	103.9(2)	C8-C7-C5	118.3(2)	C8-C7-C11	111.2(2)

C9-C8-C7	110.6(2)	C10-C8-C7	114.2(2)	C10-C8-C9	109.0(2)
C12-C11-C7	106.8(2)	C13-C12-C11	103.59(19)	C12-C13-C5	104.3(2)
C12-C13-C14	118.4(2)	C14-C13-C5	118.2(2)	O3-C14-C2	104.20(19)
O3-C14-C13	114.6(2)	O3-C14-C15	109.21(19)	C13-C14-C2	109.30(19)
C13-C14-C15	107.5(2)	C15-C14-C2	112.1(2)	C16-C15-C14	115.9(2)
C15-C16-C17	114.42(19)	O1-C17-C16	108.88(19)	O1-C17-C18	103.2(2)
O1-C17-C19	108.00(19)	C18-C17-C16	110.3(2)	C18-C17-C19	111.9(2)
C19-C17-C16	114.0(2)	C17-C19-C20	113.0(2)	C2-C20-C19	113.9(2)

This report has been created with Olex2[12], compiled on 2022.04.07 svn.rca3783a0 for OlexSys.

X-ray Structure Determination of Compound 20

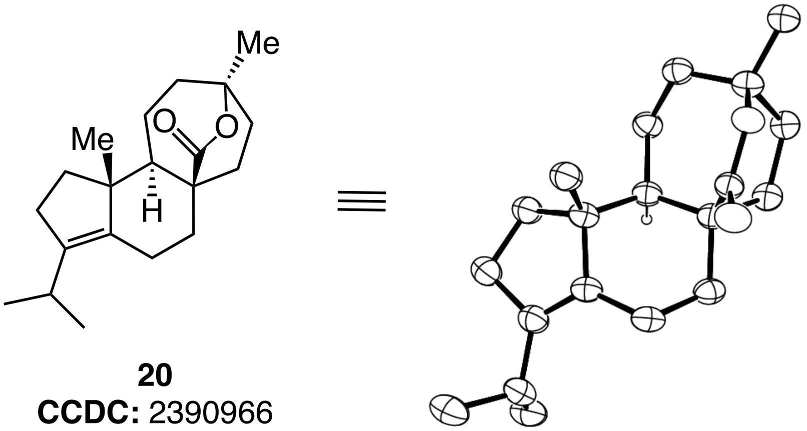

20
CCDC: 2390966

Compound **20**, $C_{20}H_{30}O_2$, crystallizes in the monoclinic space group $P2_1$ (systematic absences 0k0: k=odd) with a=8.6973(3) Å, b=10.4935(3) Å, c=9.4391(3) Å, α=90°, β=100.033(3)°, γ=90°, V=848.29(5) Å^3, Z=2, and d_{calc}=1.184 g/cm^3. X-ray intensity data were collected on a Rigaku XtaLAB Synergy-S diffractometer[7] equipped with an HPC area detector (HyPix-6000HE) and employing confocal multilayer optic-monochromated Cu-Kα radiation (λ=1.54184 Å) at a temperature of 100 K. Preliminary indexing was performed from a series ofsixty 0.5° rotation frames with exposures of 2.5 seconds for θ = ±47.554° and 10 seconds for θ = 113.25°. A total of 4762 frames (41 runs) were collected employing ω scans with a crystal to detector distance of 34.0 mm, rotation widths of 0.5° and exposures of 15 seconds.

Rotation frames were integrated using CrysAlisPro 8, producing a listing of unaveraged F^2 and $\sigma(F^2)$ values. A total of 13761 reflections were measured over the ranges $9.516 \leq 2\theta \leq 148.84°$, $-10 \leq h \leq 10$, $-13 \leq k \leq 12$, $-11 \leq l \leq 7$ yielding 3344 unique reflections (R_{int} = 0.0380). The intensity data were corrected for Lorentz and polarization effects and for absorption using SCALE3 ABSPACK[9] (minimum and maximum transmission 0.75966, 1.00000). The structure was solved by dual space methods – SHELXT.[10] Refinement was by full-matrix least squares based on F^2 using SHELXL.[11] All reflections were used during refinement. The weighting scheme used

was w=1/[$\sigma^2(F_o^2)$+ $(0.0840P)^2$ + 0.2212P] where P = $(F_o^2 + 2F_c^2)/3$. Non-hydrogen atoms were refined anisotropically and hydrogen atoms were refined using a riding model. Refinement converged to R1=0.0506 and wR2=0.1375 for 3028 observed reflections for which F > 4σ(F) and R1=0.0555 and wR2=0.1418 and GOF =1.091 for all 3344 unique, non-zero reflections and 203 variables. The maximum Δ/σ in the final cycle of least squares was 0.000 and the two most prominent peaks in the final difference Fourier were +0.38 and –0.25 e/Å^3.

Table SI19. lists cell information, data collection parameters, and refinement data. Final positional and equivalent isotropic thermal parameters are given in **Tables SI20**. and **SI21**. Anisotropic thermal parameters are in **Table SI22**. **Tables SI23**. and **SI24**. list bond distances and bond angles. **Figure 6**. is an ORTEP representation of **20** with 50% probability thermal ellipsoids displayed.

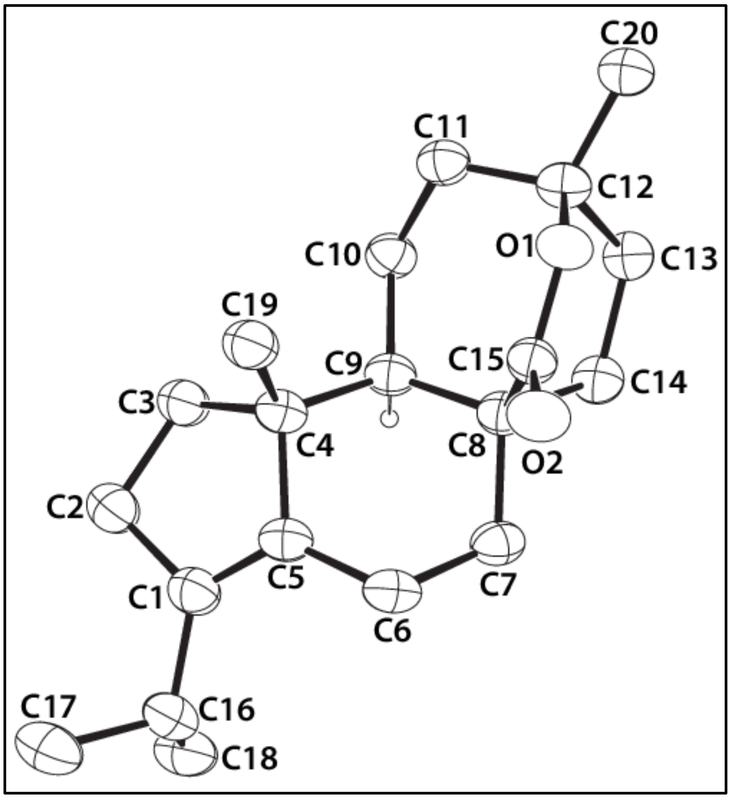

Figure 6. ORTEP drawing of **20** with 50% thermal ellipsoids.

Table SI19. Summary of Structure Determination of Compound 20

Empirical formula	$C_{20}H_{30}O_2$
Formula weight	302.44
Diffractometer	Rigaku XtaLAB Synergy-S (HyPix-6000HE)
Temperature/K	100
Crystal system	monoclinic
Space group	$P2_1$
a	8.6973(3) Å
b	10.4935(3) Å
c	9.4391(3) Å
α	90°
β	100.033(3)°
γ	90°
Volume	848.29(5) $Å^3$
Z	2
d_{calc}	1.184 g/cm^3
μ	0.572 mm^{-1}
F(000)	332.0
Crystal size, mm	0.156 × 0.047 × 0.036
2θ range for data collection	9.516 - 148.84°
Index ranges	$-10 \leq h \leq 10$, $-13 \leq k \leq 12$, $-11 \leq l \leq 7$
Reflections collected	13761
Independent reflections	3344[R(int) = 0.0380]
Data/restraints/parameters	3344/1/203
Goodness-of-fit on F^2	1.091
Final R indexes [I>=2σ (I)]	$R_1 = 0.0506$, $wR_2 = 0.1375$
Final R indexes [all data]	$R_1 = 0.0555$, $wR_2 = 0.1418$
Largest diff. peak/hole	0.38/–0.25 $eÅ^{-3}$
Flack parameter	0.08(10)

Table SI20. Refined Positional Parameters for Compound 20

Atom	*x*	*y*	*z*	U(eq)
O1	0.0861(2)	0.4402(2)	1.0425(2)	0.0322(5)
O2	0.2957(3)	0.3329(2)	1.0213(3)	0.0374(6)
C1	0.4293(4)	0.4752(3)	0.5346(4)	0.0385(8)
C2	0.2924(4)	0.5080(5)	0.4197(4)	0.0470(9)
C3	0.1690(4)	0.5545(4)	0.5069(4)	0.0420(8)
C4	0.2130(4)	0.4922(3)	0.6574(4)	0.0348(7)
C5	0.3864(4)	0.4642(3)	0.6631(4)	0.0340(7)
C6	0.4836(4)	0.4346(4)	0.8052(4)	0.0369(7)
C7	0.4560(4)	0.5299(3)	0.9207(4)	0.0354(7)
C8	0.2833(4)	0.5497(3)	0.9308(3)	0.0313(7)
C9	0.1952(4)	0.5881(3)	0.7776(3)	0.0319(7)
C10	0.0292(4)	0.6413(3)	0.7732(4)	0.0356(7)
C11	–0.0752(4)	0.5751(3)	0.8656(4)	0.0355(7)
C12	–0.0033(4)	0.5601(3)	1.0231(4)	0.0325(7)
C13	0.1063(4)	0.6683(3)	1.0831(4)	0.0344(7)
C14	0.2673(4)	0.6596(3)	1.0364(4)	0.0349(7)
C15	0.2236(4)	0.4319(3)	0.9968(3)	0.0308(7)
C16	0.5912(4)	0.4603(4)	0.4989(4)	0.0409(8)
C17	0.5910(5)	0.3730(4)	0.3689(5)	0.0521(10)
C18	0.6598(5)	0.5898(4)	0.4705(5)	0.0512(10)
C19	0.1197(4)	0.3680(4)	0.6614(4)	0.0397(8)
C20	–0.1308(4)	0.5387(4)	1.1130(4)	0.0377(7)

Table SI21. Positional Parameters for Hydrogens in Compound 20.

Atom	*x*	*y*	*z*	U(eq)
H2A	0.254971	0.432336	0.361066	0.056
H2B	0.319663	0.576003	0.355678	0.056
H3A	0.063115	0.527794	0.46012	0.05
H3B	0.171145	0.648618	0.51507	0.05
H6A	0.595243	0.43615	0.796004	0.044
H6B	0.458501	0.347693	0.834981	0.044
H7A	0.501437	0.613033	0.900452	0.043
H7B	0.512055	0.499965	1.015161	0.043

H9	0.255014	0.663989	0.753184	0.038
H10A	0.038915	0.731913	0.802395	0.043
H10B	–0.025341	0.63898	0.67198	0.043
H11A	–0.173199	0.624455	0.859209	0.043
H11B	–0.102966	0.489575	0.824841	0.043
H13A	0.120979	0.666655	1.189479	0.041
H13B	0.057156	0.750686	1.05023	0.041
H14A	0.348206	0.648926	1.123332	0.042
H14B	0.288245	0.741165	0.990547	0.042
H16	0.659657	0.420909	0.583731	0.049
H17A	0.553479	0.288255	0.390049	0.078
H17B	0.697244	0.366247	0.348224	0.078
H17C	0.521986	0.408765	0.285094	0.078
H18A	0.594708	0.629762	0.387123	0.077
H18B	0.765975	0.578308	0.451019	0.077
H18C	0.662724	0.644467	0.555105	0.077
H19A	0.159558	0.321534	0.750275	0.06
H19B	0.130945	0.315128	0.57829	0.06
H19C	0.009188	0.388396	0.658274	0.06
H20A	–0.196395	0.466755	1.073493	0.057
H20B	–0.195141	0.615611	1.11055	0.057
H20C	–0.082733	0.520033	1.212574	0.057

Table SI22. Refined Thermal Parameters (U's) for Compound 20

Atom	U_{11}	U_{22}	U_{33}	U_{23}	U_{13}	U_{12}
O1	0.0305(11)	0.0244(11)	0.0437(12)	0.0003(9)	0.0122(9)	0.0015(9)
O2	0.0361(12)	0.0283(12)	0.0496(14)	0.0078(10)	0.0125(10)	0.0050(9)
C1	0.0360(17)	0.038(2)	0.0427(18)	–0.0025(14)	0.0106(13)	0.0034(14)
C2	0.0406(19)	0.064(3)	0.0384(19)	0.0011(17)	0.0114(16)	0.0070(17)
C3	0.0349(17)	0.053(2)	0.0387(18)	0.0029(17)	0.0095(14)	0.0059(16)
C4	0.0301(16)	0.0359(17)	0.0396(17)	0.0016(14)	0.0095(13)	0.0035(14)
C5	0.0285(15)	0.0312(17)	0.0431(17)	0.0010(13)	0.0088(12)	0.0028(12)
C6	0.0283(15)	0.0374(18)	0.0466(18)	0.0057(15)	0.0112(13)	0.0039(14)
C7	0.0284(15)	0.0369(19)	0.0414(18)	0.0076(13)	0.0072(13)	–0.0017(13)
C8	0.0292(15)	0.0279(15)	0.0377(17)	0.0003(12)	0.0082(13)	–0.0030(12)
C9	0.0288(16)	0.0275(15)	0.0406(18)	0.0017(13)	0.0091(13)	0.0000(12)
C10	0.0325(16)	0.0349(18)	0.0396(17)	0.0035(13)	0.0072(13)	0.0073(13)

C11	0.0304(16)	0.0328(17)	0.0436(18)	–0.0022(14)	0.0074(13)	0.0017(13)
C12	0.0312(16)	0.0229(15)	0.0444(18)	0.0009(13)	0.0094(13)	0.0038(12)
C13	0.0360(17)	0.0271(16)	0.0403(17)	–0.0021(13)	0.0068(13)	–0.0007(13)
C14	0.0359(17)	0.0270(17)	0.0420(17)	0.0001(13)	0.0079(14)	–0.0064(13)
C15	0.0301(15)	0.0291(17)	0.0337(16)	0.0003(12)	0.0072(12)	0.0003(13)
C16	0.0383(18)	0.044(2)	0.0436(18)	0.0020(16)	0.0162(14)	0.0053(15)
C17	0.046(2)	0.051(2)	0.066(3)	–0.0023(19)	0.027(2)	–0.0008(18)
C18	0.039(2)	0.048(2)	0.069(3)	–0.006(2)	0.0178(18)	0.0022(17)
C19	0.0352(18)	0.040(2)	0.046(2)	–0.0069(15)	0.0118(15)	–0.0018(14)
C20	0.0327(16)	0.0374(18)	0.0442(19)	–0.0008(14)	0.0103(14)	0.0012(14)

Table SI23. Bond Distances in Compound 20, Å

O1-C12	1.474(4)	O1-C15	1.343(4)	O2-C15	1.215(4)
C1-C2	1.505(5)	C1-C5	1.334(5)	C1-C16	1.512(4)
C2-C3	1.542(5)	C3-C4	1.550(5)	C4-C5	1.528(4)
C4-C9	1.544(5)	C4-C19	1.539(5)	C5-C6	1.488(5)
C6-C7	1.529(5)	C7-C8	1.536(4)	C8-C9	1.567(5)
C8-C14	1.546(5)	C8-C15	1.516(4)	C9-C10	1.542(4)
C10-C11	1.531(5)	C11-C12	1.516(5)	C12-C13	1.527(5)
C12-C20	1.525(5)	C13-C14	1.543(5)	C16-C17	1.531(6)
C16-C18	1.527(6)				

Table SI24. Bond Angles in Compound 20, °

C15-O1-C12	119.7(2)	C2-C1-C16	120.9(3)	C5-C1-C2	111.3(3)
C5-C1-C16	127.8(3)	C1-C2-C3	103.0(3)	C2-C3-C4	105.8(3)
C5-C4-C3	101.4(3)	C5-C4-C9	108.9(3)	C5-C4-C19	111.0(3)
C9-C4-C3	111.0(3)	C19-C4-C3	109.4(3)	C19-C4-C9	114.4(3)
C1-C5-C4	112.5(3)	C1-C5-C6	129.2(3)	C6-C5-C4	118.3(3)
C5-C6-C7	111.8(3)	C6-C7-C8	114.2(3)	C7-C8-C9	107.7(3)
C7-C8-C14	109.8(3)	C14-C8-C9	108.6(3)	C15-C8-C7	108.9(3)
C15-C8-C9	116.2(3)	C15-C8-C14	105.5(2)	C4-C9-C8	114.6(3)
C10-C9-C4	115.4(3)	C10-C9-C8	115.0(3)	C11-C10-C9	117.8(3)

C12-C11-C10	114.9(3)	O1-C12-C11	109.2(3)	O1-C12-C13	107.9(2)
O1-C12-C20	102.9(3)	C11-C12-C13	114.2(3)	C11-C12-C20	110.2(3)
C20-C12-C13	111.7(3)	C12-C13-C14	112.9(3)	C13-C14-C8	114.6(3)
O1-C15-C8	117.8(3)	O2-C15-O1	117.0(3)	O2-C15-C8	125.0(3)
C1-C16-C17	111.7(3)	C1-C16-C18	110.8(3)	C18-C16-C17	109.7(3)

This report has been created with Olex2[12], compiled on 2022.04.07 svn.rca3783a0 for OlexSys.

X-ray Structure Determination of Compound 8

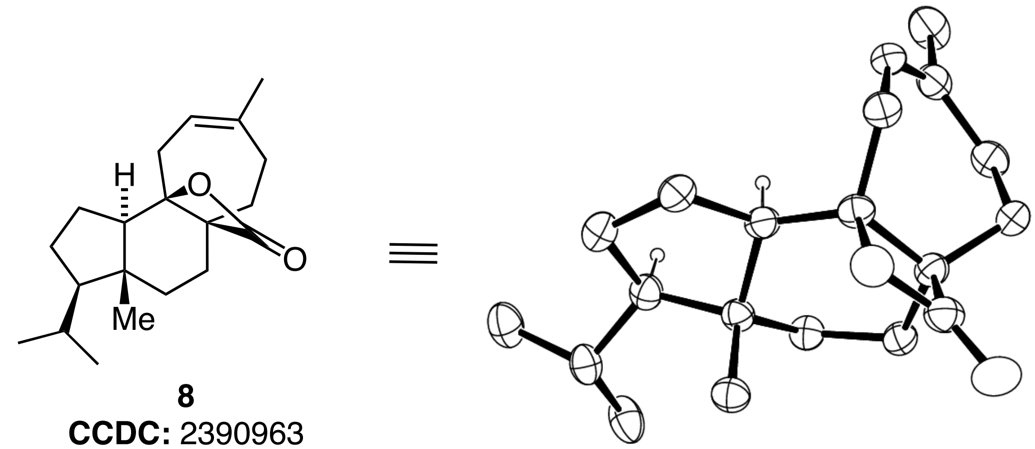

8
CCDC: 2390963

Compound **8**, $C_{20}H_{30}O_2$, crystallizes in the trigonal space group $P3_1$ (systematic absences 00l: l≠3n) with a=7.09270(10) Å, b=7.09270(10) Å, c=29.8613(6) Å, α=90°, β=90°, γ=120°, V=1300.95(5) Å^3, Z=3, and d_{calc}=1.158 g/cm^3. X-ray intensity data were collected on a Rigaku XtaLAB Synergy-S diffractometer[7] equipped with an HPC area detector (HyPix-6000HE) and employing confocal multilayer optic-monochromated Cu-Kα radiation (λ=1.54184 Å) at a temperature of 100 K. Preliminary indexing was performed from a series of sixty 0.5° rotation frames with exposures of 1.25 seconds for θ = ±47.554° and 5 seconds for θ = 113.25°. A total of 4211 frames (43 runs) were collected employing ω scans with a crystal to detector distance of 34.0 mm, rotation widths of 0.5° and exposures of 2 seconds.

Rotation frames were integrated using CrysAlisPro[8] producing a listing of unaveraged F^2 and $\sigma(F^2)$ values. A total of 18449 reflections were measured over the ranges 8.884 ≤ 2θ ≤ 148.914°, –8 ≤ h ≤ 8, –8 ≤ k ≤ 8, –36 ≤ l ≤ 37 yielding 3459 unique reflections (R_{int} = 0.0355). The intensity data were corrected for Lorentz and polarization effects and for absorption using SCALE3 ABSPACK[9] (minimum and maximum transmission 0.72432, 1.00000). The structure was

solved by dual space methods – SHELXT[10]. Refinement was by full-matrix least squares based on F^2 using SHELXL[11]All reflections were used during refinement. The weighting scheme used was $w=1/[\sigma^2(F_o^2) + (0.0559P)^2 + 0.1169P]$ where $P = (F_o^2 + 2F_c^2)/3$. Non-hydrogen atoms were refined anisotropically and hydrogen atoms were refined using a riding model. Refinement converged to R1=0.0363 and wR2=0.0905 for 3279 observed reflections for which $F > 4\sigma(F)$ and R1=0.0389 and wR2=0.0946 and GOF =1.111 for all 3459 unique, non-zero reflections and 203 variables. The maximum Δ/σ in the final cycle of least squares was 0.000 and the two most prominent peaks in the final difference Fourier were +0.16 and –0.17 e/Å^3.

Table SI25. lists cell information, data collection parameters, and refinement data. Final positional and equivalent isotropic thermal parameters are given in **Tables SI26**. and **SI27**. Anisotropic thermal parameters are in **Table SI28**. **Tables SI29** and **SI30**. list bond distances and bond angles. **Figure 7**. is an ORTEP representation of **8** with 50% probability thermal ellipsoids displayed.

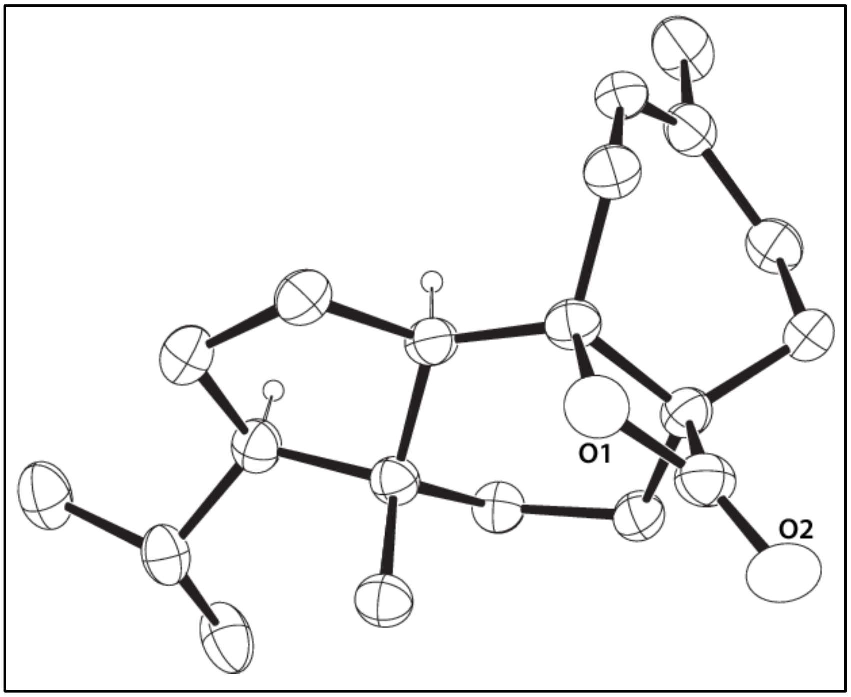

Figure 7. ORTEP drawing of **8** with 50% thermal ellipsoids.

Table SI25. Summary of Structure Determination of Compound 8

Empirical formula	$C_{20}H_{30}O_2$
Formula weight	302.44
Diffractometer	Rigaku XtaLAB Synergy-S (HyPix-6000HE)
Temperature/K	100
Crystal system	trigonal
Space group	$P3_1$
a	7.09270(10) Å
b	7.09270(10) Å
c	29.8613(6) Å
α	90°
β	90°
γ	120°
Volume	1300.95(5) Å^3
Z	3
d_{calc}	1.158 g/cm^3
μ	0.559 mm^{-1}
F(000)	498.0
Crystal size, mm	0.221 × 0.104 × 0.029
2θ range for data collection	8.884 - 148.914°
Index ranges	–8 ≤ h ≤ 8, –8 ≤ k ≤ 8, –36 ≤ l ≤ 37
Reflections collected	18449
Independent reflections	3459[R(int) = 0.0355]
Data/restraints/parameters	3459/1/203
Goodness-of-fit on F^2	1.111
Final R indexes [I>=2σ (I)]	R_1 = 0.0363, wR_2 = 0.0905
Final R indexes [all data]	R_1 = 0.0389, wR_2 = 0.0946
Largest diff. peak/hole	0.16/-0.17 eÅ^{-3}
Flack parameter	–0.05(11)

Table SI26. Refined Positional Parameters for Compound 8

Atom	*x*	*y*	*z*	U(eq)
O1	0.2806(3)	0.3364(2)	0.52551(6)	0.0321(4)
O2	0.5753(3)	0.3110(3)	0.54745(7)	0.0411(4)
C1	0.3693(3)	0.8767(3)	0.43293(8)	0.0293(5)
C2	0.1230(4)	0.7013(4)	0.42841(9)	0.0362(5)
C3	0.0650(4)	0.5474(4)	0.46900(9)	0.0336(5)
C4	0.2653(3)	0.6652(3)	0.49886(8)	0.0270(4)
C5	0.4592(3)	0.7698(3)	0.46588(7)	0.0256(4)
C6	0.4754(4)	0.9551(4)	0.38681(8)	0.0323(5)
C7	0.7173(4)	1.1220(4)	0.38878(9)	0.0447(6)
C8	0.3592(5)	1.0551(5)	0.36128(10)	0.0431(6)
C9	0.5011(4)	0.6051(4)	0.44086(8)	0.0298(5)
C10	0.6577(4)	0.9285(3)	0.49359(8)	0.0292(5)
C11	0.7111(3)	0.8151(4)	0.53134(8)	0.0292(5)
C12	0.5170(3)	0.6325(3)	0.55797(8)	0.0273(5)
C13	0.2835(3)	0.5443(3)	0.53845(8)	0.0270(4)
C14	0.1046(4)	0.4824(4)	0.57289(8)	0.0323(5)
C15	0.1158(4)	0.6765(4)	0.59616(8)	0.0337(5)
C16	0.2862(4)	0.8387(4)	0.61727(8)	0.0332(5)
C17	0.5087(4)	0.8636(4)	0.62254(9)	0.0333(5)
C18	0.5382(3)	0.6730(4)	0.60826(8)	0.0294(5)
C19	0.2659(5)	1.0206(5)	0.63839(10)	0.0472(7)
C20	0.4779(4)	0.4089(4)	0.54464(8)	0.0311(5)

Table SI27. Positional Parameters for Hydrogens in Compound 8.

Atom	*x*	*y*	*z*	U(eq)
H1	0.377227	1.005032	0.448611	0.035
H2A	0.0325	0.771606	0.42835	0.043
H2B	0.096933	0.619036	0.400131	0.043
H3A	–0.06693	0.528321	0.484418	0.04
H3B	0.041109	0.402933	0.459768	0.04
H4	0.256225	0.790163	0.511963	0.032
H6	0.456802	0.825948	0.369593	0.039
H7A	0.739831	1.245493	0.407339	0.067

H7B	0.77159	1.173096	0.358454	0.067
H7C	0.796294	1.05468	0.401831	0.067
H8A	0.205809	0.944852	0.357102	0.065
H8B	0.428379	1.106939	0.331992	0.065
H8C	0.369198	1.177616	0.378433	0.065
H9A	0.541388	0.526981	0.46237	0.045
H9B	0.61977	0.682288	0.419341	0.045
H9C	0.36875	0.501038	0.424882	0.045
H10A	0.62997	1.040326	0.506829	0.035
H10B	0.785436	1.003569	0.473539	0.035
H11A	0.808073	0.927982	0.552956	0.035
H11B	0.794979	0.751502	0.518143	0.035
H14A	–0.038418	0.398934	0.557875	0.039
H14B	0.11491	0.386231	0.595604	0.039
H15	–0.013855	0.68465	0.59578	0.04
H17A	0.550443	0.894628	0.654473	0.04
H17B	0.612936	0.993304	0.605222	0.04
H18A	0.683743	0.702061	0.618007	0.035
H18B	0.428024	0.539568	0.623732	0.035
H19A	0.12395	1.004057	0.630813	0.071
H19B	0.381892	1.160708	0.627114	0.071
H19C	0.278596	1.01531	0.670987	0.071

Table SI28. Refined Thermal Parameters (U's) for Compound 8

Atom	U_{11}	U_{22}	U_{33}	U_{23}	U_{13}	U_{12}
O1	0.0359(8)	0.0238(7)	0.0354(9)	–0.0019(6)	0.0008(7)	0.0141(6)
O2	0.0459(10)	0.0385(9)	0.0498(12)	0.0047(8)	0.0103(8)	0.0292(8)
C1	0.0321(11)	0.0283(10)	0.0288(12)	–0.0036(9)	–0.0038(9)	0.0162(9)
C2	0.0303(11)	0.0437(13)	0.0355(14)	–0.0001(10)	–0.005(1)	0.0191(10)
C3	0.0243(10)	0.0397(12)	0.0338(13)	–0.0017(10)	–0.0020(9)	0.0139(9)
C4	0.0245(9)	0.0295(10)	0.0283(12)	–0.0032(9)	–0.0001(9)	0.0144(8)
C5	0.0266(10)	0.0255(10)	0.0248(11)	–0.0027(8)	–0.0004(8)	0.0130(8)
C6	0.0386(12)	0.0287(10)	0.0268(12)	–0.0035(9)	–0.006(1)	0.0147(9)
C7	0.0438(14)	0.0412(14)	0.0305(14)	0.0053(11)	–0.0024(11)	0.0073(11)
C8	0.0603(16)	0.0421(13)	0.0327(13)	0.0002(11)	–0.0055(12)	0.0299(13)
C9	0.0314(11)	0.0288(10)	0.0301(13)	–0.0029(9)	0.0010(9)	0.0157(9)

C10	0.0268(10)	0.0259(10)	0.0316(13)	–0.0008(9)	–0.0001(9)	0.0107(8)
C11	0.0241(9)	0.0309(11)	0.0308(12)	0.0005(9)	–0.0005(9)	0.0125(8)
C12	0.0273(10)	0.0283(10)	0.0291(12)	0.0008(9)	0.0004(9)	0.0160(8)
C13	0.026(1)	0.0252(10)	0.0299(12)	–0.0023(8)	0.0007(9)	0.0128(8)
C14	0.0266(10)	0.0363(11)	0.0316(13)	0.0019(9)	0.0010(9)	0.0140(9)
C15	0.0343(11)	0.0468(13)	0.0302(13)	0.0049(10)	0.0052(10)	0.0280(11)
C16	0.0457(13)	0.0343(11)	0.0293(12)	0.0052(9)	0.0064(11)	0.0273(10)
C17	0.0365(12)	0.0306(10)	0.0304(12)	–0.0051(9)	–0.0002(10)	0.0151(9)
C18	0.0279(10)	0.0325(10)	0.0307(13)	0.0009(9)	–0.0014(9)	0.0174(9)
C19	0.0737(19)	0.0436(14)	0.0411(16)	0.0029(12)	0.0081(14)	0.0420(14)
C20	0.0339(11)	0.0309(11)	0.0320(13)	0.0030(9)	0.0059(9)	0.0189(9)

Table SI29. Bond Distances in Compound 8, Å

O1-C13	1.515(2)	O1-C20	1.352(3)	O2-C20	1.203(3)
C1-C2	1.564(3)	C1-C5	1.560(3)	C1-C6	1.534(3)
C2-C3	1.543(4)	C3-C4	1.524(3)	C4-C5	1.546(3)
C4-C13	1.503(3)	C5-C9	1.536(3)	C5-C10	1.533(3)
C6-C7	1.522(3)	C6-C8	1.532(3)	C10-C11	1.538(3)
C11-C12	1.556(3)	C12-C13	1.561(3)	C12-C18	1.522(3)
C12-C20	1.520(3)	C13-C14	1.518(3)	C14-C15	1.509(3)
C15-C16	1.338(4)	C16-C17	1.506(3)	C16-C19	1.506(3)
C17-C18	1.529(3)				

Table SI30. Bond Angles in Compound 8, °

C20-O1-C13	90.81(15)	C5-C1-C2	103.50(18)	C6-C1-C2	111.2(2)
C6-C1-C5	119.80(18)	C3-C2-C1	106.94(19)	C4-C3-C2	103.06(19)
C3-C4-C5	104.59(18)	C13-C4-C3	118.01(19)	C13-C4-C5	117.70(17)
C4-C5-C1	98.83(16)	C9-C5-C1	110.13(18)	C9-C5-C4	114.13(18)
C10-C5-C1	115.61(17)	C10-C5-C4	106.19(18)	C10-C5-C9	111.41(17)
C7-C6-C1	113.89(19)	C7-C6-C8	108.5(2)	C8-C6-C1	109.5(2)
C5-C10-C11	112.80(18)	C10-C11-C12	117.57(18)	C11-C12-C13	117.54(19)
C18-C12-C11	112.60(18)	C18-C12-C13	115.41(18)	C20-C12-C11	110.99(18)

C20-C12-C13	83.14(16)	C20-C12-C18	113.87(18)	O1-C13-C12	88.90(14)
O1-C13-C14	106.76(17)	C4-C13-O1	113.05(18)	C4-C13-C12	116.22(18)
C4-C13-C14	113.58(18)	C14-C13-C12	115.4(2)	C15-C14-C13	113.14(19)
C16-C15-C14	127.9(2)	C15-C16-C17	126.4(2)	C15-C16-C19	120.1(2)
C17-C16-C19	113.5(2)	C16-C17-C18	117.77(19)	C12-C18-C17	113.44(19)
O1-C20-C12	96.99(16)	O2-C20-O1	126.4(2)	O2-C20-C12	136.6(2)

This report has been created with Olex2[12] compiled on 2022.04.07 svn.rca3783a0 for OlexSys.

X-ray Structure Determination of Compound 21

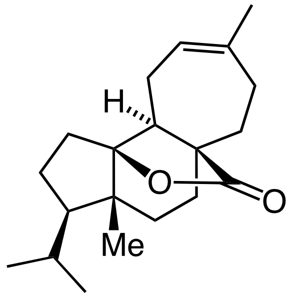

21
CCDC: 2390967

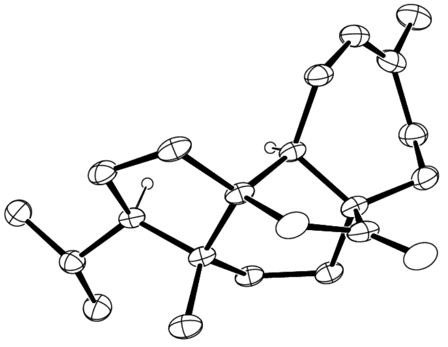

Compound **21**, $C_{20}H_{30}O_2$, crystallizes in the monoclinic space group $P2_1$ (systematic absences 0k0: k=odd) with a=6.4078(4) Å, b=21.6854(13) Å, c=12.5982(7) Å, α=90°, β=100.189(6)°, γ=90°, V=1722.98(18) $Å^3$, Z=4, and d_{calc}=1.166 g/cm^3. X-ray intensity data were collected on a Rigaku XtaLAB Synergy-S diffractometer[7] equipped with an HPC area detector (Dectris Pilatus3 R 200 K) and employing confocal multilayer optic-monochromated Mo-Kα radiation (λ=0.71073 Å) at a temperature of 100 K. Preliminary indexing was performed from a series of thirty 0.5° rotation frames with exposures of 1.25 seconds. A total of 1202 frames (10 runs) were collected employing ω scans with a crystal to detector distance of 34.0 mm, rotation widths of 0.5° and exposures of 10.45 seconds.

Rotation frames were integrated using CrysAlisPro[8], producing a listing of unaveraged F^2 and $\sigma(F^2)$ values. A total of 34543 reflections were measured over the ranges $3.784 \leq 2\theta \leq 56.554°$, $-8 \leq h \leq 8$, $-28 \leq k \leq 28$, $-16 \leq l \leq 16$ yielding 8366 unique reflections (R_{int} = 0.0634). The intensity data were corrected for Lorentz and polarization effects and for absorption using SCALE3 ABSPACK 9 (minimum and maximum transmission 0.60356, 1.00000). The structure was solved by dual space methods – SHELXT[10]. Refinement was by full-matrix least squares based on F^2 using SHELXL[11] All reflections were used during refinement. The weighting scheme used was $w=1/[\sigma^2(F_o^2)+(0.0951P)^2+0.3890P]$ where $P=(F_o^2+2F_c^2)/3$. Non-hydrogen atoms

were refined anisotropically and hydrogen atoms were refined using a riding model. Refinement converged to R1=0.0570 and wR2=0.1465 for 7324 observed reflections for which $F > 4\sigma(F)$ and R1=0.0657 and wR2=0.1508 and GOF =1.020 for all 8366 unique, non-zero reflections and 405 variables. The maximum Δ/σ in the final cycle of least squares was 0.000 and the two most prominent peaks in the final difference Fourier were +0.41 and –0.24 e/Å^3.

Table SI31. lists cell information, data collection parameters, and refinement data. Final positional and equivalent isotropic thermal parameters are given in **Tables SI32**. and **SI33**. Anisotropic thermal parameters are in **Table SI34**. **Tables SI35**. and **SI36**. list bond distances and bond angles. **Figure 8**. is an ORTEP representation of **21** with 50% probability thermal ellipsoids displayed.

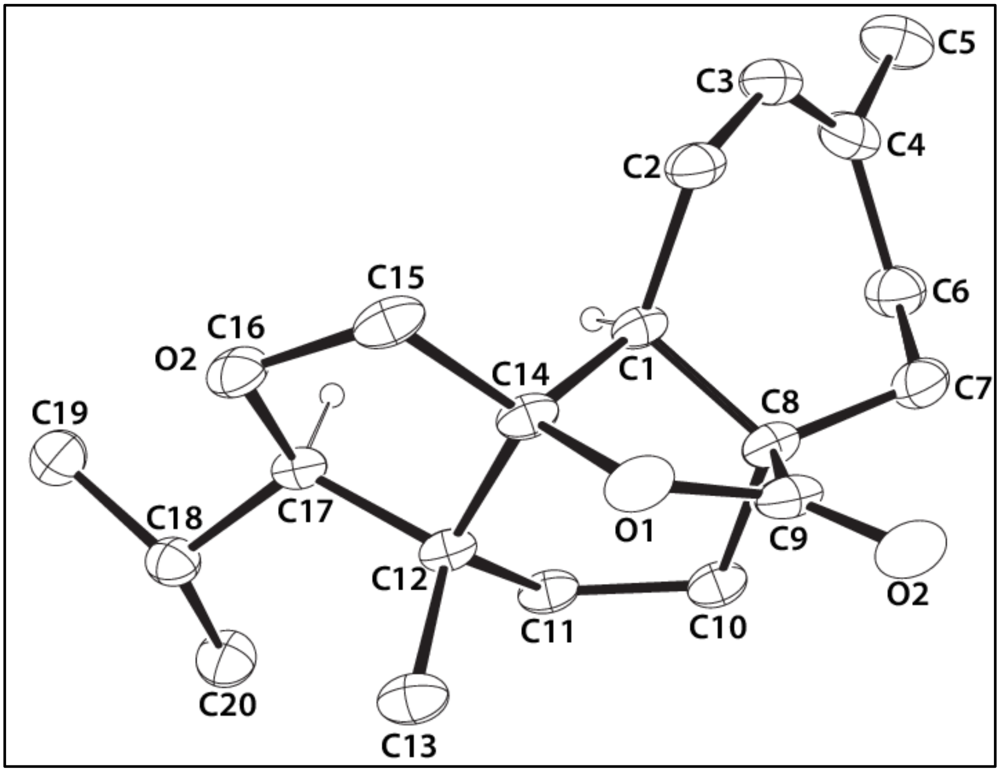

Figure 8. ORTEP drawing of **21** with 50% thermal ellipsoids.

Table SI31. Summary of Structure Determination of Compound 21

Empirical formula	$C_{20}H_{30}O_2$
Formula weight	302.44
Diffractometer	Rigaku XtaLAB Synergy-S (Dectris Pilatus3 R 200 K)
Temperature/K	100
Crystal system	monoclinic
Space group	$P2_1$
a	6.4078(4) Å
b	21.6854(13) Å
c	12.5982(7) Å
α	90°
β	100.189(6)°
γ	90°
Volume	1722.98(18) $Å^3$
Z	4
d_{calc}	1.166 g/cm^3
μ	0.073 mm^{-1}
F(000)	664.0
Crystal size, mm	0.35 × 0.246 × 0.08
2θ range for data collection	3.784 - 56.554°
Index ranges	$-8 \le h \le 8$, $-28 \le k \le 28$, $-16 \le l \le 16$
Reflections collected	34543
Independent reflections	8366[R(int) = 0.0634]
Data/restraints/parameters	8366/1/405
Goodness-of-fit on F^2	1.020
Final R indexes [I>=2σ (I)]	$R_1 = 0.0570$, $wR_2 = 0.1465$
Final R indexes [all data]	$R_1 = 0.0657$, $wR_2 = 0.1508$
Largest diff. peak/hole	0.41/–0.24 $eÅ^{-3}$
Flack parameter	0.5(6)

Table SI32. Refined Positional Parameters for Compound 21

Atom	*x*	*y*	*z*	U(eq)
O1*	–0.3838(3)	–0.45654(10)	–0.19226(17)	0.0215(5)
O2*	–0.4343(4)	–0.51322(12)	–0.34282(18)	0.0278(5)
C1*	–0.0576(5)	–0.49699(14)	–0.1070(2)	0.0168(6)
C2*	–0.1263(5)	–0.55424(15)	–0.0504(2)	0.0210(6)
C3*	0.0391(5)	–0.60460(15)	–0.0326(3)	0.0229(6)
C4*	0.1778(5)	–0.62042(14)	–0.0948(3)	0.0244(7)
C5*	0.3427(6)	–0.66929(17)	–0.0579(3)	0.0332(8)
C6*	0.1882(5)	–0.59436(15)	–0.2055(3)	0.0257(7)
C7*	–0.0241(5)	–0.57089(14)	–0.2665(2)	0.0218(6)
C8*	–0.0811(5)	–0.50649(13)	–0.2306(2)	0.0167(6)
C9*	–0.3169(5)	–0.49460(14)	–0.2650(2)	0.0203(6)
C10*	0.0362(5)	–0.45437(14)	–0.2806(2)	0.0184(6)
C11*	0.0860(5)	–0.39792(14)	–0.2068(2)	0.0177(6)
C12*	–0.0883(4)	–0.38042(14)	–0.1417(2)	0.0161(6)
C13*	–0.2517(5)	–0.33705(15)	–0.2077(2)	0.0223(6)
C14*	–0.2012(5)	–0.43994(15)	–0.1086(2)	0.0191(6)
C15*	–0.2870(5)	–0.42345(16)	–0.0057(3)	0.0251(7)
C16*	–0.1712(5)	–0.36465(15)	0.0390(2)	0.0220(6)
C17*	0.0027(5)	–0.35280(14)	–0.0288(2)	0.0170(6)
C18*	0.0741(5)	–0.28478(14)	–0.0220(2)	0.0206(6)
C19*	0.1597(6)	–0.26804(17)	0.0962(3)	0.0290(7)
C20*	0.2402(5)	–0.26895(16)	–0.0904(3)	0.0251(7)
O1	–0.8835(3)	–0.58153(11)	–0.68918(17)	0.0235(5)
O2	–0.8932(4)	–0.66994(12)	–0.78051(19)	0.0296(5)
C1	–0.5517(5)	–0.59359(14)	–0.5766(2)	0.0187(6)
C2	–0.6057(5)	–0.63479(15)	–0.4859(2)	0.0242(7)
C3	–0.4211(6)	–0.67346(16)	–0.4316(3)	0.0260(7)
C4	–0.2663(6)	–0.69917(16)	–0.4731(3)	0.0284(7)
C5	–0.0888(6)	–0.7331(2)	–0.4029(3)	0.0368(8)
C6	–0.2495(6)	–0.69861(17)	–0.5923(3)	0.0297(7)
C7	–0.4617(6)	–0.69526(16)	–0.6690(3)	0.0270(7)
C8	–0.5544(5)	–0.62988(15)	–0.6821(2)	0.0215(6)
C9	–0.7909(5)	–0.63219(16)	–0.7240(2)	0.0228(6)
C10	–0.4567(5)	–0.59042(15)	–0.7644(2)	0.0223(6)
C11	–0.4370(5)	–0.52155(15)	–0.7335(2)	0.0214(6)
C12	–0.6254(5)	–0.49462(14)	–0.6876(2)	0.0188(6)

C13	–0.7976(5)	–0.47129(17)	–0.7796(2)	0.0257(7)
C14	–0.7212(5)	–0.54454(14)	–0.6198(2)	0.0192(6)
C15	–0.8286(5)	–0.50899(16)	–0.5394(2)	0.0231(6)
C16	–0.7401(5)	–0.44309(16)	–0.5347(3)	0.0244(7)
C17	–0.5580(5)	–0.44407(14)	–0.5998(2)	0.0186(6)
C18	–0.5052(5)	–0.37841(14)	–0.6343(3)	0.0228(6)
C19	–0.4224(7)	–0.33993(17)	–0.5337(3)	0.0322(8)
C20	–0.3437(6)	–0.37536(17)	–0.7097(3)	0.0297(7)

Table SI33. Positional Parameters for Hydrogens in Compound 21.

Atom	*x*	*y*	*z*	U(eq)
H1*	0.092274	–0.486001	–0.075734	0.02
H2*A	–0.159727	–0.541743	0.020334	0.025
H2*B	–0.257608	–0.570968	–0.094166	0.025
H3*	0.044864	–0.62807	0.031506	0.028
H5*A	0.484579	–0.651752	–0.054873	0.05
H5*B	0.323033	–0.703862	–0.108786	0.05
H5*C	0.327422	–0.683947	0.013906	0.05
H6*A	0.241482	–0.626793	–0.249162	0.031
H6*B	0.291516	–0.559989	–0.197277	0.031
H7*A	–0.137022	–0.60014	–0.255539	0.026
H7*B	–0.019128	–0.569887	–0.344541	0.026
H10A	0.170556	–0.470991	–0.297366	0.022
H10B	–0.052277	–0.441111	–0.349363	0.022
H11A	0.111371	–0.362124	–0.251563	0.021
H11B	0.219064	–0.405983	–0.155509	0.021
H13A	–0.18479	–0.297276	–0.217182	0.033
H13B	–0.370655	–0.33079	–0.169478	0.033
H13C	–0.303931	–0.355439	–0.278455	0.033
H15A	–0.441922	–0.416232	–0.022612	0.03
H15B	–0.258748	–0.457323	0.047619	0.03
H16A	–0.107364	–0.370171	0.11592	0.026
H16B	–0.271057	–0.32946	0.032875	0.026
H17*	0.128332	–0.378298	0.002876	0.02
H18*	–0.053563	–0.258639	–0.047349	0.025
H19A	0.282782	–0.29393	0.123657	0.044

H19B	0.04888	–0.274955	0.139471	0.044
H19C	0.201967	–0.224554	0.100932	0.044
H20A	0.181654	–0.27617	–0.1666	0.038
H20B	0.365511	–0.295002	–0.06873	0.038
H20C	0.280498	–0.225496	–0.079735	0.038
H1	–0.410455	–0.57353	–0.553398	0.022
H2A	–0.654565	–0.608398	–0.431089	0.029
H2B	–0.724112	–0.662475	–0.51629	0.029
H3	–0.415346	–0.680317	–0.356663	0.031
H5A	–0.108666	–0.731135	–0.327635	0.055
H5B	0.046912	–0.71409	–0.409605	0.055
H5C	–0.089145	–0.77634	–0.42581	0.055
H6A	–0.162279	–0.662799	–0.606103	0.036
H6B	–0.17443	–0.736382	–0.608646	0.036
H7A	–0.564116	–0.722667	–0.641897	0.032
H7B	–0.443071	–0.710805	–0.740651	0.032
H10C	–0.31438	–0.606757	–0.769105	0.027
H10D	–0.546532	–0.594469	–0.836595	0.027
H11C	–0.420169	–0.497545	–0.798323	0.026
H11D	–0.306305	–0.515832	–0.679344	0.026
H13D	–0.927761	–0.462699	–0.751445	0.039
H13E	–0.826099	–0.502841	–0.835956	0.039
H13F	–0.748677	–0.433441	–0.809917	0.039
H15C	–0.796861	–0.528523	–0.467303	0.028
H15D	–0.984289	–0.508538	–0.563552	0.028
H16C	–0.686694	–0.430786	–0.459057	0.029
H16D	–0.851495	–0.413597	–0.566767	0.029
H17	–0.429417	–0.459868	–0.550554	0.022
H18	–0.639623	–0.359038	–0.672035	0.027
H19D	–0.290431	–0.35809	–0.495152	0.048
H19E	–0.528413	–0.339414	–0.486405	0.048
H19F	–0.395241	–0.297658	–0.555207	0.048
H20D	–0.403661	–0.394516	–0.778883	0.044
H20E	–0.214735	–0.397441	–0.677232	0.044
H20F	–0.309058	–0.332178	–0.721591	0.044

Table SI34. Refined Thermal Parameters (U's) for Compound 21

Atom	U_{11}	U_{22}	U_{33}	U_{23}	U_{13}	U_{12}
O1*	0.0099(10)	0.0328(12)	0.022(1)	–0.0015(9)	0.0030(8)	–0.0002(8)
O2*	0.0210(12)	0.0387(13)	0.0211(11)	0.0004(10)	–0.0037(9)	–0.0028(10)
C1*	0.0144(13)	0.0249(14)	0.0112(12)	0.0017(10)	0.0025(10)	–0.0018(11)
C2*	0.0198(15)	0.0289(16)	0.0153(13)	0.0032(11)	0.0057(11)	–0.0045(12)
C3*	0.0231(15)	0.0240(15)	0.0204(14)	0.0066(11)	0.0003(12)	–0.0048(12)
C4*	0.0205(16)	0.0209(15)	0.0307(16)	0.0043(12)	0.0016(13)	–0.0025(12)
C5*	0.0226(17)	0.0293(17)	0.046(2)	0.0111(16)	0.0011(15)	0.0024(14)
C6*	0.0244(16)	0.0256(16)	0.0288(16)	0.0028(13)	0.0093(13)	0.0032(13)
C7*	0.0247(16)	0.0234(15)	0.0183(14)	–0.0015(11)	0.0067(12)	–0.0010(12)
C8*	0.0138(13)	0.0244(15)	0.0123(12)	–0.0001(11)	0.0036(10)	–0.0017(11)
C9*	0.0146(14)	0.0262(15)	0.0187(13)	0.0049(11)	–0.0004(11)	–0.0028(11)
C10*	0.0182(14)	0.0251(15)	0.0130(12)	0.0008(11)	0.0059(10)	0.0013(11)
C11*	0.0136(14)	0.0255(14)	0.0155(13)	–0.0011(11)	0.007(1)	–0.0010(11)
C12*	0.0134(13)	0.0250(14)	0.0101(11)	–0.0005(10)	0.0027(10)	0.0006(11)
C13*	0.0196(15)	0.0315(16)	0.0151(13)	0.0016(12)	0.0009(11)	0.0049(12)
C14*	0.0146(14)	0.0293(15)	0.0149(13)	0.0009(11)	0.0070(11)	0.0003(12)
C15*	0.0205(16)	0.0363(18)	0.0224(15)	–0.0025(13)	0.0142(12)	–0.0017(13)
C16*	0.0207(15)	0.0325(17)	0.0142(13)	–0.0012(12)	0.0072(11)	0.0019(12)
C17*	0.0126(13)	0.0254(14)	0.0137(12)	0.0002(10)	0.0048(10)	0.0030(11)
C18*	0.0188(15)	0.0254(15)	0.0181(14)	–0.0016(11)	0.0049(11)	0.0030(12)
C19*	0.0339(19)	0.0320(17)	0.0211(15)	–0.0066(13)	0.0047(13)	–0.0017(14)
C20*	0.0223(16)	0.0291(16)	0.0247(15)	–0.0029(13)	0.0063(12)	–0.0017(12)
O1	0.0156(10)	0.0355(13)	0.0185(10)	–0.0026(9)	0.0002(8)	–0.0049(9)
O2	0.0314(13)	0.0372(13)	0.0195(11)	–0.0026(10)	0.0027(10)	–0.0110(11)
C1	0.0164(14)	0.0267(15)	0.0127(12)	–0.0013(11)	0.0016(10)	–0.0044(11)
C2	0.0283(17)	0.0289(16)	0.0165(13)	0.0001(12)	0.0068(12)	–0.0068(13)
C3	0.0270(17)	0.0320(17)	0.0180(14)	0.0035(12)	0.0008(12)	–0.0098(13)
C4	0.0290(18)	0.0304(17)	0.0248(16)	0.0078(13)	0.0023(13)	–0.0062(14)
C5	0.0296(19)	0.046(2)	0.0331(19)	0.0125(17)	0.0017(15)	–0.0017(16)
C6	0.0322(19)	0.0330(18)	0.0249(16)	0.0026(14)	0.0079(14)	0.0035(14)
C7	0.0329(19)	0.0296(16)	0.0198(15)	–0.0022(12)	0.0080(13)	–0.0024(14)
C8	0.0211(15)	0.0288(16)	0.0146(13)	–0.0019(11)	0.0030(11)	–0.0035(12)
C9	0.0221(16)	0.0335(16)	0.0128(12)	0.0016(12)	0.0029(11)	–0.0074(13)
C10	0.0197(15)	0.0324(17)	0.0166(13)	–0.0008(12)	0.0080(11)	–0.0008(13)
C11	0.0170(14)	0.0335(16)	0.0154(13)	0.0012(12)	0.0070(11)	–0.0010(12)
C12	0.0161(14)	0.0272(15)	0.0134(12)	0.0001(11)	0.0031(10)	–0.0008(11)

C13	0.0195(15)	0.0388(18)	0.0186(14)	0.0022(13)	0.0024(12)	0.0024(13)
C14	0.0139(14)	0.0301(16)	0.0143(13)	–0.0009(11)	0.0040(11)	–0.0030(11)
C15	0.0145(14)	0.0397(18)	0.0162(13)	–0.0023(12)	0.0053(11)	–0.0018(12)
C16	0.0251(16)	0.0320(16)	0.0186(14)	–0.0003(12)	0.0106(12)	0.0040(13)
C17	0.0159(14)	0.0259(14)	0.0141(13)	0.0012(11)	0.0027(10)	0.0010(11)
C18	0.0223(16)	0.0258(15)	0.0213(14)	0.0028(12)	0.0071(12)	0.0019(13)
C19	0.044(2)	0.0265(16)	0.0269(17)	–0.0012(13)	0.0094(15)	–0.0017(15)
C20	0.0303(18)	0.0315(17)	0.0300(17)	0.0012(14)	0.0128(14)	–0.0047(14)

Table SI35. Bond Distances in Compound 21, Å

O1*-C9*	1.358(4)	O1*-C14*	1.474(4)	O2*-C9*	1.195(4)
C1*-C2*	1.535(4)	C1*-C8*	1.550(4)	C1*-C14*	1.540(4)
C2*-C3*	1.511(5)	C3*-C4*	1.330(5)	C4*-C5*	1.511(5)
C4*-C6*	1.517(5)	C6*-C7*	1.527(5)	C7*-C8*	1.532(4)
C8*-C9*	1.518(4)	C8*-C10*	1.552(4)	C10*-C11*	1.537(4)
C11*-C12*	1.545(4)	C12*-C13*	1.538(4)	C12*-C14*	1.572(4)
C12*-C17*	1.558(4)	C14*-C15*	1.537(4)	C15*-C16*	1.532(5)
C16*-C17*	1.541(4)	C17*-C18*	1.542(4)	C18*-C19*	1.537(4)
C18*-C20*	1.522(4)	O1-C9	1.358(4)	O1-C14	1.472(4)
O2-C9	1.201(4)	C1-C2	1.537(4)	C1-C8	1.541(4)
C1-C14	1.549(4)	C2-C3	1.511(5)	C3-C4	1.324(5)
C4-C5	1.504(5)	C4-C6	1.524(5)	C6-C7	1.524(5)
C7-C8	1.535(5)	C8-C9	1.515(5)	C8-C10	1.559(4)
C10-C11	1.543(5)	C11-C12	1.543(4)	C12-C13	1.537(4)
C12-C14	1.570(4)	C12-C17	1.563(4)	C14-C15	1.530(4)
C15-C16	1.535(5)	C16-C17	1.541(4)	C17-C18	1.544(4)
C18-C19	1.532(5)	C18-C20	1.525(5)		

Table SI36. Bond Angles in Compound 21, °

C9*-O1*-C14*	109.0(2)	C2*-C1*-C8*	112.0(2)	C2*-C1*-C14*	115.8(2)
C14*-C1*-C8*	98.2(2)	C3*-C2*-C1*	113.7(3)	C4*-C3*-C2*	128.6(3)
C3*-C4*-C5*	120.2(3)	C3*-C4*-C6*	126.0(3)	C5*-C4*-C6*	113.7(3)
C4*-C6*-C7*	114.0(3)	C6*-C7*-C8*	113.2(3)	C1*-C8*-C10*	110.1(2)
C7*-C8*-C1*	115.6(2)	C7*-C8*-C10*	112.7(2)	C9*-C8*-C1*	100.4(2)

C9*-C8*-C7*	110.3(2)	C9*-C8*-C10*	106.8(2)	O1*-C9*-C8*	109.1(2)
O2*-C9*-O1*	122.0(3)	O2*-C9*-C8*	128.9(3)	C11*-C10*-C8*	113.4(2)
C10*-C11*-C12*	115.1(2)	C11*-C12*-C14*	110.4(2)	C11*-C12*-C17*	113.0(2)
C13*-C12*-C11*	110.5(2)	C13*-C12*-C14*	110.1(2)	C13*-C12*-C17*	111.6(2)
C17*-C12*-C14*	100.9(2)	O1*-C14*-C1*	102.1(2)	O1*-C14*-C12*	110.8(2)
O1*-C14*-C15*	107.6(2)	C1*-C14*-C12*	111.1(2)	C15*-C14*-C1*	118.6(3)
C15*-C14*-C12*	106.4(2)	C16*-C15*-C14*	106.3(2)	C15*-C16*-C17*	106.6(2)
C16*-C17*-C12*	104.6(2)	C16*-C17*-C18*	111.4(2)	C18*-C17*-C12*	118.5(2)
C19*-C18*-C17*	109.3(3)	C20*-C18*-C17*	114.5(3)	C20*-C18*-C19*	109.2(3)
C9-O1-C14	109.2(2)	C2-C1-C8	112.0(3)	C2-C1-C14	115.1(3)
C8-C1-C14	98.5(2)	C3-C2-C1	113.5(3)	C4-C3-C2	129.5(3)
C3-C4-C5	121.0(3)	C3-C4-C6	125.0(3)	C5-C4-C6	113.9(3)
C7-C6-C4	114.5(3)	C6-C7-C8	113.4(3)	C1-C8-C10	110.4(3)
C7-C8-C1	115.7(3)	C7-C8-C10	112.6(3)	C9-C8-C1	100.1(3)
C9-C8-C7	110.6(3)	C9-C8-C10	106.3(2)	O1-C9-C8	109.2(2)
O2-C9-O1	121.3(3)	O2-C9-C8	129.5(3)	C11-C10-C8	112.8(2)
C10-C11-C12	115.2(3)	C11-C12-C14	110.5(3)	C11-C12-C17	113.2(2)
C13-C12-C11	110.3(2)	C13-C12-C14	109.9(3)	C13-C12-C17	112.0(3)
C17-C12-C14	100.6(2)	O1-C14-C1	102.3(2)	O1-C14-C12	110.8(2)
O1-C14-C15	108.3(2)	C1-C14-C12	110.3(2)	C15-C14-C1	118.9(3)
C15-C14-C12	106.1(3)	C14-C15-C16	106.8(3)	C15-C16-C17	106.0(3)
C16-C17-C12	104.2(2)	C16-C17-C18	111.2(3)	C18-C17-C12	119.6(2)
C19-C18-C17	109.3(3)	C20-C18-C17	115.0(3)	C20-C18-C19	108.6(3)

This report has been created with Olex2[12], compiled on 2022.04.07 svn.rca3783a0 for OlexSys.

X-ray Structure Determination of Azorellolide

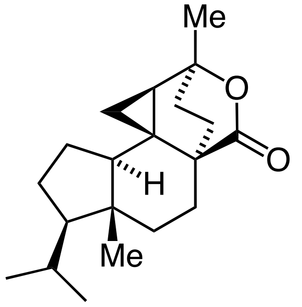

≡

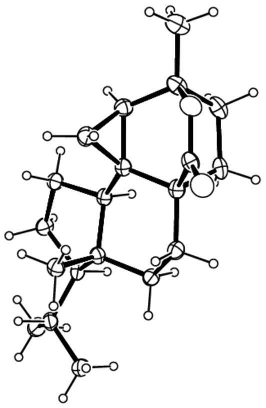

azorellolide
Synthetic CCDC: 2390968
Natural CCDC[5]: 249835

Azorellolide, $C_{20}H_{30}O_2$, crystallizes in the monoclinic space group $P2_1$ (systematic absences 0k0: k=odd) with a=6.9350(2)Å, b=9.2708(2) Å, c=13.2286(3) Å, α=90°, β=90.927(2)°, γ=90°, V=850.40(4) $Å^3$, Z=2, and d_{calc}=1.181 g/cm^3. X-ray intensity data were collected on a Rigaku XtaLAB Synergy-S diffractometer[7] equipped with an HPC area detector (HyPix-6000HE) and employing confocal multilayer optic-monochromated Cu-Kα radiation (λ=1.54184 Å) at a temperature of 100 K. Preliminary indexing was performed from a series of sixty 0.5° rotation frames with exposures of 0.625 seconds for θ = ±47.689° and 2.5 seconds for θ = 113.25°. A total of 3191 frames (41 runs) were collected employing ω scans with a crystal to detector distance of 34.0 mm, rotation widths of 0.5° and exposures of 4 seconds.

Rotation frames were integrated using CrysAlisPro[8] producing a listing of unaveraged F^2 and $\sigma(F^2)$ values. A total of 9477 reflections were measured over the ranges $6.682 \le 2\theta \le 148.876°$, $-8 \le h \le 8$, $-11 \le k \le 11$, $-16 \le l \le 16$ yielding 3291 unique reflections (R_{int} = 0.0529). The intensity data were corrected for Lorentz and polarization effects and for absorption using SCALE3 ABSPACK[9] (minimum and maximum transmission 0.54038, 1.00000). The structure was solved by dual space methods - SHELXT[10] Refinement was by full-matrix least squares based on F^2 using SHELXL[11]. All reflections were used during refinement. The weighting scheme used was

w=1/[$\sigma^2(F_o^2)$+ $(0.0438P)^2 + 0.1885P$] where $P = (F_o^2 + 2F_c^2)/3$. Non-hydrogen atoms were refined anisotropically and hydrogen atoms were refined using a riding model. Refinement converged to R1=0.0398 and wR2=0.0921 for 3031 observed reflections for which $F > 4\sigma(F)$ and R1=0.0447 and wR2=0.1033 and GOF =1.088 for all 3291 unique, non-zero reflections and 203 variables. The maximum Δ/σ in the final cycle of least squares was 0.000 and the two most prominent peaks in the final difference Fourier were +0.16 and –0.21 e/Å^3. **Table SI37**. lists cell information, data collection parameters, and refinement data. Final positional and equivalent isotropic thermal parameters are given in **Tables SI38**. and **SI39**. Anisotropic thermal parameters are in Table **SI40**. Tables 5 **SI41**. and 6 **SI42**. list bond distances and bond angles. **Figure 9**. is an ORTEP representation of Azorellolide with 50% probability thermal ellipsoids displayed.

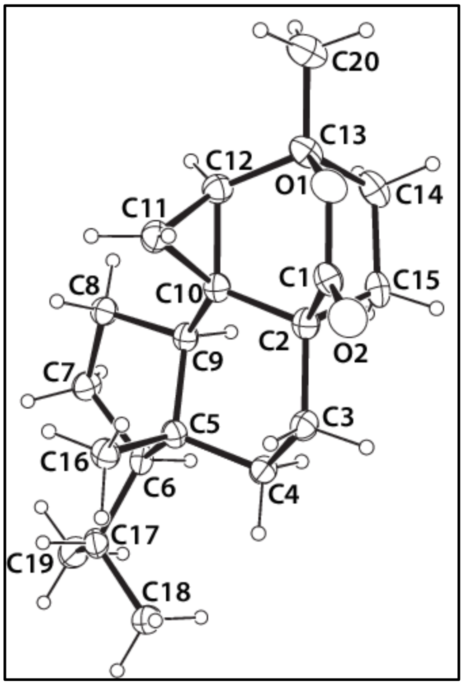

Figure 9. ORTEP drawing of **Azorellolide** with 50% thermal ellipsoids.

Table SI37. Summary of Structure Determination of Azorellolide

Empirical formula	$C_{20}H_{30}O_2$
Formula weight	302.44
Diffractometer	Rigaku XtaLAB Synergy-S (HyPix-6000HE)
Temperature/K	100
Crystal system	monoclinic
Space group	$P2_1$
a	6.9350(2) Å
b	9.2708(2) Å
c	13.2286(3) Å
α	90°
β	90.927(2)°
γ	90°
Volume	850.40(4)$Å^3$
Z	2
d_{calc}	1.181 g/cm^3
μ	0.570 mm^{-1}
F(000)	332.0
Crystal size, mm	0.21 × 0.04 × 0.02
2θ range for data collection	6.682 - 148.876°
Index ranges	–8 ≤ h ≤ 8, –11 ≤ k ≤ 11, –16 ≤ l ≤ 16
Reflections collected	9477
Independent reflections	3291[R(int) = 0.0529]
Data/restraints/parameters	3291/1/203
Goodness-of-fit on F^2	1.088
Final R indexes [I>=2σ (I)]	R_1 = 0.0398, wR_2 = 0.0921
Final R indexes [all data]	R_1 = 0.0447, wR_2 = 0.1033
Largest diff. peak/hole	0.16/–0.21 e$Å^{-3}$
Flack parameter	0.08(17)

Table SI38. Refined Positional Parameters for Compound Azorellolide

Atom	*x*	*y*	*z*	U(eq)
O1	0.4614(2)	0.4735(2)	–0.00518(13)	0.0240(4)
O2	0.7299(2)	0.4639(2)	0.08753(14)	0.0281(5)
C1	0.5558(3)	0.4588(3)	0.08489(18)	0.0210(5)
C2	0.4241(3)	0.4337(3)	0.17330(19)	0.0197(5)
C3	0.5419(3)	0.4220(3)	0.27236(19)	0.0204(5)
C4	0.4216(3)	0.4318(3)	0.36848(19)	0.0200(5)
C5	0.2906(3)	0.5652(3)	0.36921(18)	0.0185(5)
C6	0.1346(3)	0.5682(3)	0.45167(18)	0.0192(5)
C7	–0.0330(4)	0.6585(3)	0.40327(19)	0.0219(5)
C8	0.0071(3)	0.6717(3)	0.28946(18)	0.0205(5)
C9	0.1617(3)	0.5572(3)	0.27299(18)	0.0179(5)
C10	0.2753(3)	0.5578(3)	0.17706(18)	0.0183(5)
C11	0.3107(4)	0.6930(3)	0.11572(19)	0.0223(5)
C12	0.1801(4)	0.5769(3)	0.07499(19)	0.0212(5)
C13	0.2498(3)	0.4650(3)	0.00045(19)	0.0227(5)
C14	0.2024(4)	0.3161(3)	0.0433(2)	0.0260(6)
C15	0.3138(4)	0.2933(3)	0.1445(2)	0.0226(6)
C16	0.4101(4)	0.7049(3)	0.37198(19)	0.0210(5)
C17	0.1881(4)	0.6229(3)	0.55870(19)	0.0216(6)
C18	0.3559(4)	0.5406(3)	0.6080(2)	0.0270(6)
C19	0.0135(4)	0.6141(3)	0.6276(2)	0.0294(6)
C20	0.1758(4)	0.4901(4)	–0.1062(2)	0.0327(7)
O1	0.4614(2)	0.4735(2)	–0.00518(13)	0.0240(4)

Table SI39 . Positional Parameters for Hydrogens in Azorellolide.

Atom	*x*	*y*	*z*	U(eq)
H3A	0.611831	0.328951	0.272785	0.025
H3B	0.639372	0.500046	0.273915	0.025
H4A	0.341115	0.344088	0.373999	0.024
H4B	0.509447	0.43506	0.428182	0.024
H6	0.086046	0.467261	0.459156	0.023
H7A	–0.158125	0.609774	0.413761	0.026

H7B	–0.038166	0.755357	0.43465	0.026
H8A	–0.110317	0.651318	0.248361	0.025
H8B	0.055304	0.769149	0.272466	0.025
H9	0.094385	0.461858	0.27536	0.021
H11A	0.250636	0.783813	0.138779	0.027
H11B	0.438528	0.704417	0.084467	0.027
H12	0.038699	0.598029	0.074131	0.025
H14A	0.239305	0.240471	–0.005596	0.031
H14B	0.062024	0.308426	0.054538	0.031
H15A	0.406169	0.212609	0.137722	0.027
H15B	0.222216	0.26835	0.198471	0.027
H16A	0.488349	0.710908	0.31109	0.031
H16B	0.494818	0.704729	0.431995	0.031
H16C	0.323288	0.788208	0.37461	0.031
H17	0.22654	0.726565	0.553169	0.026
H18A	0.327601	0.43706	0.607169	0.04
H18B	0.373723	0.573075	0.678018	0.04
H18C	0.474003	0.558959	0.57036	0.04
H19A	0.047711	0.653662	0.694255	0.044
H19B	–0.025904	0.513191	0.634853	0.044
H19C	–0.093285	0.669948	0.597966	0.044
H20A	0.213386	0.586704	–0.128537	0.049
H20B	0.034848	0.481967	–0.107936	0.049
H20C	0.231047	0.417738	–0.151352	0.049
H3A	0.611831	0.328951	0.272785	0.025
H3B	0.639372	0.500046	0.273915	0.025

Table SI40 . Refined Thermal Parameters (U's) for Azorellolide.

Atom	U_{11}	U_{22}	U_{33}	U_{23}	U_{13}	U_{12}
O1	0.0200(9)	0.0312(11)	0.0211(9)	–0.0025(8)	0.0040(7)	–0.0023(8)
O2	0.0198(9)	0.0340(12)	0.0308(10)	–0.0025(9)	0.0044(7)	–0.0015(8)
C1	0.0213(12)	0.0209(14)	0.0209(13)	–0.0041(10)	0.0043(9)	–0.0008(10)
C2	0.0181(11)	0.0175(13)	0.0237(13)	–0.0007(10)	0.0016(9)	–0.0013(10)
C3	0.0196(12)	0.0199(13)	0.0219(13)	0.0003(10)	0.0014(9)	0.0026(10)
C4	0.0203(11)	0.0186(13)	0.0212(13)	0.0006(10)	–0.0011(9)	0.0009(10)
C5	0.0190(11)	0.0165(12)	0.0198(12)	–0.0015(10)	0.0002(9)	–0.0009(10)
C6	0.0210(12)	0.0147(12)	0.0221(12)	0.0003(10)	0.0034(9)	0.0018(10)
C7	0.0192(12)	0.0246(14)	0.0221(13)	0.0003(10)	0.0026(9)	0.0031(10)
C8	0.0198(12)	0.0208(13)	0.0208(12)	–0.001(1)	0.0003(9)	0.002(1)
C9	0.0158(11)	0.0160(12)	0.0219(13)	–0.0008(10)	0.0009(9)	–0.0007(10)
C10	0.0168(11)	0.0175(12)	0.0207(12)	–0.0009(10)	0.0010(9)	–0.0008(10)
C11	0.0257(13)	0.0186(14)	0.0227(13)	0.0006(10)	0.0022(9)	0.0013(10)
C12	0.0213(12)	0.0227(14)	0.0198(12)	0.0008(11)	0.0011(9)	0.0001(11)
C13	0.0178(11)	0.0295(15)	0.0209(13)	–0.0053(11)	0.0032(9)	–0.0026(11)
C14	0.0225(13)	0.0274(15)	0.0283(15)	0.0105(11)	0.0036(11)	–0.0050(11)
C15	0.0257(14)	0.0151(13)	0.0271(14)	–0.0038(10)	0.0061(10)	–0.0019(10)
C16	0.0198(12)	0.0192(14)	0.0238(13)	–0.0005(10)	–0.0001(9)	–0.0026(10)
C17	0.0275(13)	0.0171(13)	0.0203(13)	–0.0021(10)	0.0019(10)	0.0016(10)
C18	0.0325(14)	0.0269(15)	0.0214(13)	–0.0008(10)	–0.0027(10)	0.0069(12)
C19	0.0337(16)	0.0306(16)	0.0239(14)	0.0014(11)	0.0035(11)	0.0077(12)
C20	0.0316(15)	0.0419(18)	0.0246(15)	–0.0059(13)	–0.0017(11)	–0.0017(13)
O1	0.0200(9)	0.0312(11)	0.0211(9)	–0.0025(8)	0.0040(7)	–0.0023(8)

Table SI41. Bond Distances in Azorellolide, Å

O1-C1	1.357(3)	O1-C13	1.473(3)	O2-C1	1.208(3)
C1-C2	1.514(3)	C2-C3	1.537(3)	C2-C10	1.547(4)
C2-C15	1.554(3)	C3-C4	1.535(4)	C4-C5	1.535(4)
C5-C6	1.549(3)	C5-C9	1.546(3)	C5-C16	1.538(4)
C6-C7	1.561(3)	C6-C17	1.544(3)	C7-C8	1.540(4)
C8-C9	1.527(3)	C9-C10	1.505(3)	C10-C11	1.515(4)
C10-C12	1.504(3)	C11-C12	1.501(4)	C12-C13	1.515(4)
C13-C14	1.531(4)	C13-C20	1.512(4)	C14-C15	1.550(4)
C17-C18	1.529(4)	C17-C19	1.530(4)		

Table SI42. Bond Angles in Compound Azorellolide, °

C1-O1-C13	114.65(18)	O1-C1-C2	113.9(2)	O2-C1-O1	119.3(2)
O2-C1-C2	126.8(2)	C1-C2-C3	110.53(19)	C1-C2-C10	108.7(2)
C1-C2-C15	103.9(2)	C3-C2-C10	111.7(2)	C3-C2-C15	113.7(2)
C10-C2-C15	107.75(19)	C4-C3-C2	114.5(2)	C5-C4-C3	112.5(2)
C4-C5-C6	116.1(2)	C4-C5-C9	106.9(2)	C4-C5-C16	111.12(19)
C9-C5-C6	100.32(18)	C16-C5-C6	110.5(2)	C16-C5-C9	111.4(2)
C5-C6-C7	104.17(19)	C17-C6-C5	119.4(2)	C17-C6-C7	111.5(2)
C8-C7-C6	107.4(2)	C9-C8-C7	102.8(2)	C8-C9-C5	104.30(19)
C10-C9-C5	112.98(19)	C10-C9-C8	119.7(2)	C9-C10-C2	112.5(2)
C9-C10-C11	123.1(2)	C11-C10-C2	119.0(2)	C12-C10-C2	110.0(2)
C12-C10-C9	122.0(2)	C12-C10-C11	59.62(17)	C12-C11-C10	59.83(17)
C10-C12-C13	111.3(2)	C11-C12-C10	60.55(17)	C11-C12-C13	121.8(2)
O1-C13-C12	109.0(2)	O1-C13-C14	106.7(2)	O1-C13-C20	105.6(2)
C12-C13-C14	107.7(2)	C20-C13-C12	113.3(2)	C20-C13-C14	114.4(2)
C13-C14-C15	109.6(2)	C14-C15-C2	109.5(2)	C18-C17-C6	113.4(2)
C18-C17-C19	108.9(2)	C19-C17-C6	110.4(2)		

This report has been created with Olex2[12] compiled on 2021.10.07 svn.r9718050b for OlexSys.

References

(1) Stambulyan, H.; Minehan, T. G. A protecting group-free synthesis of (–)-hortonones A–C from the Inhoffen–Lythgoe diol. *Organic & Biomolecular Chemistry* **2016**, *14* (37), 8728-8731. DOI: 10.1039/c6ob01738j.

(2) Fieser, L. F.; Fieser, M. *Reagents for organic synthesis*; Wiley, 1967.

(3) Haider, M.; Sennari, G.; Eggert, A.; Sarpong, R. Total Synthesis of the Cephalotaxus Norditerpenoids (+/-)-Cephanolides A-D. *J Am Chem Soc* **2021**, *143* (7), 2710-2715. DOI: 10.1021/jacs.1c00293 From NLM Medline.

(4) Hafeman, N. J.; Chan, M.; Fulton, T. J.; Alexy, E. J.; Loskot, S. A.; Virgil, S. C.; Stoltz, B. M. Asymmetric Total Synthesis of Havellockate. *J Am Chem Soc* **2022**, *144* (44), 20232-20236. DOI: 10.1021/jacs.2c09583 From NLM Medline.

(5) Colloca, C. B.; Pappano, D. B.; Bustos, D. A.; Sosa, V. E.; Baggio, R. F.; Garland, M. T.; Gil, R. R. Azorellane diterpenes from Azorella cryptantha. *Phytochemistry* **2004**, *65* (14), 2085-2089. DOI: 10.1016/j.phytochem.2004.03.038 From NLM Medline.

(6) Lima, B.; Sanchez, M.; Agüero, M. B.; Tapia, A.; Palermo, J. A.; Feresin, G. E. Antibacterial activity of extracts and compounds isolated from the Andean medicinal plant Azorella cryptantha (Clos) Reiche, Apiaceae. *Industrial Crops and Products* **2015**, *64*, 152-157. DOI: https://doi.org/10.1016/j.indcrop.2014.10.065.

(7) CrysAlisPro 1.171.43.106a: Rigaku Oxford Diffraction, Rigaku Corporation, Oxford, UK. (2024).

(8) CrysAlisPro 1.171.43.106a: Rigaku Oxford Diffraction, Rigaku Corporation, Oxford, UK. (2024).

(9) SCALE3 ABSPACK v1.0.7: an Oxford Diffraction program; Oxford Diffraction Ltd: Abingdon, UK, 2005.

(10) SHELXT v2018/2: Sheldrick, G.M., Acta Cryst., A, 71, 3-8 (2015).

(11) SHELXL-2019/3: Sheldrick, G.M., Acta Cryst., A, 71, 3-8 (2015).

(12) Olex2: Dolomanov, O. V.; Bourhis, L. J.; Gildea, R. J.; Howard, J. A. K.; Puschmann, H., *J. Appl. Cryst.* **2009**, *42*, 339-341
